# Supplementary material for: Local adaptation to climate anomalies relates to species phylogeny
Source: Commun Biol. 2022 Feb 17;5:143. doi: 10.1038/s42003-022-03088-3 (PMC8854402; doi:10.1038/s42003-022-03088-3)
Supplement: Supplementary file 2 — Supplementary Information [file 42003_2022_3088_MOESM2_ESM.pdf]

Local adaptation to climate anomalies relates to species phylogeny

Yolanda Melero, Luke C. Evans, Mikko Kuussaari, Reto Schmucki, Constantí Stefanescu,

David B. Roy, Tom H. Oliver

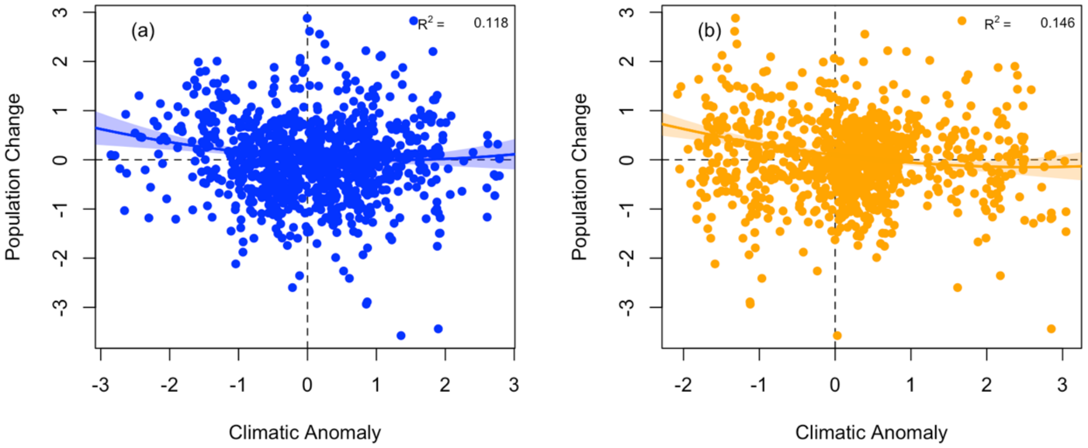

Supplementary Figure 1. Population change in relation to local and global climatic anomalies for (a) local and (b) global responses respectively for *Aglais io*, a species best adapted to global climatic anomalies in temperature during the post flight period of the previous year (t-1) of their adult stage. Colors indicate spatial scale (blue, local; orange, global), circles indicate raw data. R^2 values are provided.

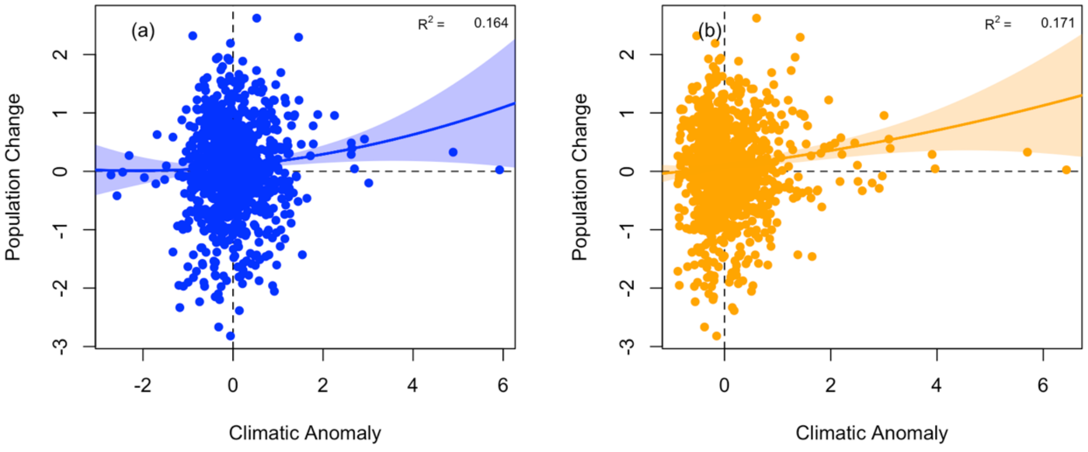

Supplementary Figure 2. Population change in relation to local and global climatic anomalies for (a) local and (b) global responses respectively for *Anthocharis cardamines*, a species best adapted to global climatic anomalies in precipitation during the flight period of the previous year (t-1) of their adult stage. Colors indicate spatial scale (blue, local; orange, global), circles indicate raw data. R^2 values are provided.

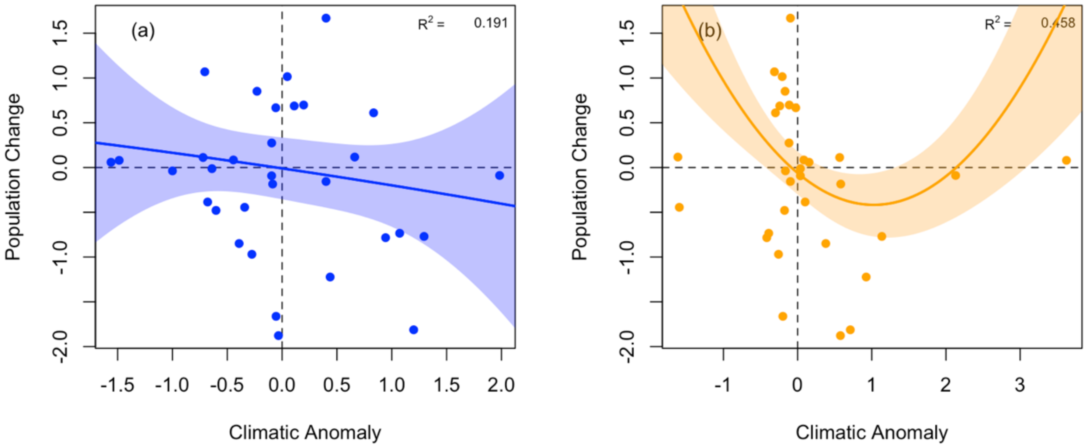

Supplementary Figure 3. Population change in relation to local and global climatic anomalies for (a) local and (b) global responses respectively for *Apatura ilia*, a species best adapted to global climatic anomalies in aridity during the pre-flight period of the previous year (t-1) of their adult stage. Colors indicate spatial scale (blue, local; orange, global), circles indicate raw data. R^2 values are provided.

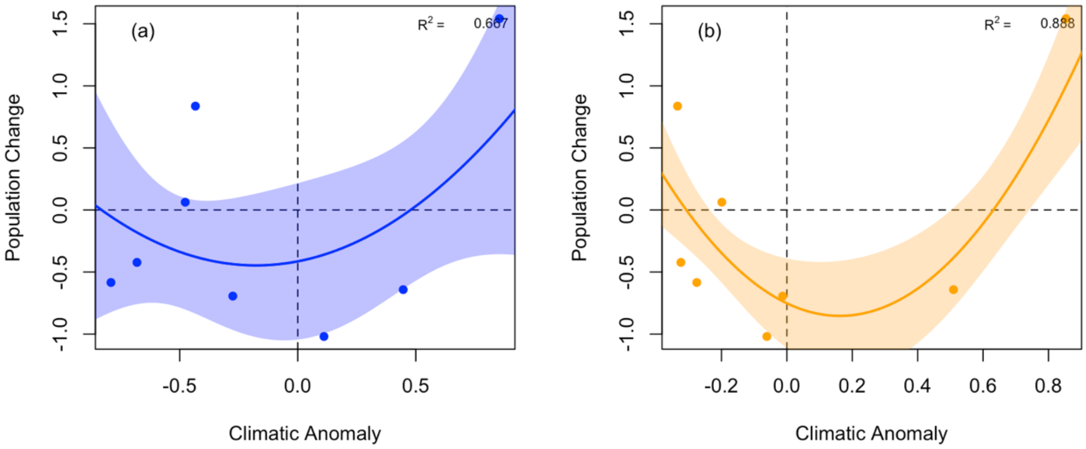

Supplementary Figure 4. Population change in relation to local and global climatic anomalies for (a) local and (b) global responses respectively for *Apatura iris*, a species best adapted to global climatic anomalies in precipitation during the flight period of the year (t) of their adult stage. Colors indicate spatial scale (blue, local; orange, global), circles indicate raw data. R^2 values are provided.

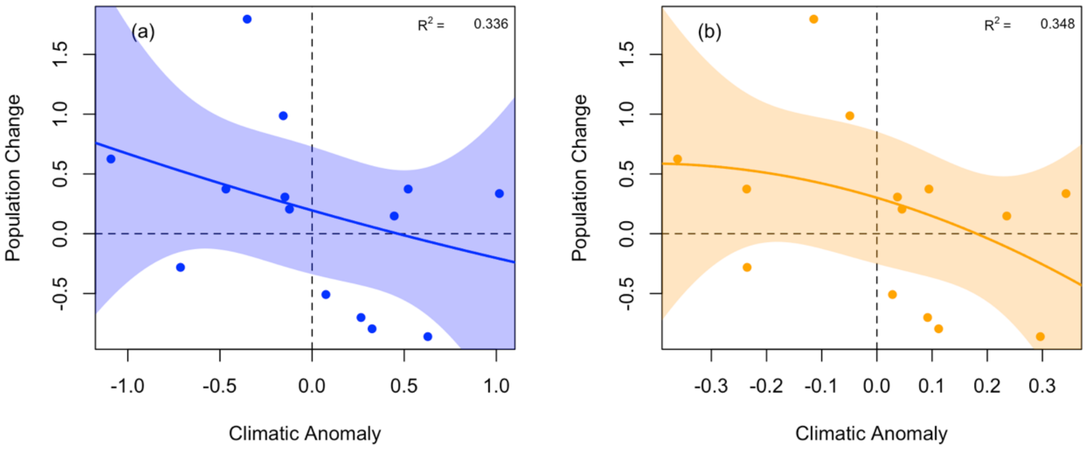

Supplementary Figure 5. Population change in relation to local and global climatic anomalies for (a) local and (b) global responses respectively for *Arethusana arethusa*, a species best adapted to global climatic anomalies in temperature during the pre-flight period of the year (t) of their adult stage. Colors indicate spatial scale (blue, local; orange, global), circles indicate raw data. R^2 values are provided.

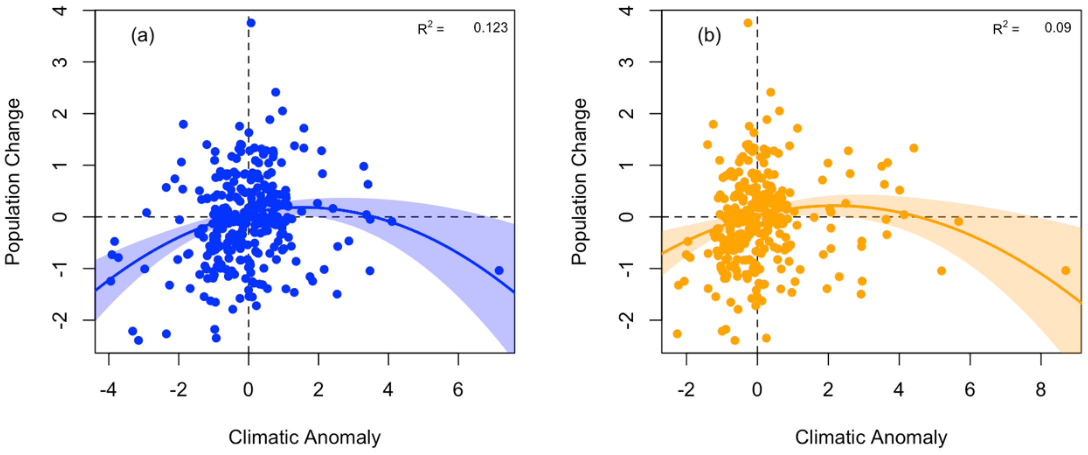

Supplementary Figure 6. Population change in relation to local and global climatic anomalies for (a) local and (b) global responses respectively for *Argynnis adippe*, a species best adapted to local climatic anomalies in precipitation during the pre-flight period of the previous year (t-1) of their adult stage. Colors indicate spatial scale (blue, local; orange, global), circles indicate raw data. R^2 values are provided.

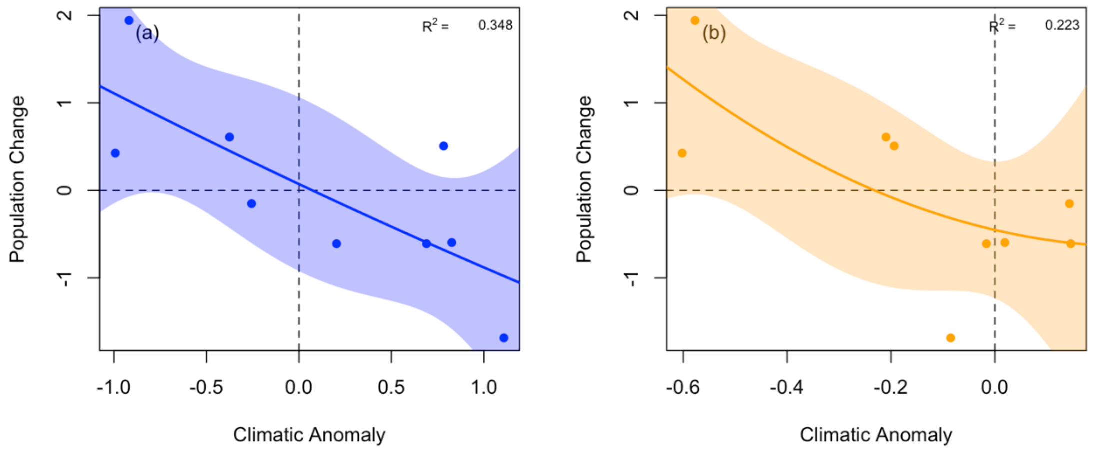

Supplementary Figure 7. Population change in relation to local and global climatic anomalies for (a) local and (b) global responses respectively for *Argynnis pandora*, a species best adapted to local climatic anomalies in temperature during the flight period of the year (t) of their adult stage. Colors indicate spatial scale (blue, local; orange, global), circles indicate raw data. R^2 values are provided.

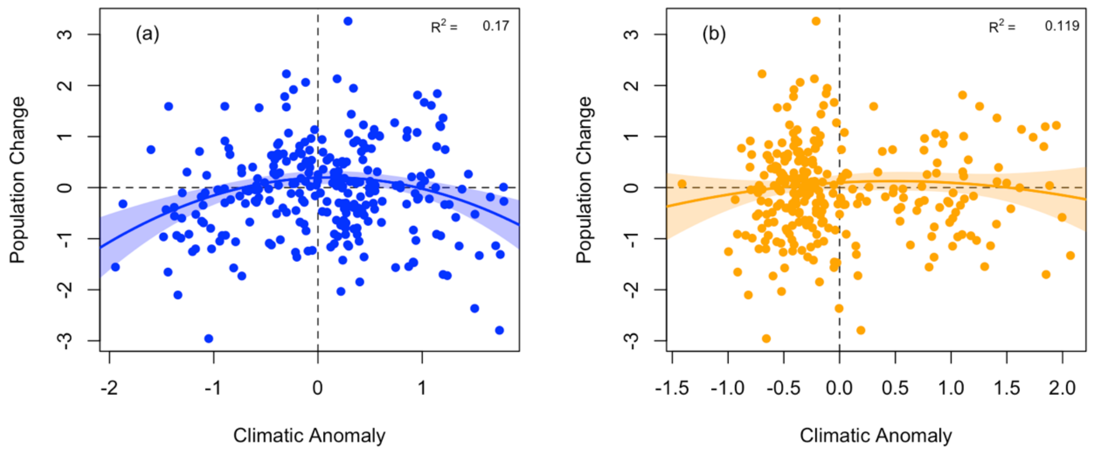

Supplementary Figure 8. Population change in relation to local and global climatic anomalies for (a) local and (b) global responses respectively for *Aricia agestis*, a species best adapted to local climatic anomalies in temperature during the flight period of the previous year (t-1) of their adult stage. Colors indicate spatial scale (blue, local; orange, global), circles indicate raw data. R^2 values are provided.

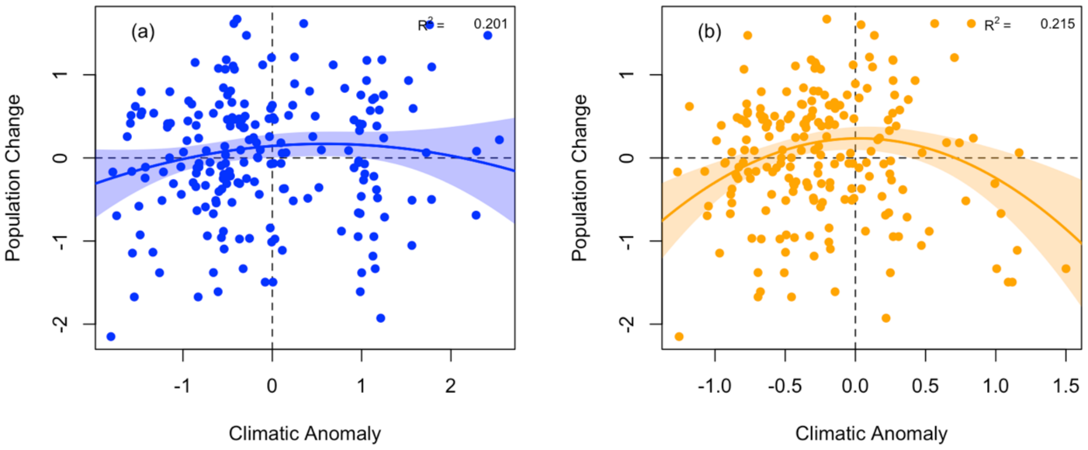

Supplementary Figure 9. Population change in relation to local and global climatic anomalies for (a) local and (b) global responses respectively for *Aricia artaxerxes*, a species best adapted to global climatic anomalies in temperature during the pre-flight period of the year (t) of their adult stage. Colors indicate spatial scale (blue, local; orange, global), circles indicate raw data. R^2 values are provided.

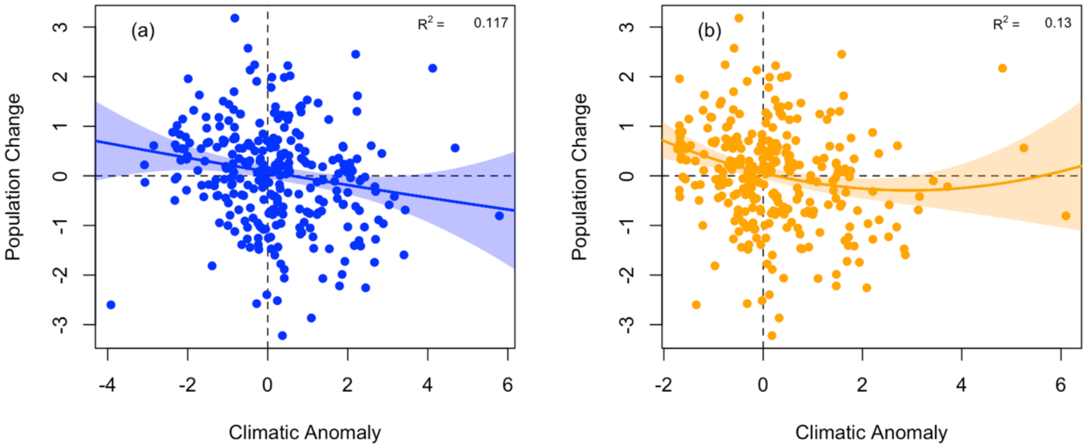

Supplementary Figure 10. Population change in relation to local and global climatic anomalies for (a) local and (b) global responses respectively for *Aricia cramera*, a species best adapted to global climatic anomalies in precipitation during the pre-flight period of the previous year (t-1) of their adult stage. Colors indicate spatial scale (blue, local; orange, global), circles indicate raw data. R^2 values are provided.

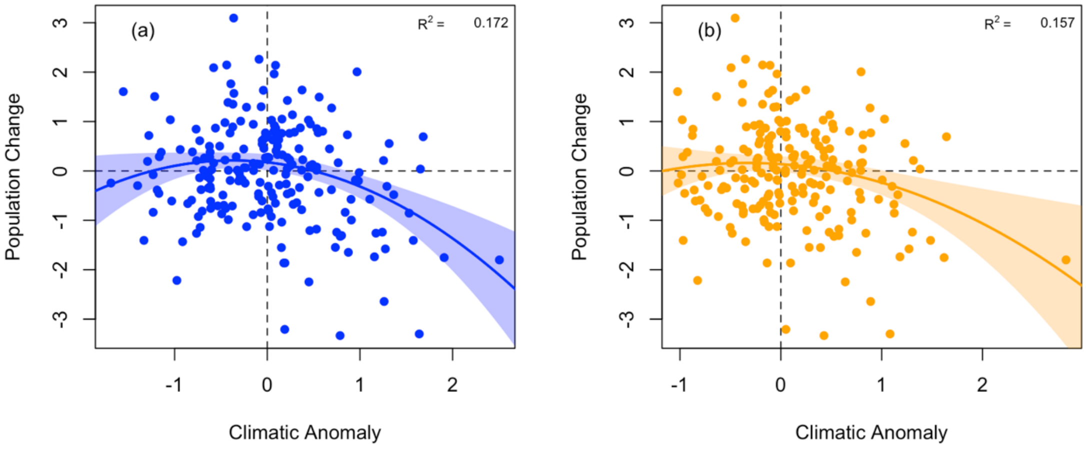

Supplementary Figure 11. Population change in relation to local and global climatic anomalies for (a) local and (b) global responses respectively for *Boloria dia*, a species best adapted to local climatic anomalies in precipitation during the post flight period of the previous year (t-1) of their adult stage. Colors indicate spatial scale (blue, local; orange, global), circles indicate raw data. R^2 values are provided.

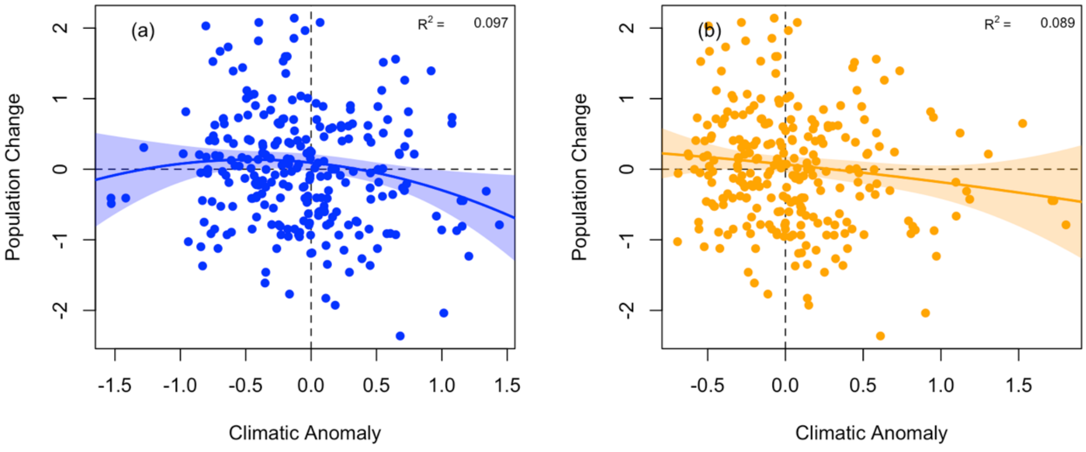

Supplementary Figure 12. Population change in relation to local and global climatic anomalies for (a) local and (b) global responses respectively for *Boloria euphrosyne*, a species best adapted to local climatic anomalies in precipitation during the flight period of the year (t) of their adult stage. Colors indicate spatial scale (blue, local; orange, global), circles indicate raw data. R^2 values are provided.

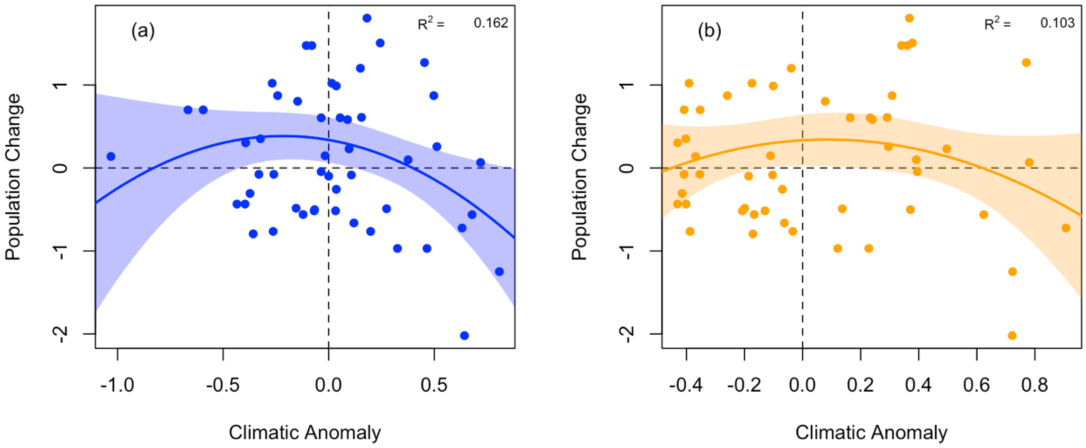

Supplementary Figure 13. Population change in relation to local and global climatic anomalies for (a) local and (b) global responses respectively for *Brenthis daphne*, a species best adapted to local climatic anomalies in precipitation during the flight period of the year (t) of their adult stage. Colors indicate spatial scale (blue, local; orange, global), circles indicate raw data. R^2 values are provided.

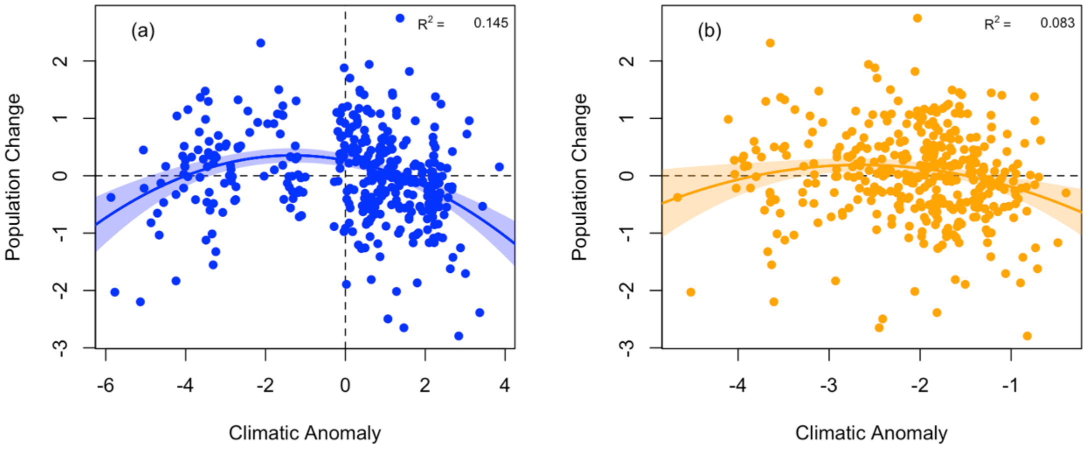

Supplementary Figure 14. Population change in relation to local and global climatic anomalies for (a) local and (b) global responses respectively for *Brenthis ino*, a species best adapted to local climatic anomalies in temperature during the overwintering of the previous year (t-1) of their adult stage. Colors indicate spatial scale (blue, local; orange, global), circles indicate raw data. R^2 values are provided.

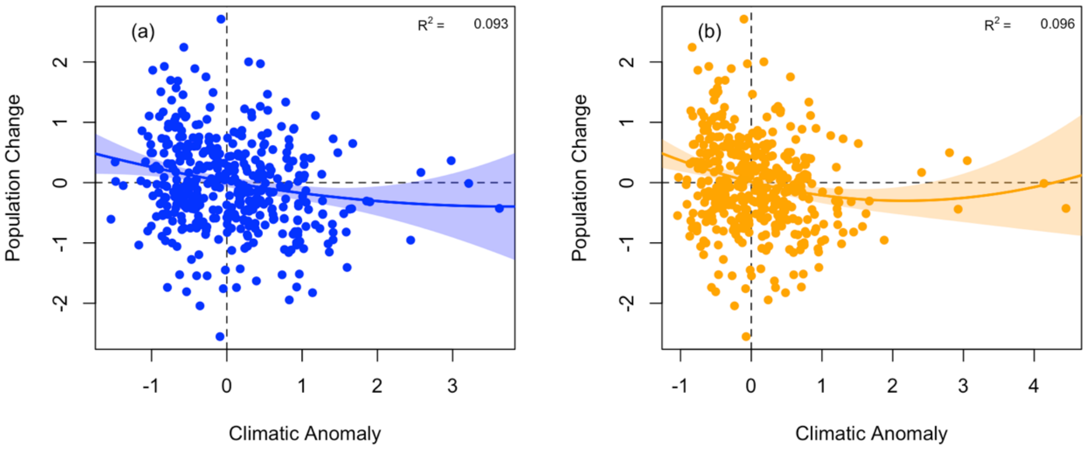

Supplementary Figure 15. Population change in relation to local and global climatic anomalies for (a) local and (b) global responses respectively for *Brintesia circe*, a species best adapted to global climatic anomalies in precipitation during the flight period of the previous year (t-1) of their adult stage. Colors indicate spatial scale (blue, local; orange, global), circles indicate raw data. R^2 values are provided.

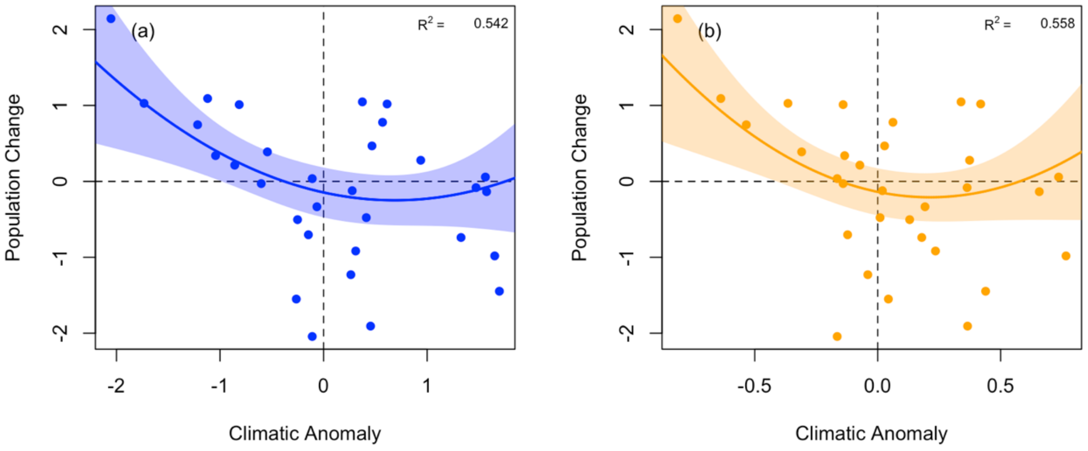

Supplementary Figure 16. Population change in relation to local and global climatic anomalies for (a) local and (b) global responses respectively for *Cacyreus marshalli*, a species best adapted to global climatic anomalies in temperature during the flight period of the previous year (t-1) of their adult stage. Colors indicate spatial scale (blue, local; orange, global), circles indicate raw data. R^2 values are provided.

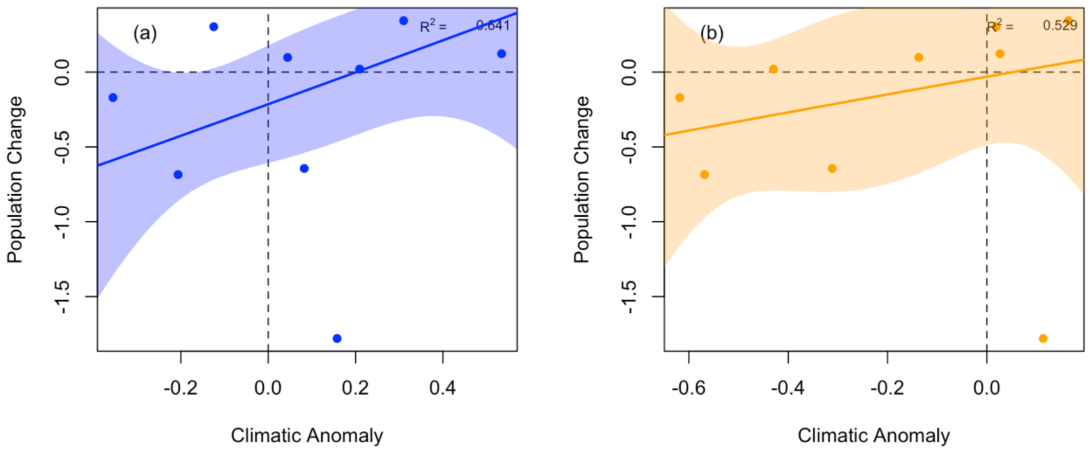

Supplementary Figure 17. Population change in relation to local and global climatic anomalies for (a) local and (b) global responses respectively for *Callophrys avis*, a species best adapted to local climatic anomalies in temperature during the post flight period of the previous year (t-1) of their adult stage. Colors indicate spatial scale (blue, local; orange, global), circles indicate raw data. R^2 values are provided.

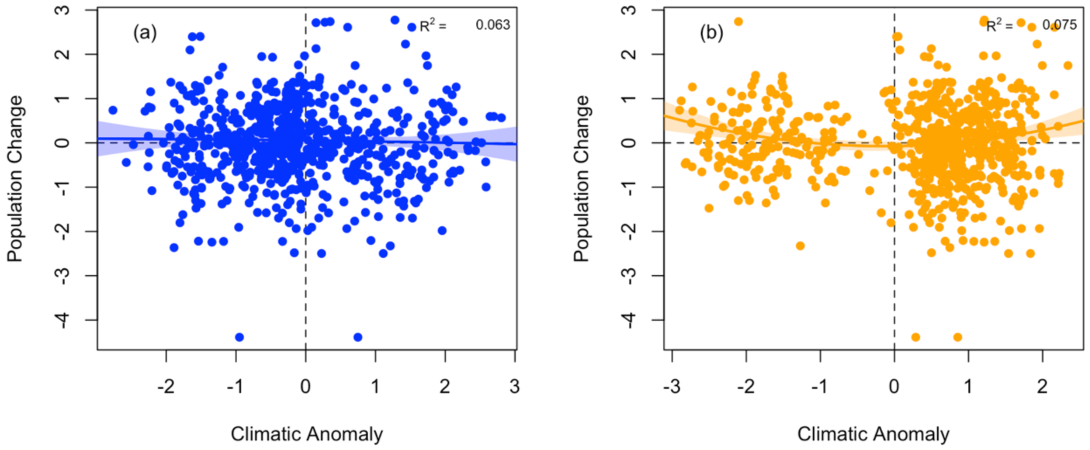

Supplementary Figure 18. Population change in relation to local and global climatic anomalies for (a) local and (b) global responses respectively for *Callophrys rubi*, a species best adapted to global climatic anomalies in temperature during the pre-flight period of the year (t) of their adult stage. Colors indicate spatial scale (blue, local; orange, global), circles indicate raw data. R^2 values are provided.

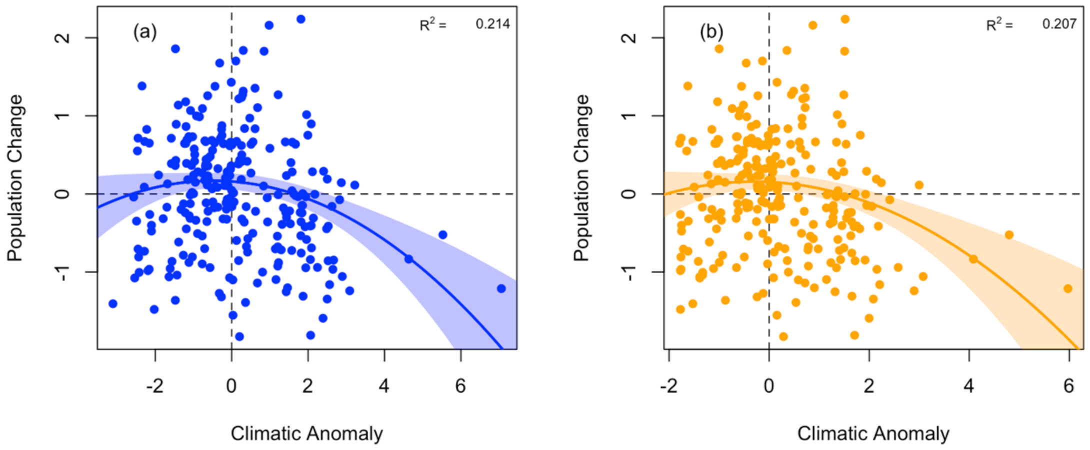

Supplementary Figure 19. Population change in relation to local and global climatic anomalies for (a) local and (b) global responses respectively for *Charaxes jasius*, a species best adapted to local climatic anomalies in precipitation during the pre-flight period of the previous year (t-1) of their adult stage. Colors indicate spatial scale (blue, local; orange, global), circles indicate raw data. R^2 values are provided.

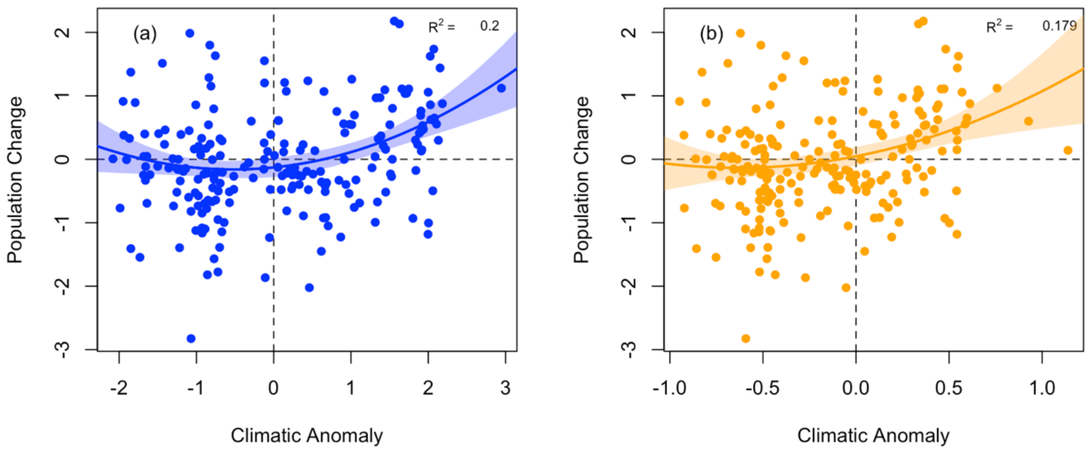

Supplementary Figure 20. Population change in relation to local and global climatic anomalies for (a) local and (b) global responses respectively for *Coenonympha glycerion*, a species best adapted to local climatic anomalies in temperature during the flight period of the previous year (t-1) of their adult stage. Colors indicate spatial scale (blue, local; orange, global), circles indicate raw data. R^2 values are provided.

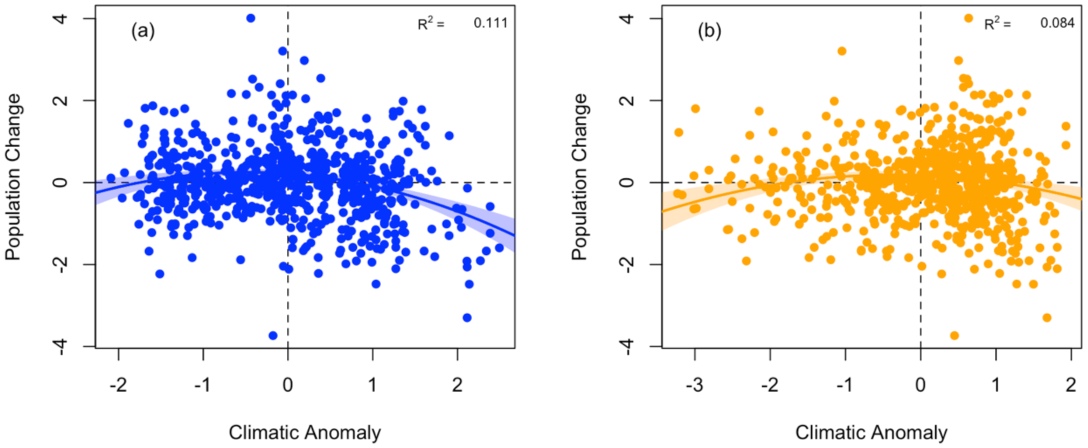

Supplementary Figure 21. Population change in relation to local and global climatic anomalies for (a) local and (b) global responses respectively for *Colias croceus*, a species best adapted to local climatic anomalies in temperature during the post flight period of the previous year (t-1) of their adult stage. Colors indicate spatial scale (blue, local; orange, global), circles indicate raw data. R^2 values are provided.

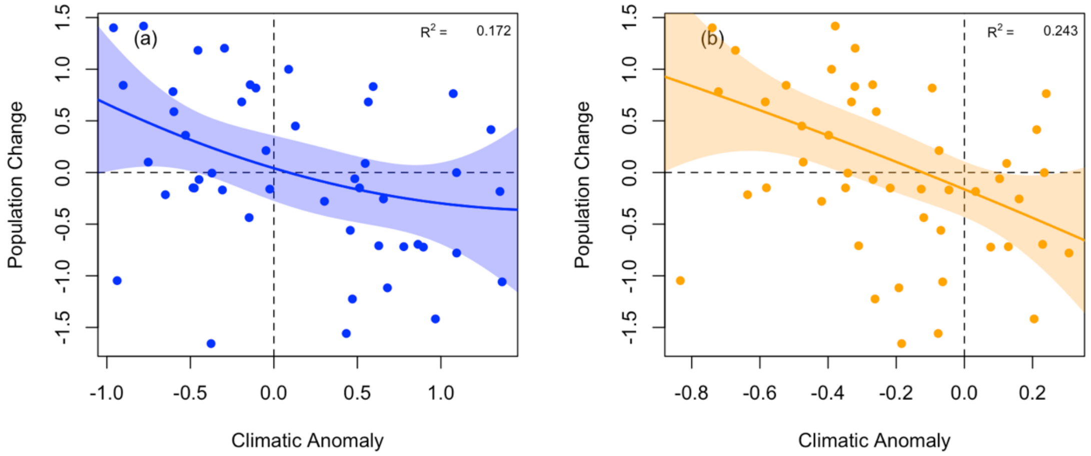

Supplementary Figure 22. Population change in relation to local and global climatic anomalies for (a) local and (b) global responses respectively for *Colias palaeno*, a species best adapted to global climatic anomalies in temperature during the post flight period of the previous year (t-1) of their adult stage. Colors indicate spatial scale (blue, local; orange, global), circles indicate raw data. R^2 values are provided.

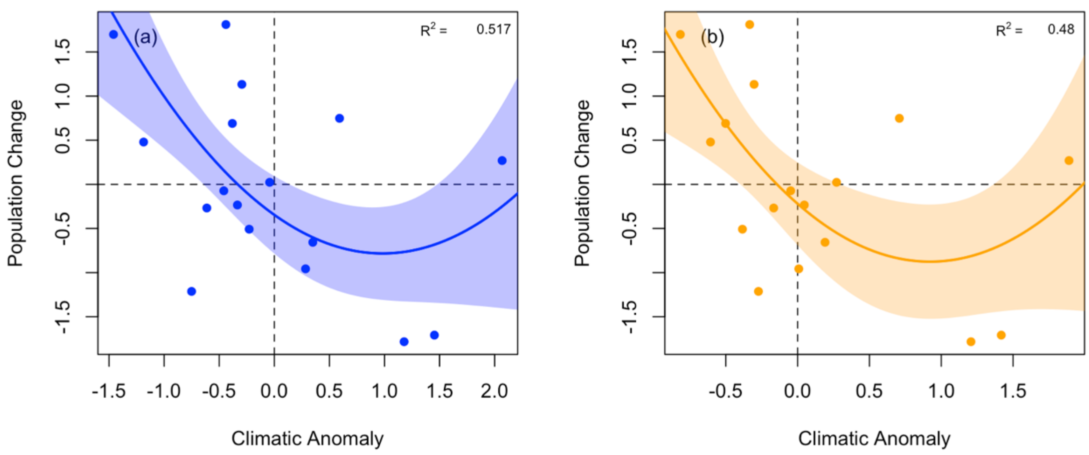

Supplementary Figure 23. Population change in relation to local and global climatic anomalies for (a) local and (b) global responses respectively for *Cupido alcetas*, a species best adapted to local climatic anomalies in precipitation during the post flight period of the previous year (t-1) of their adult stage. Colors indicate spatial scale (blue, local; orange, global), circles indicate raw data. R^2 values are provided.

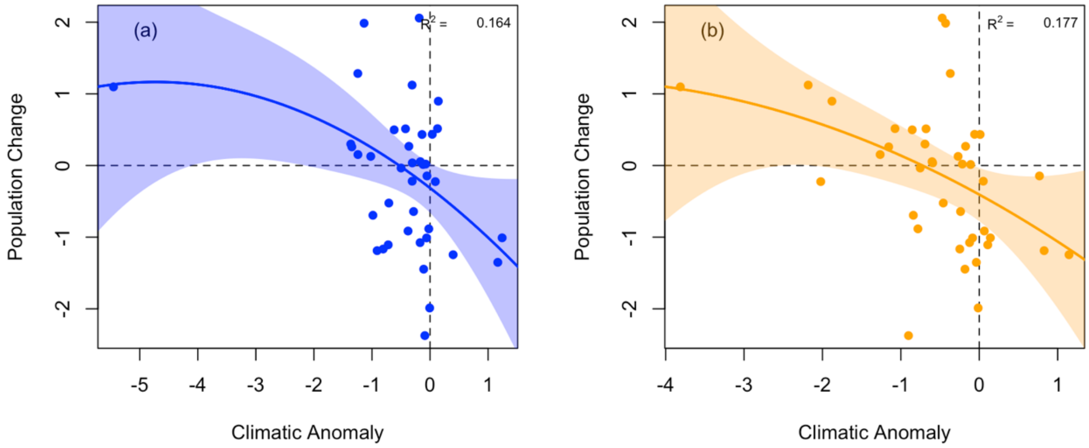

Supplementary Figure 24. Population change in relation to local and global climatic anomalies for (a) local and (b) global responses respectively for *Cupido argiades*, a species best adapted to global climatic anomalies in aridity during the pre-flight period of the previous year (t-1) of their adult stage. Colors indicate spatial scale (blue, local; orange, global), circles indicate raw data. R^2 values are provided.

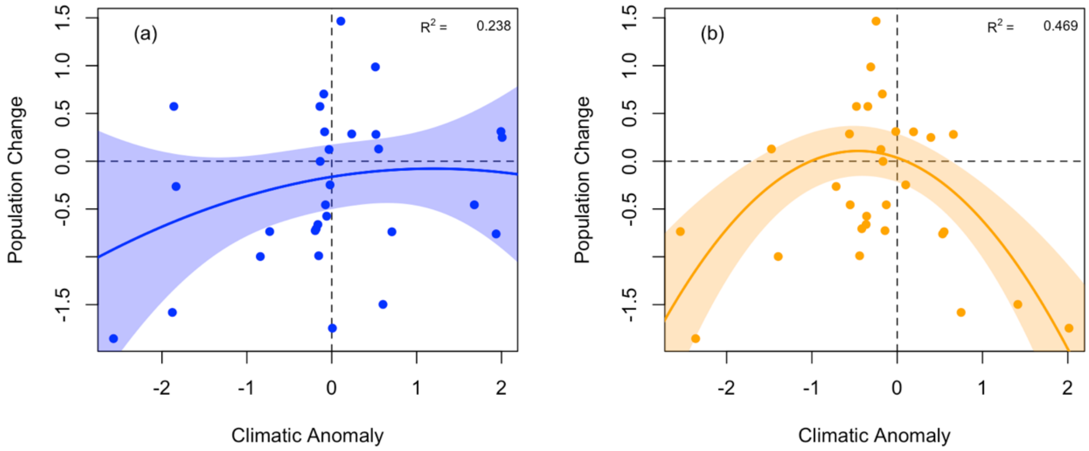

Supplementary Figure 25. Population change in relation to local and global climatic anomalies for (a) local and (b) global responses respectively for *Cupido osiris*, a species best adapted to global climatic anomalies in aridity during the pre-flight period of the year (t) of their adult stage. Colors indicate spatial scale (blue, local; orange, global), circles indicate raw data. R2 values are provided.

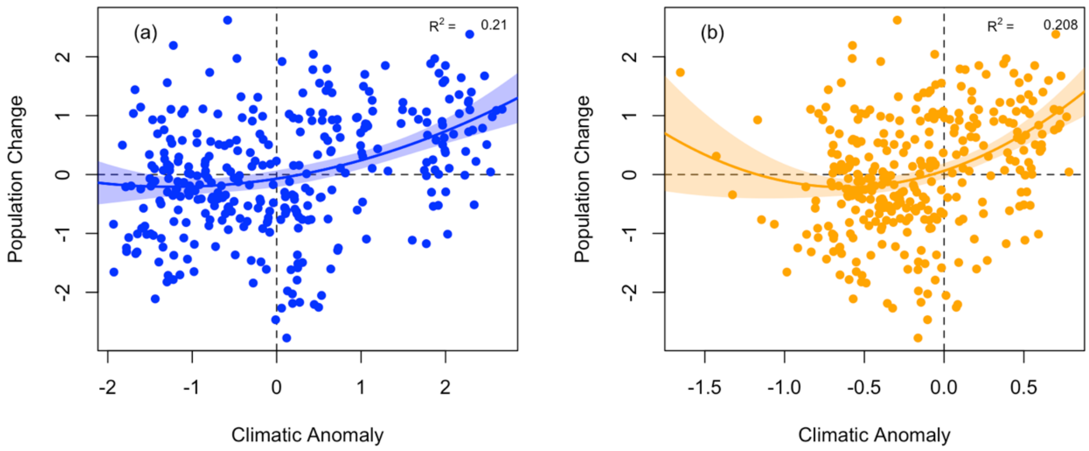

Supplementary Figure 26. Population change in relation to local and global climatic anomalies for (a) local and (b) global responses respectively for *Cyaniris semiargus*, a species best adapted to local climatic anomalies in temperature during the flight period of the previous year (t-1) of their adult stage. Colors indicate spatial scale (blue, local; orange, global), circles indicate raw data. R^2 values are provided.

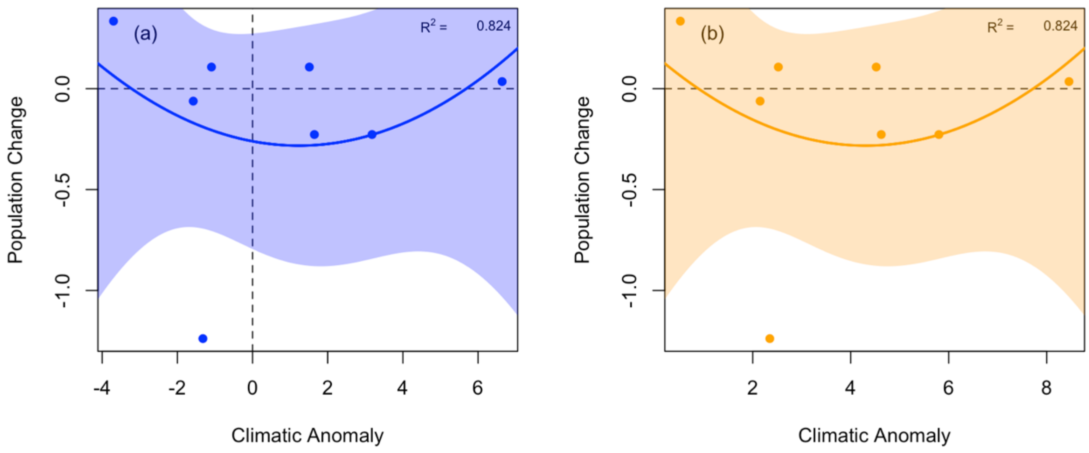

Supplementary Figure 27. Population change in relation to local and global climatic anomalies for (a) local and (b) global responses respectively for *Erebia aethiops*, a species best adapted to global climatic anomalies in precipitation during the overwintering of the year (t) of their adult stage. Colors indicate spatial scale (blue, local; orange, global), circles indicate raw data. R^2 values are provided.

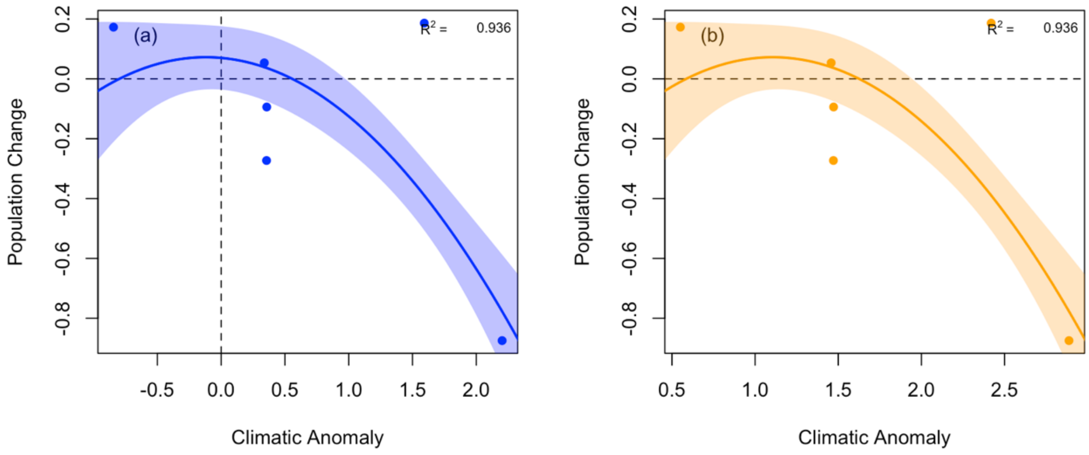

Supplementary Figure 28. Population change in relation to local and global climatic anomalies for (a) local and (b) global responses respectively for *Erebia euryale*, a species best adapted to local climatic anomalies in precipitation during the overwintering of the previous year (t-1) of their adult stage. Colors indicate spatial scale (blue, local; orange, global), circles indicate raw data. R^2 values are provided.

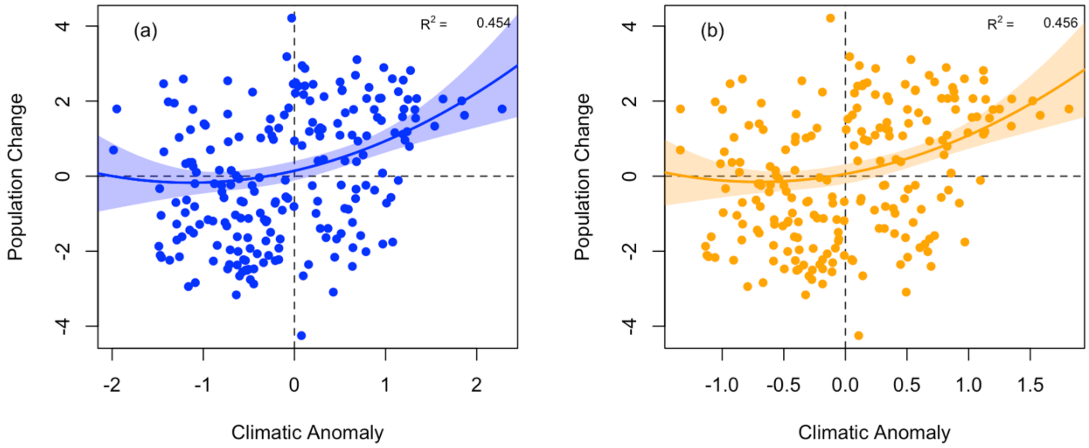

Supplementary Figure 29. Population change in relation to local and global climatic anomalies for (a) local and (b) global responses respectively for *Erebia ligea*, a species best adapted to global climatic anomalies in precipitation during the pre-flight period of the previous year (t-1) of their adult stage. Colors indicate spatial scale (blue, local; orange, global), circles indicate raw data. R^2 values are provided.

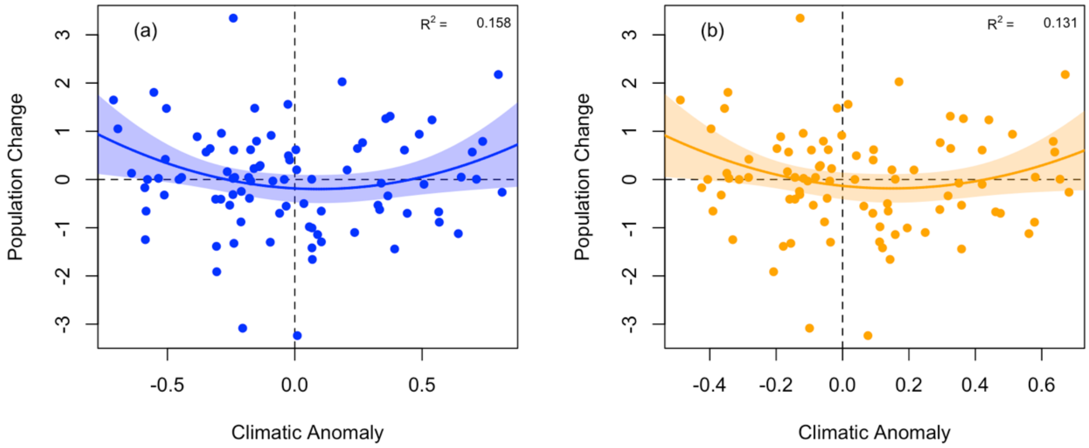

Supplementary Figure 30. Population change in relation to local and global climatic anomalies for (a) local and (b) global responses respectively for Eumedonia eumedon, a species best adapted to local climatic anomalies in precipitation during the flight period of the year (t) of their adult stage. Colors indicate spatial scale (blue, local; orange, global), circles indicate raw data. R^2 values are provided.

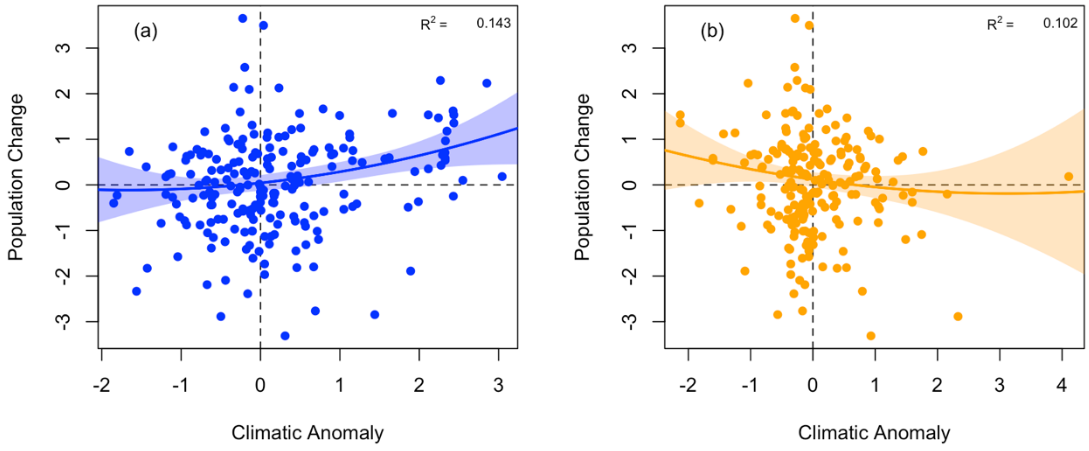

Supplementary Figure 31. Population change in relation to local and global climatic anomalies for (a) local and (b) global responses respectively for *Euphydryas aurinia*, a species best adapted to local climatic anomalies in aridity during the pre-flight period of the previous year (t-1) of their adult stage. Colors indicate spatial scale (blue, local; orange, global), circles indicate raw data. R^2 values are provided.

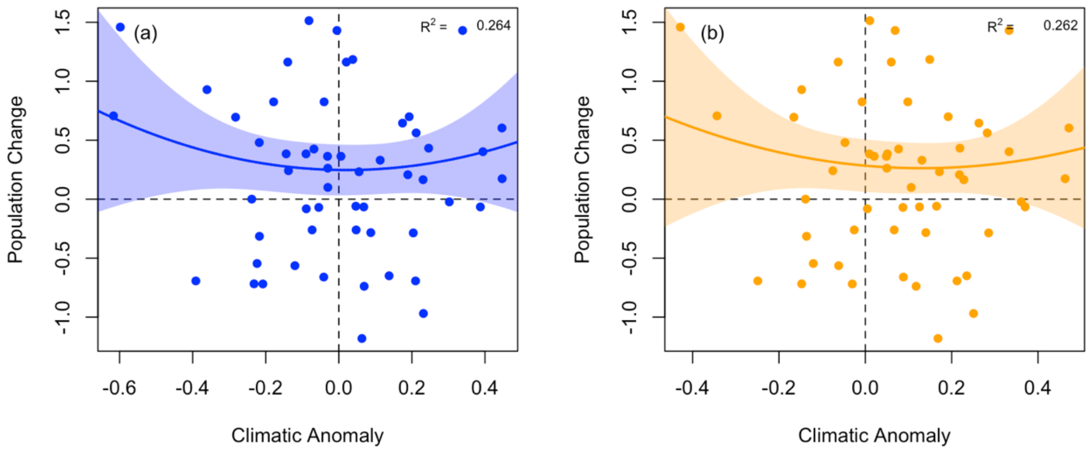

Supplementary Figure 32. Population change in relation to local and global climatic anomalies for (a) local and (b) global responses respectively for *Euphydryas maturna*, a species best adapted to local climatic anomalies in precipitation during the flight period of the year (t) of their adult stage. Colors indicate spatial scale (blue, local; orange, global), circles indicate raw data. R^2 values are provided.

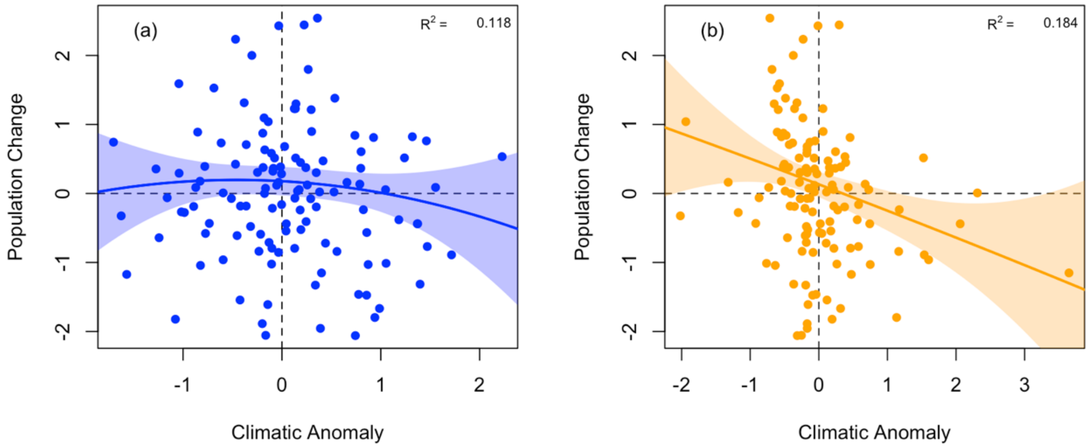

Supplementary Figure 33. Population change in relation to local and global climatic anomalies for (a) local and (b) global responses respectively for *Glaucopsyche alexis*, a species best adapted to global climatic anomalies in aridity during the pre-flight period of the previous year (t-1) of their adult stage. Colors indicate spatial scale (blue, local; orange, global), circles indicate raw data. R^2 values are provided.

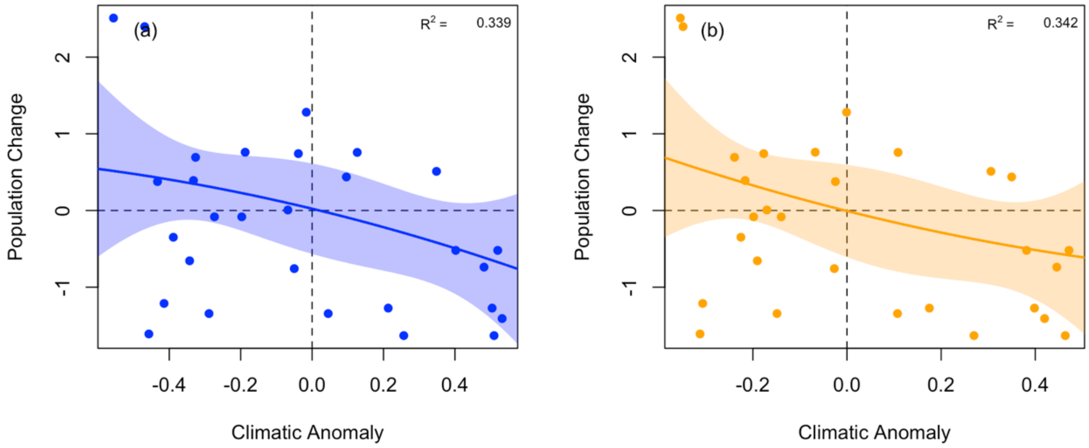

Supplementary Figure 34. Population change in relation to local and global climatic anomalies for (a) local and (b) global responses respectively for *Hamearis lucina*, a species best adapted to global climatic anomalies in precipitation during the flight period of the year (t) of their adult stage. Colors indicate spatial scale (blue, local; orange, global), circles indicate raw data. R^2 values are provided.

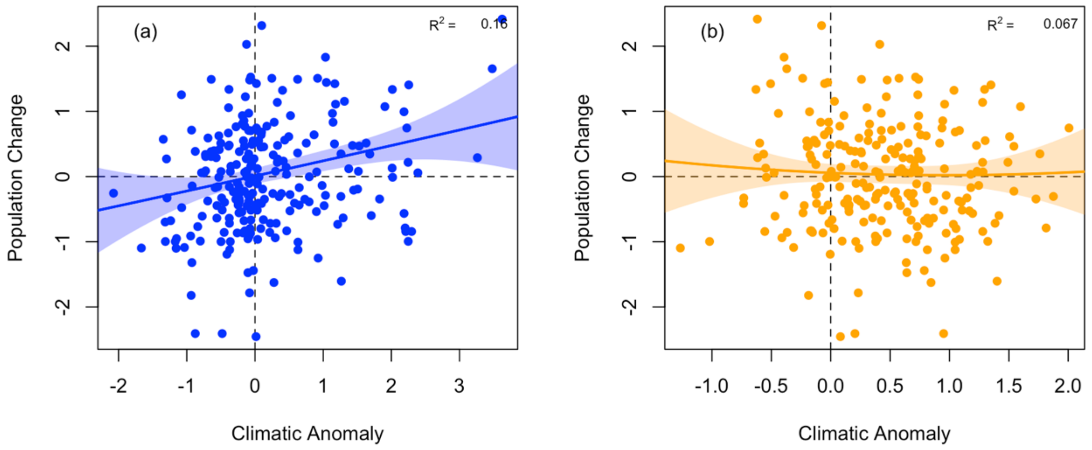

Supplementary Figure 35. Population change in relation to local and global climatic anomalies for (a) local and (b) global responses respectively for *Hipparchia fidia*, a species best adapted to local climatic anomalies in aridity during the overwintering of the year (t) of their adult stage. Colors indicate spatial scale (blue, local; orange, global), circles indicate raw data. R^2 values are provided.

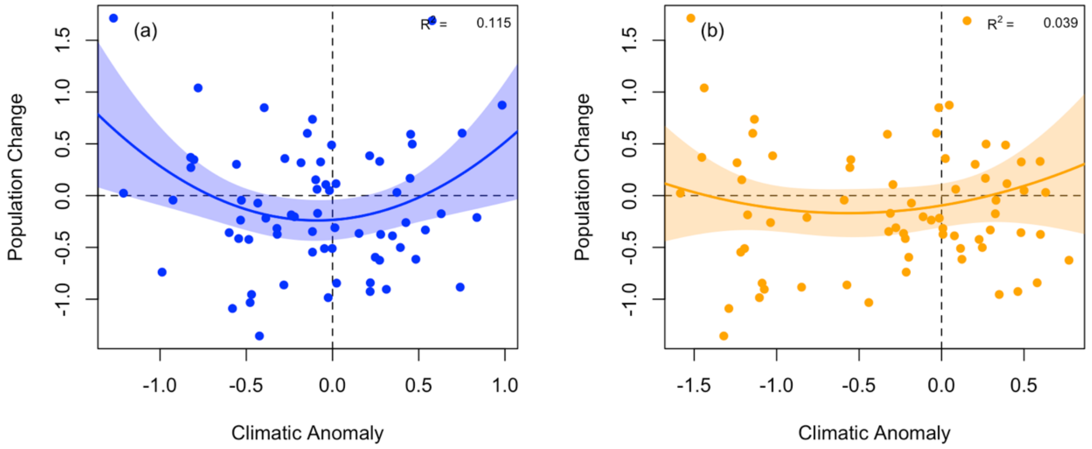

Supplementary Figure 36. Population change in relation to local and global climatic anomalies for (a) local and (b) global responses respectively for *Hipparchia hermione*, a species best adapted to local climatic anomalies in temperature during the pre-flight period of the previous year (t-1) of their adult stage. Colors indicate spatial scale (blue, local; orange, global), circles indicate raw data. R^2 values are provided.

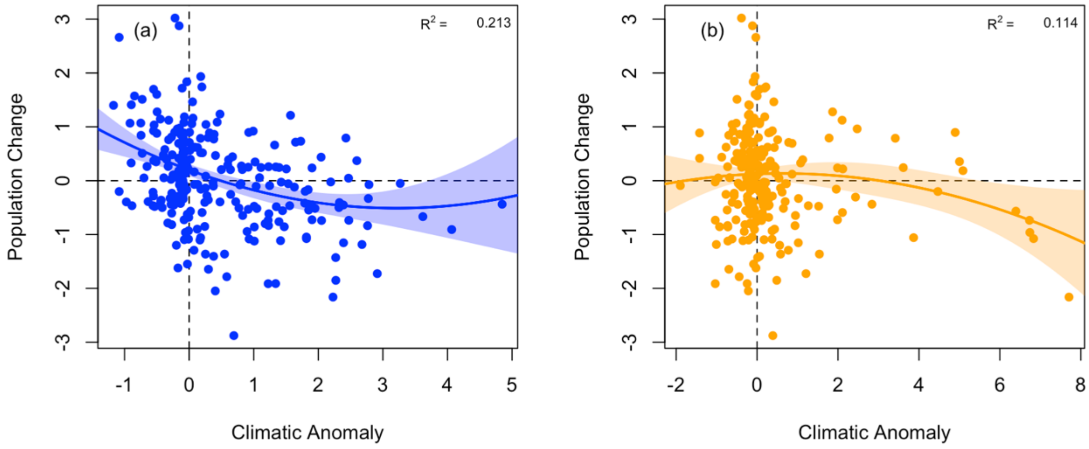

Supplementary Figure 37. Population change in relation to local and global climatic anomalies for (a) local and (b) global responses respectively for *Hipparchia statilinus*, a species best adapted to local climatic anomalies in aridity during the pre-flight period of the previous year (t-1) of their adult stage. Colors indicate spatial scale (blue, local; orange, global), circles indicate raw data. R^2 values are provided.

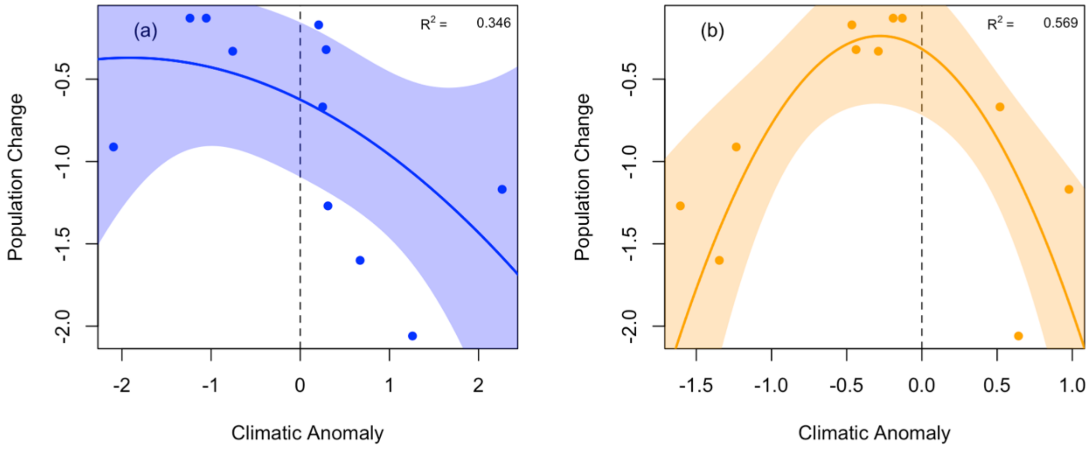

Supplementary Figure 38. Population change in relation to local and global climatic anomalies for (a) local and (b) global responses respectively for *Laeosopis roboris*, a species best adapted to global climatic anomalies in temperature during the flight period of the year (t) of their adult stage. Colors indicate spatial scale (blue, local; orange, global), circles indicate raw data. R^2 values are provided.

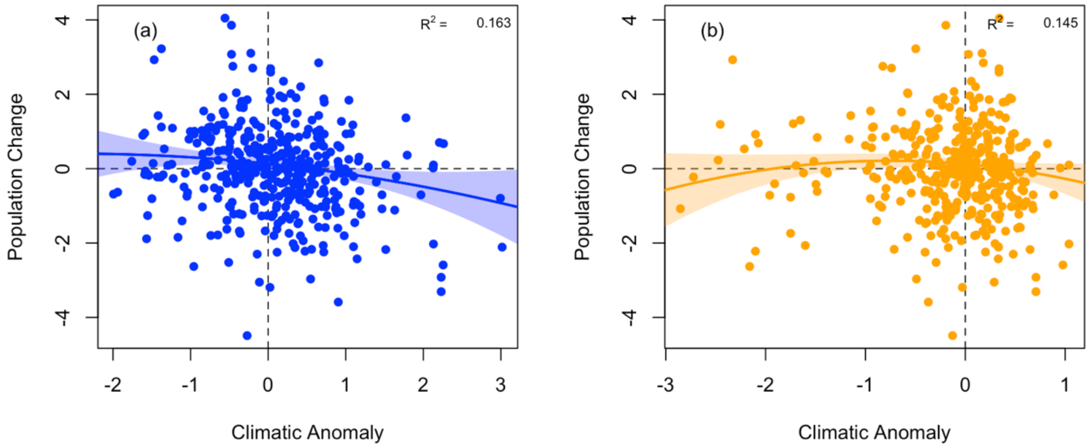

Supplementary Figure 39. Population change in relation to local and global climatic anomalies for (a) local and (b) global responses respectively for *Lampides boeticus*, a species best adapted to local climatic anomalies in temperature during the flight period of the previous year (t-1) of their adult stage. Colors indicate spatial scale (blue, local; orange, global), circles indicate raw data. R^2 values are provided.

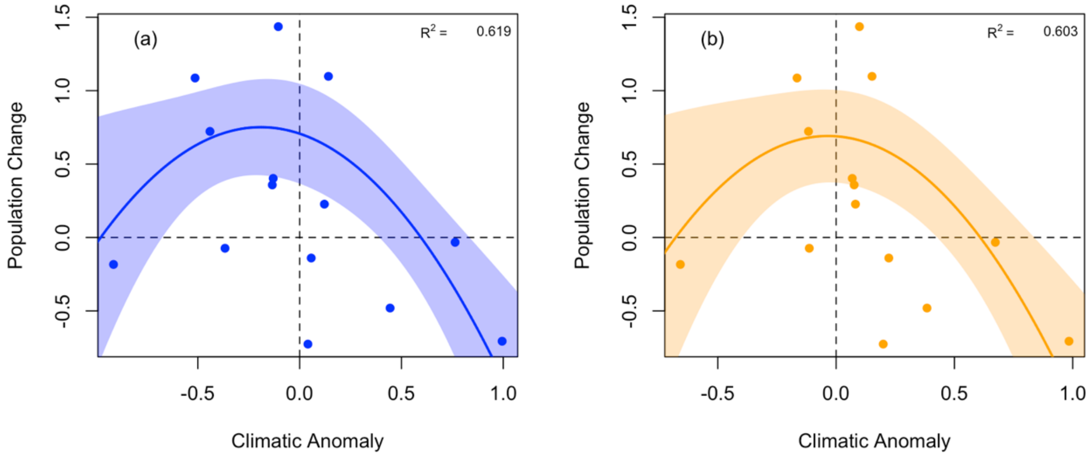

Supplementary Figure 40. Population change in relation to local and global climatic anomalies for (a) local and (b) global responses respectively for *Leptidea juvernica*, a species best adapted to local climatic anomalies in precipitation during the flight period of the previous year (t-1) of their adult stage. Colors indicate spatial scale (blue, local; orange, global), circles indicate raw data. R^2 values are provided.

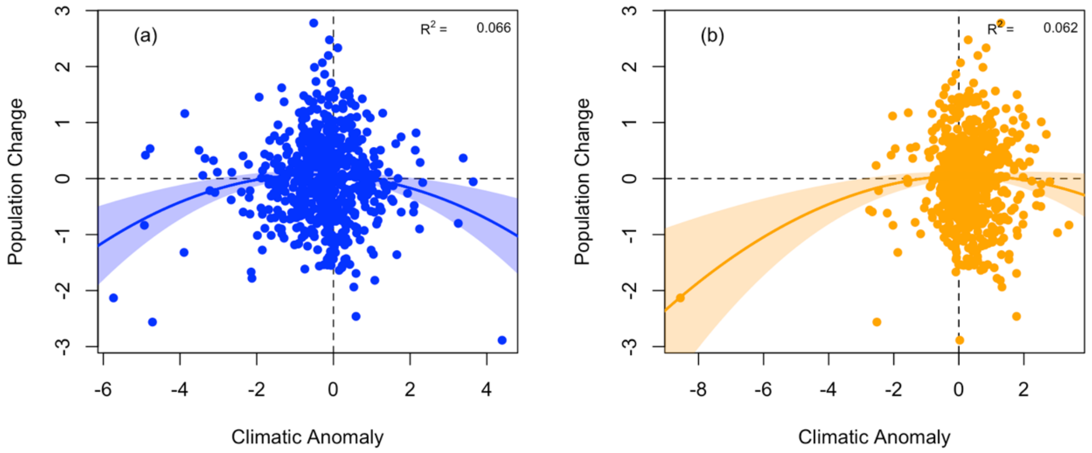

Supplementary Figure 41. Population change in relation to local and global climatic anomalies for (a) local and (b) global responses respectively for *Leptidea sinapis*, a species best adapted to local climatic anomalies in aridity during the post flight period of the previous year (t-1) of their adult stage. Colors indicate spatial scale (blue, local; orange, global), circles indicate raw data. R^2 values are provided.

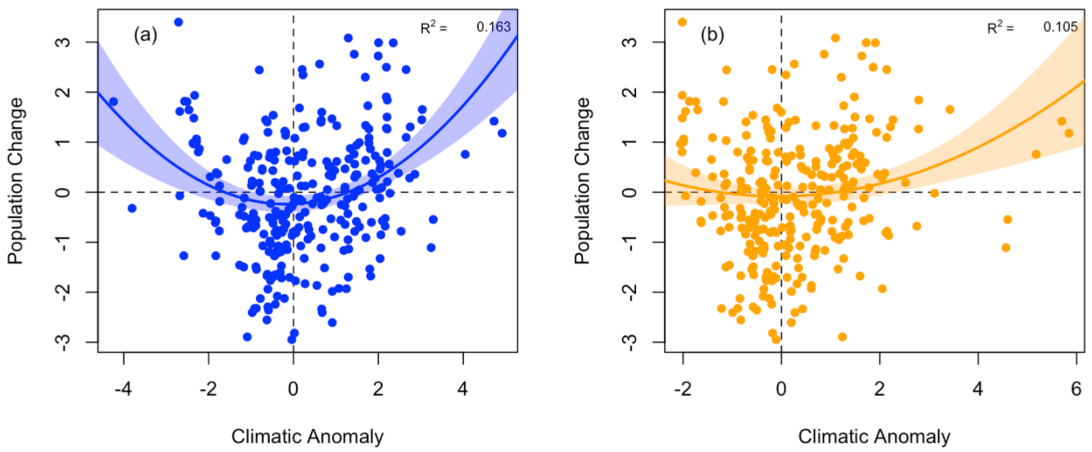

Supplementary Figure 42. Population change in relation to local and global climatic anomalies for (a) local and (b) global responses respectively for *Leptotes pirithous*, a species best adapted to local climatic anomalies in precipitation during the pre-flight period of the previous year (t-1) of their adult stage. Colors indicate spatial scale (blue, local; orange, global), circles indicate raw data. R^2 values are provided.

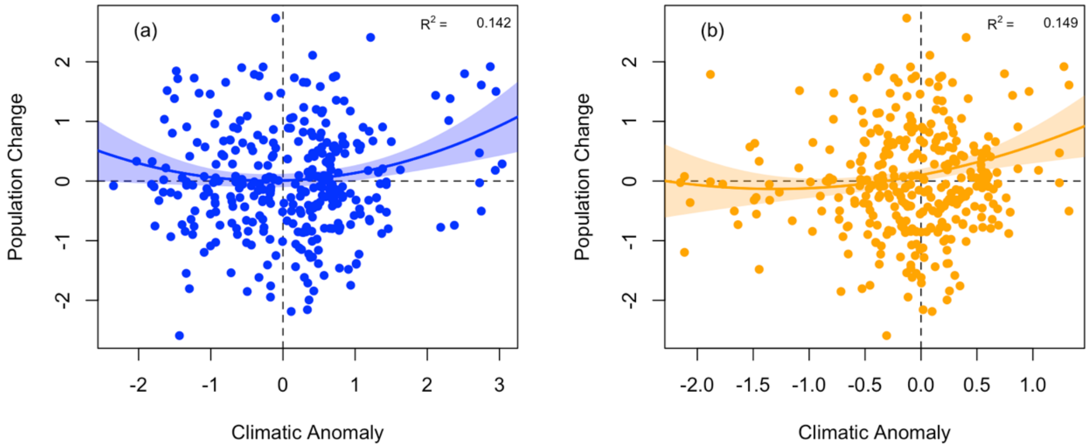

Supplementary Figure 43. Population change in relation to local and global climatic anomalies for (a) local and (b) global responses respectively for *Limenitis reducta*, a species best adapted to global climatic anomalies in temperature during the flight period of the previous year (t-1) of their adult stage. Colors indicate spatial scale (blue, local; orange, global), circles indicate raw data. R^2 values are provided.

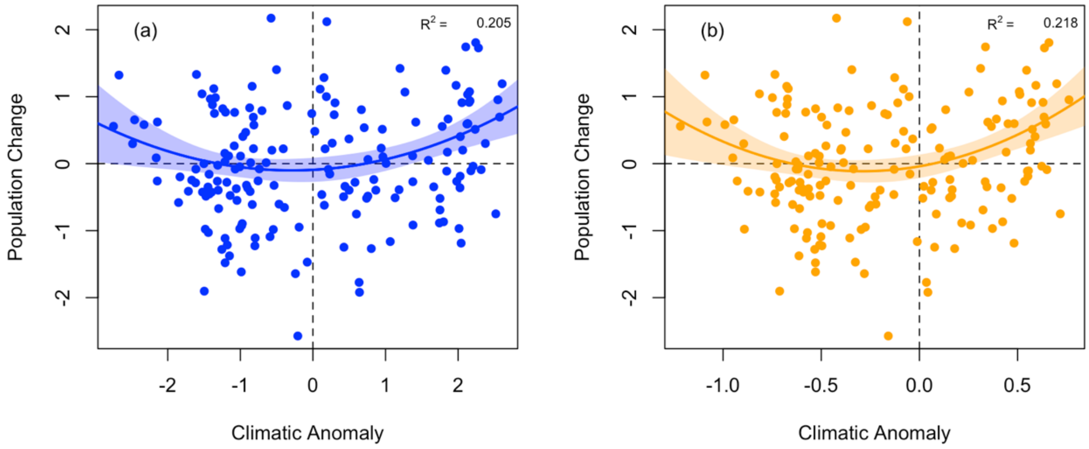

Supplementary Figure 44. Population change in relation to local and global climatic anomalies for (a) local and (b) global responses respectively for *Lycaena hippothoe*, a species best adapted to global climatic anomalies in temperature during the flight period of the previous year (t-1) of their adult stage. Colors indicate spatial scale (blue, local; orange, global), circles indicate raw data. R^2 values are provided.

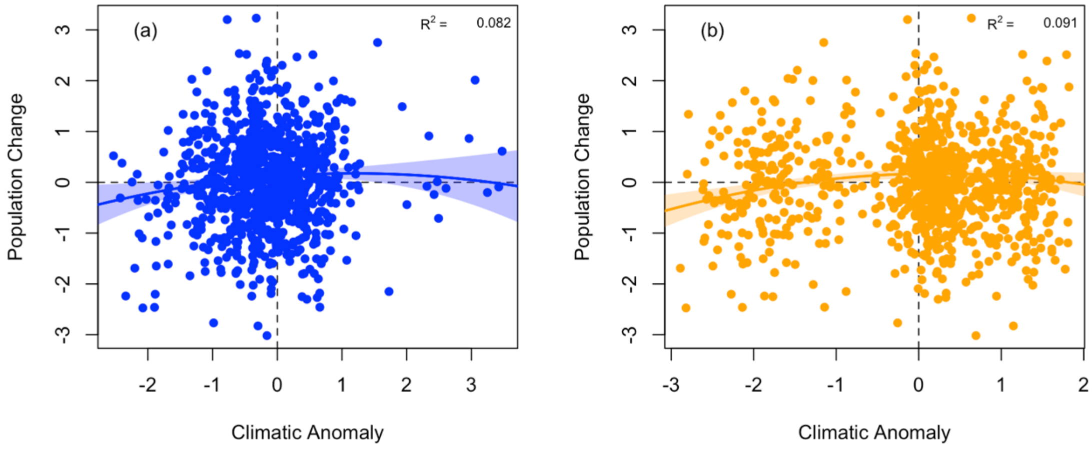

Supplementary Figure 45. Population change in relation to local and global climatic anomalies for (a) local and (b) global responses respectively for *Lycaena phlaeas*, a species best adapted to global climatic anomalies in temperature during the pre-flight period of the previous year (t-1) of their adult stage. Colors indicate spatial scale (blue, local; orange, global), circles indicate raw data. R^2 values are provided.

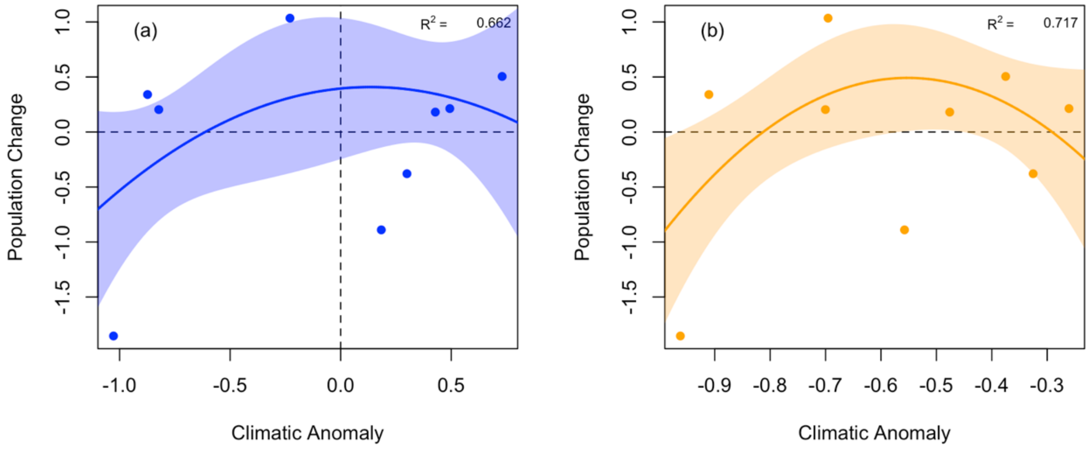

Supplementary Figure 46. Population change in relation to local and global climatic anomalies for (a) local and (b) global responses respectively for *Lycaena tityrus*, a species best adapted to global climatic anomalies in temperature during the post flight period of the previous year (t-1) of their adult stage. Colors indicate spatial scale (blue, local; orange, global), circles indicate raw data. R2 values are provided.

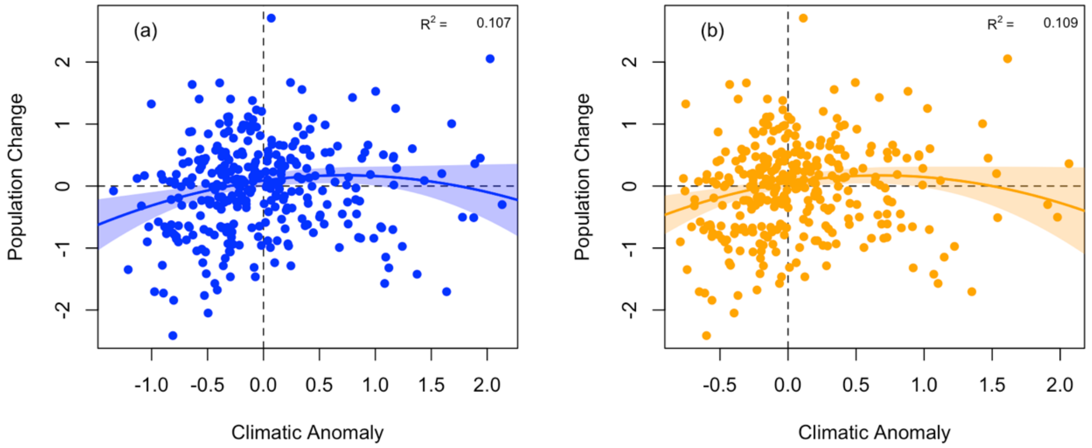

Supplementary Figure 47. Population change in relation to local and global climatic anomalies

for (a) local and (b) global responses respectively for *Lycaena virgaureae*, a species best adapted to global climatic anomalies in precipitation during the flight period of the year (t) of their adult stage. Colors indicate spatial scale (blue, local; orange, global), circles indicate raw data. R2 values are provided.

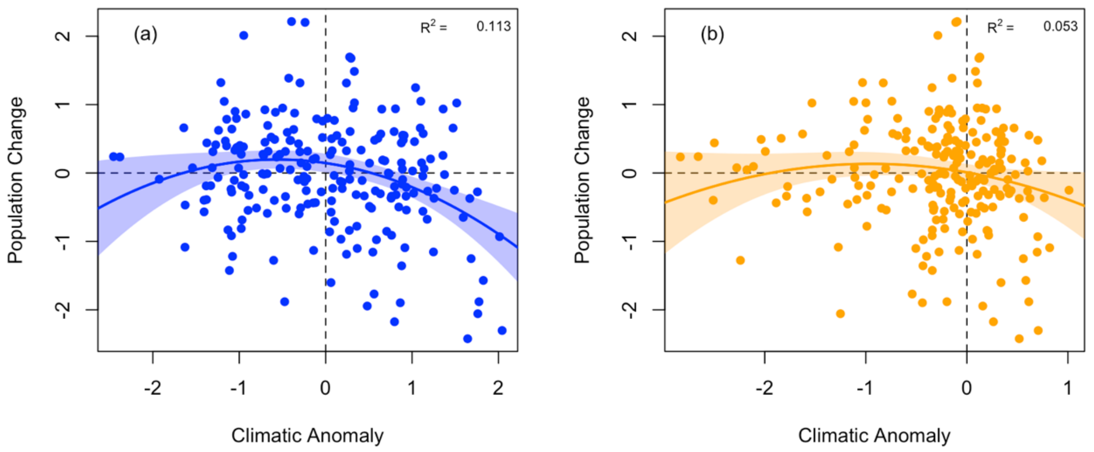

Supplementary Figure 48. Population change in relation to local and global climatic anomalies for (a) local and (b) global responses respectively for *Lysandra coridon*, a species best adapted to local climatic anomalies in temperature during the post flight period of the previous year (t-1) of their adult stage. Colors indicate spatial scale (blue, local; orange, global), circles indicate raw data. R^2 values are provided.

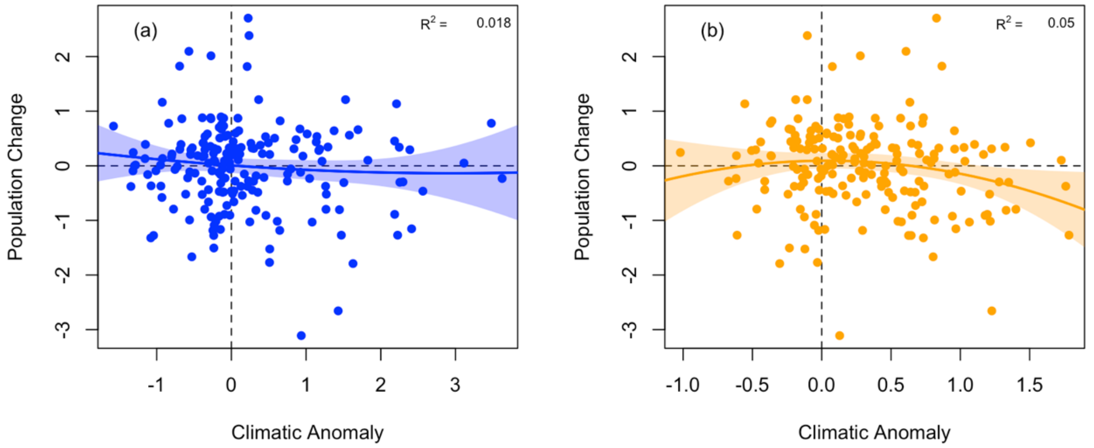

Supplementary Figure 49. Population change in relation to local and global climatic anomalies for (a) local and (b) global responses respectively for *Lysandra hispana*, a species best adapted to global climatic anomalies in aridity during the overwintering of the year (t) of their adult stage. Colors indicate spatial scale (blue, local; orange, global), circles indicate raw data. R^2 values are provided.

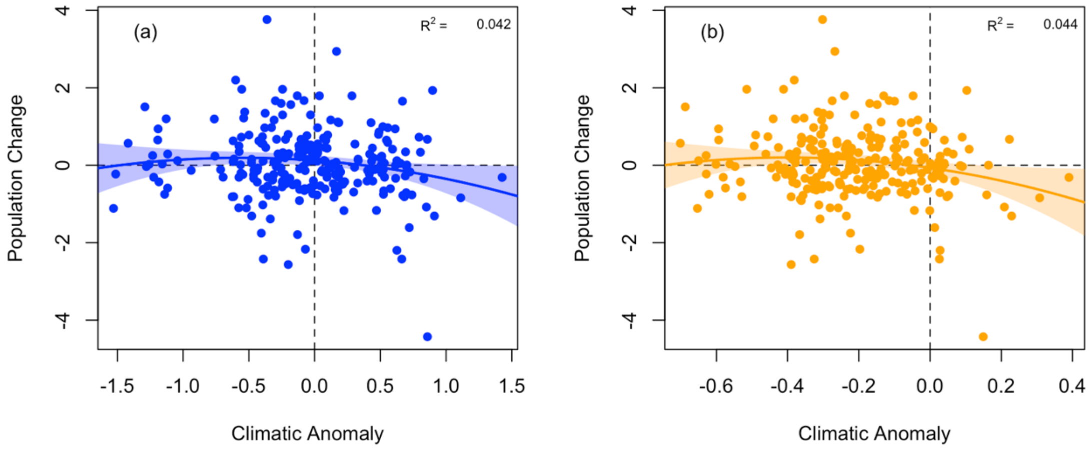

Supplementary Figure 50. Population change in relation to local and global climatic anomalies for (a) local and (b) global responses respectively for *Melanargia galathea*, a species best adapted to global climatic anomalies in temperature during the pre-flight period of the year (t) of their adult stage. Colors indicate spatial scale (blue, local; orange, global), circles indicate raw data. R^2 values are provided.

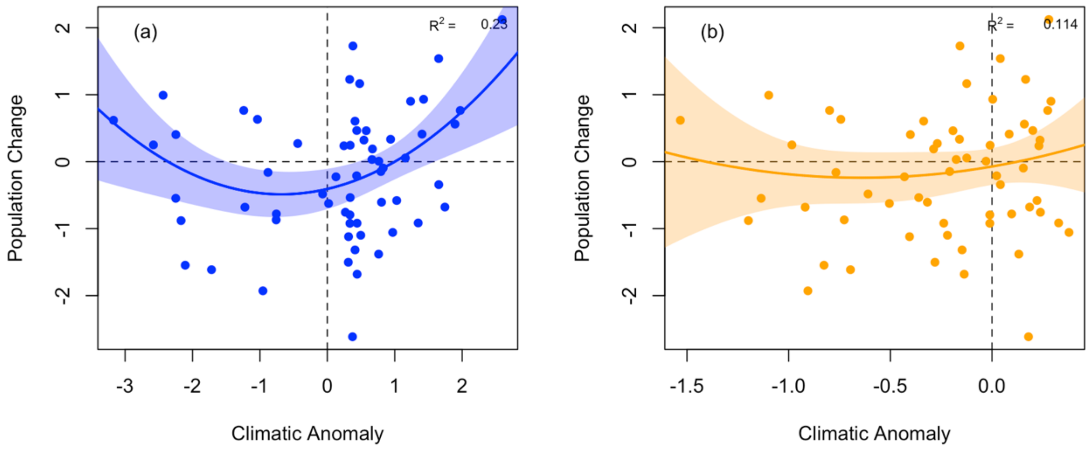

Supplementary Figure 51. Population change in relation to local and global climatic anomalies for (a) local and (b) global responses respectively for *Melanargia occitanica*, a species best adapted to local climatic anomalies in temperature during the flight period of the previous year (t-1) of their adult stage. Colors indicate spatial scale (blue, local; orange, global), circles indicate raw data. R^2 values are provided.

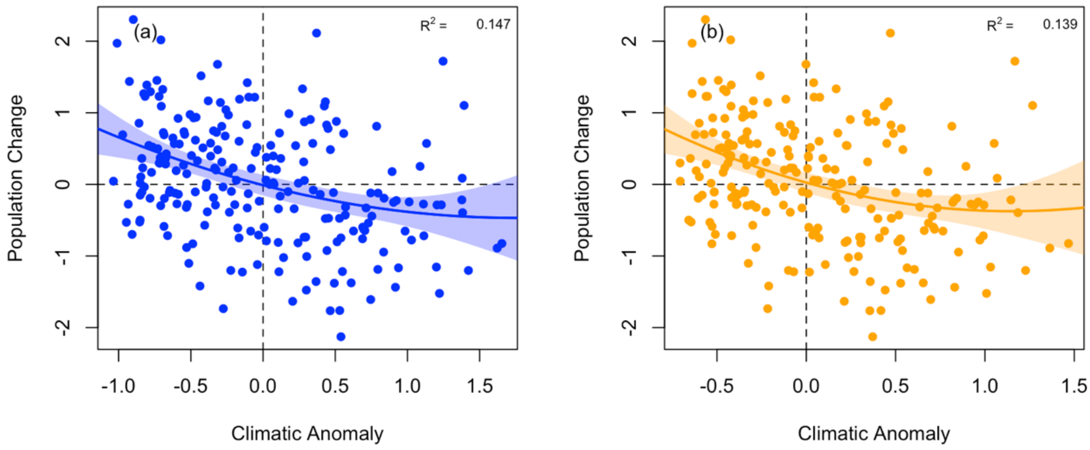

Supplementary Figure 52. Population change in relation to local and global climatic anomalies for (a) local and (b) global responses respectively for *Melitaea athalia*, a species best adapted to local climatic anomalies in temperature during the post flight period of the previous year (t-1) of their adult stage. Colors indicate spatial scale (blue, local; orange, global), circles indicate raw data. R^2 values are provided.

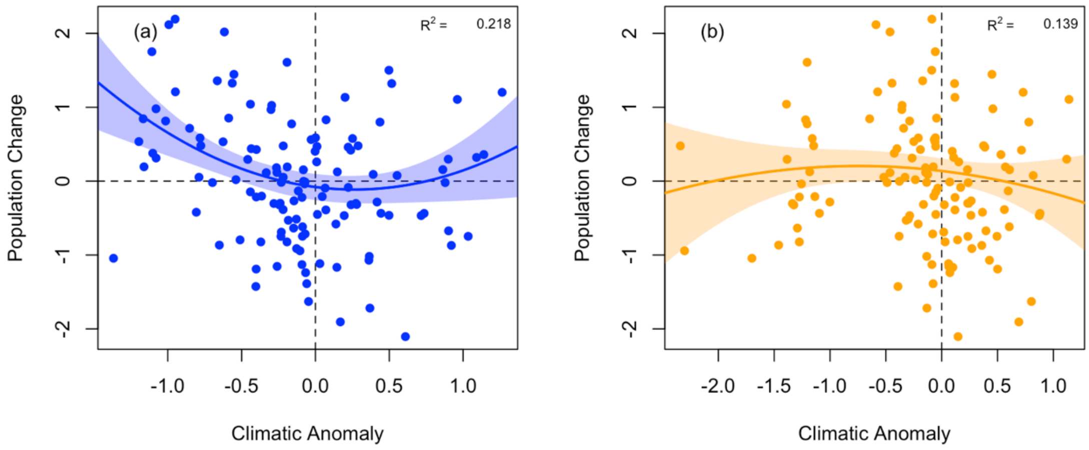

Supplementary Figure 53. Population change in relation to local and global climatic anomalies for (a) local and (b) global responses respectively for *Melitaea cinxia*, a species best adapted to local climatic anomalies in precipitation during the flight period of the previous year (t-1) of their adult stage. Colors indicate spatial scale (blue, local; orange, global), circles indicate raw data. R^2 values are provided.

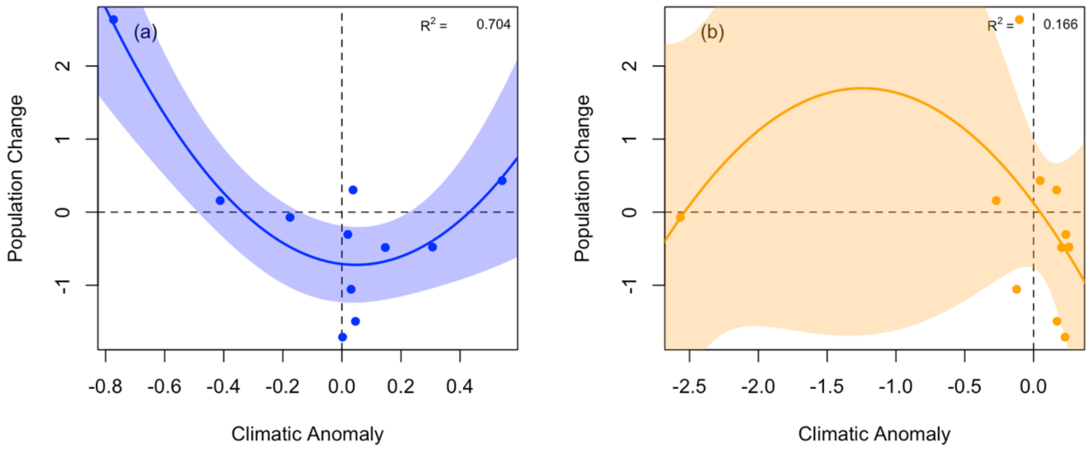

Supplementary Figure 54. Population change in relation to local and global climatic anomalies for (a) local and (b) global responses respectively for *Melitaea parthenoides*, a species best adapted to local climatic anomalies in temperature during the pre-flight period of the previous year (t-1) of their adult stage. Colors indicate spatial scale (blue, local; orange, global), circles indicate raw data. R^2 values are provided.

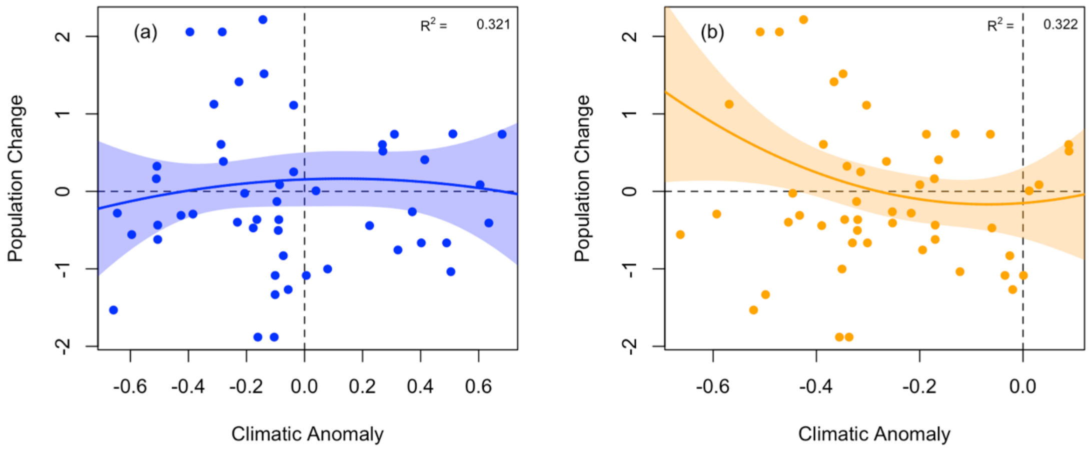

Supplementary Figure 55. Population change in relation to local and global climatic anomalies for (a) local and (b) global responses respectively for *Muschampia proto*, a species best adapted to global climatic anomalies in temperature during the pre-flight period of the year (t) of their adult stage. Colors indicate spatial scale (blue, local; orange, global), circles indicate raw data. R^2 values are provided.

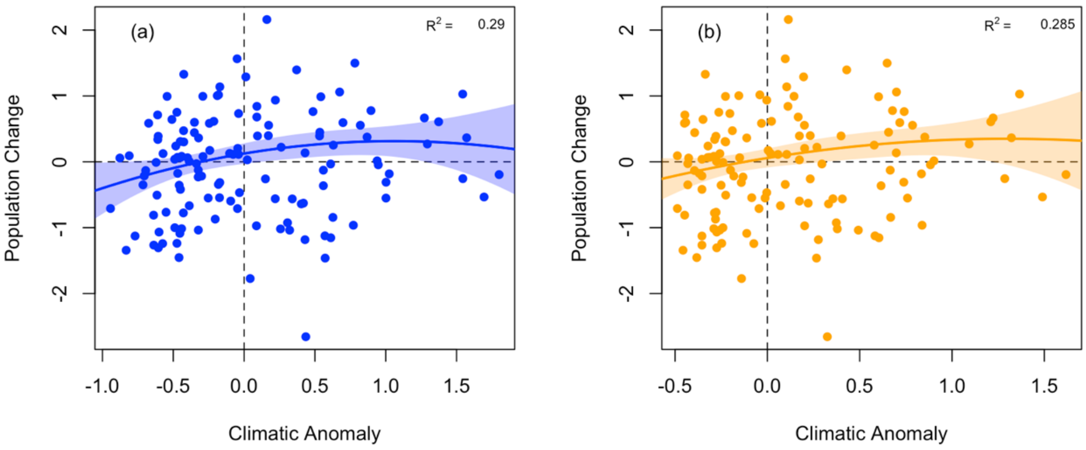

Supplementary Figure 56. Population change in relation to local and global climatic anomalies for (a) local and (b) global responses respectively for *Nymphalis polychloros*, a species best adapted to local climatic anomalies in precipitation during the pre-flight period of the previous year (t-1) of their adult stage. Colors indicate spatial scale (blue, local; orange, global), circles indicate raw data. R^2 values are provided.

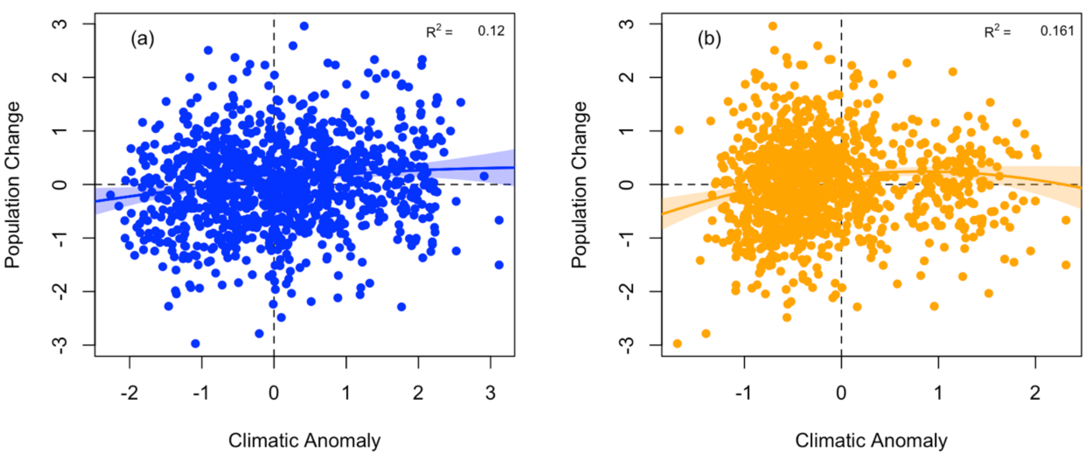

Supplementary Figure 57. Population change in relation to local and global climatic anomalies for (a) local and (b) global responses respectively for *Ochlodes sylvanus*, a species best adapted to global climatic anomalies in temperature during the flight period of the previous year (t-1) of their adult stage. Colors indicate spatial scale (blue, local; orange, global), circles indicate raw data. R^2 values are provided.

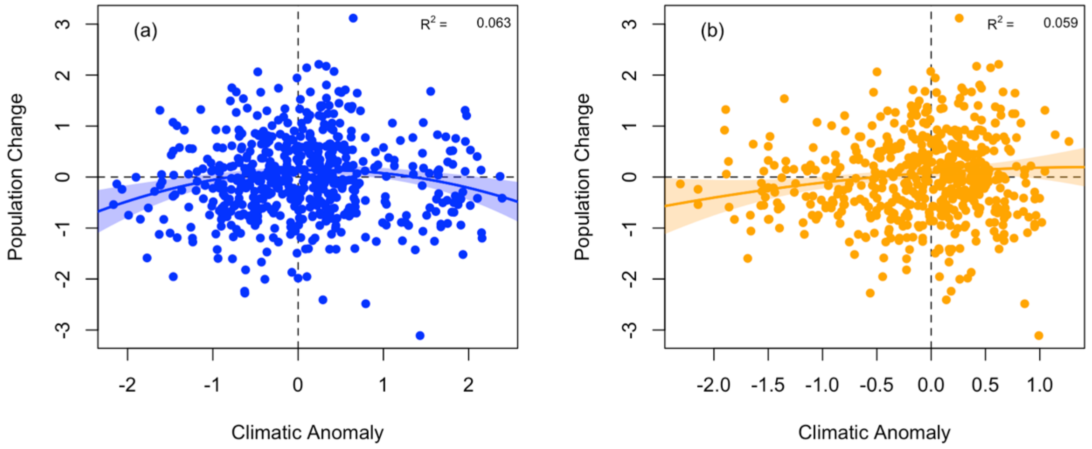

Supplementary Figure 58. Population change in relation to local and global climatic anomalies for (a) local and (b) global responses respectively for *Papilio machaon*, a species best adapted to local climatic anomalies in temperature during the flight period of the year (t) of their adult stage. Colors indicate spatial scale (blue, local; orange, global), circles indicate raw data. R^2 values are provided.

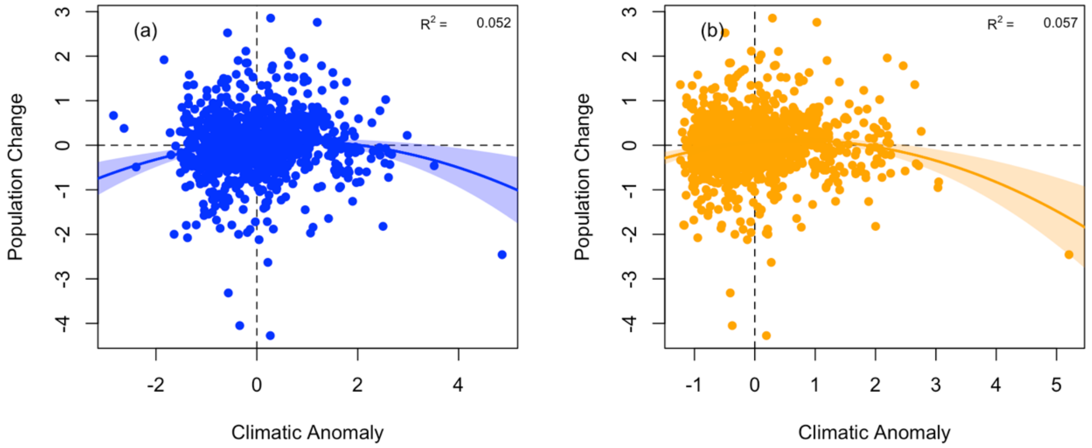

Supplementary Figure 59. Population change in relation to local and global climatic anomalies for (a) local and (b) global responses respectively for *Pararge aegeria*, a species best adapted to global climatic anomalies in precipitation during the post flight period of the previous year (t-1) of their adult stage. Colors indicate spatial scale (blue, local; orange, global), circles indicate raw data. R^2 values are provided.

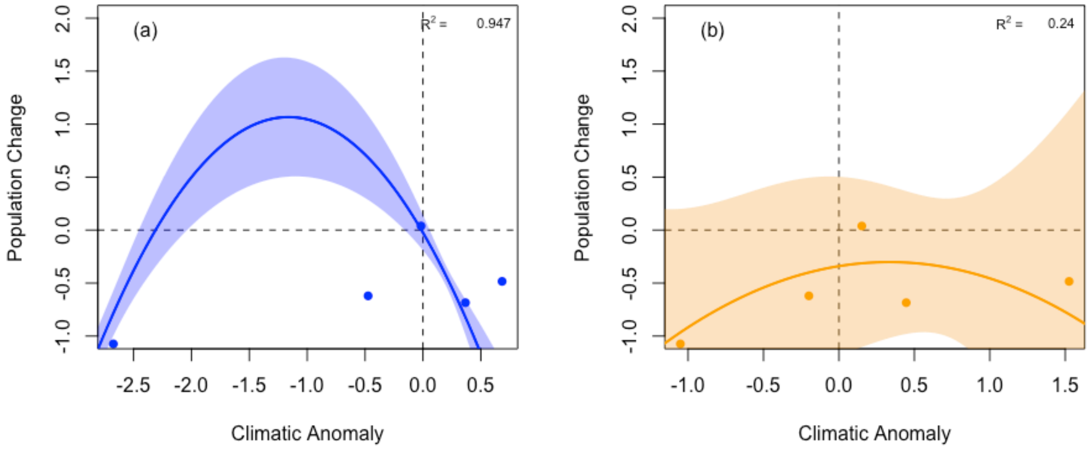

Supplementary Figure 60. Population change in relation to local and global climatic anomalies

for (a) local and (b) global responses respectively for *Parnassius apollo*, a species best adapted to local climatic anomalies in precipitation during the pre-flight period of the previous year (t-1) of their adult stage. Colors indicate spatial scale (blue, local; orange, global), circles indicate raw data. R^2 values are provided.

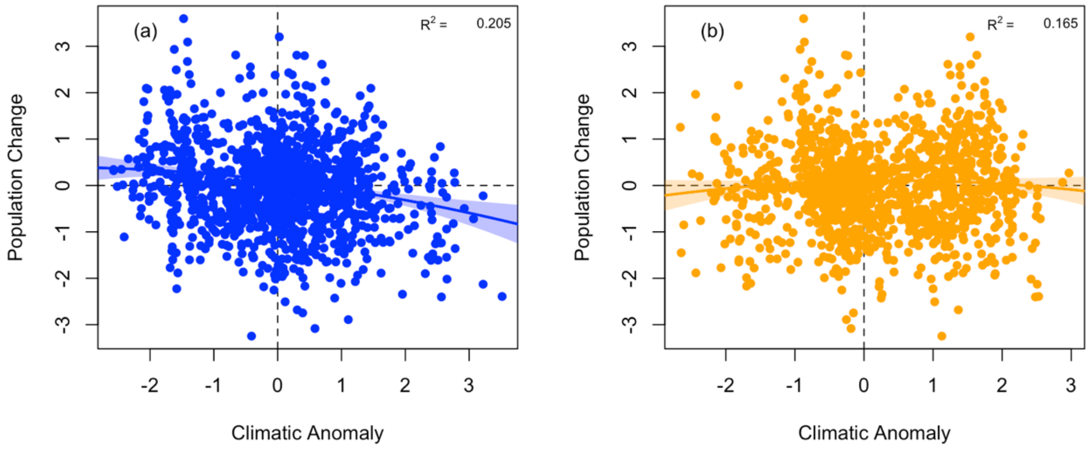

Supplementary Figure 61. Population change in relation to local and global climatic anomalies for (a) local and (b) global responses respectively for *Pieris brassicae*, a species best adapted to local climatic anomalies in temperature during the post flight period of the previous year (t-1) of their adult stage. Colors indicate spatial scale (blue, local; orange, global), circles indicate raw data. R^2 values are provided.

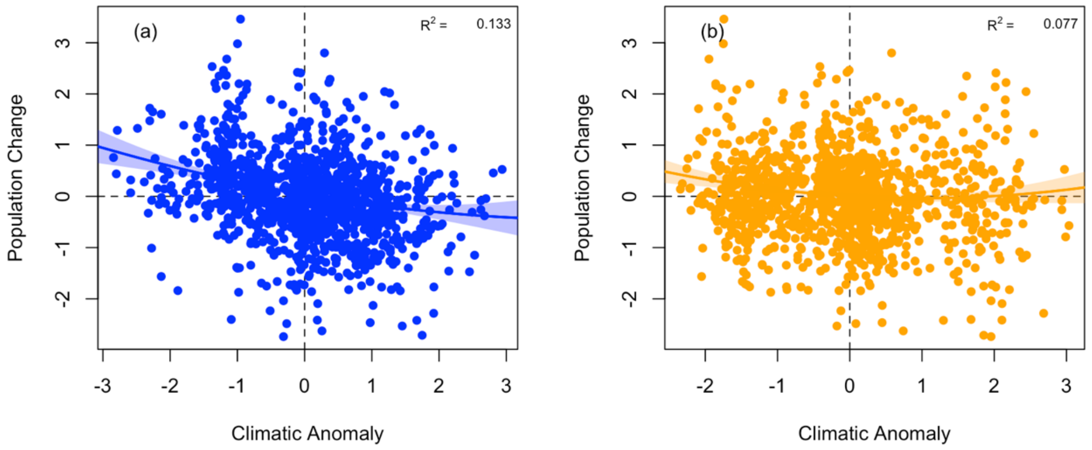

Supplementary Figure 62. Population change in relation to local and global climatic anomalies for (a) local and (b) global responses respectively for *Pieris napi*, a species best adapted to local climatic anomalies in temperature during the post flight period of the previous year (t-1) of their adult stage. Colors indicate spatial scale (blue, local; orange, global), circles indicate raw data. R^2 values are provided.

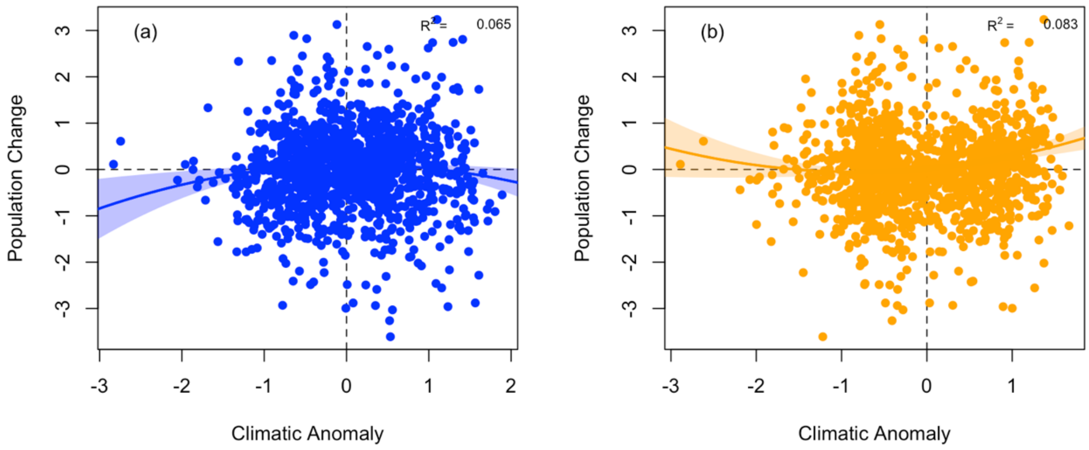

Supplementary Figure 63. Population change in relation to local and global climatic anomalies for (a) local and (b) global responses respectively for *Pieris rapae*, a species best adapted to global climatic anomalies in temperature during the flight period of the previous year (t-1) of their adult stage. Colors indicate spatial scale (blue, local; orange, global), circles indicate raw data. R^2 values are provided.

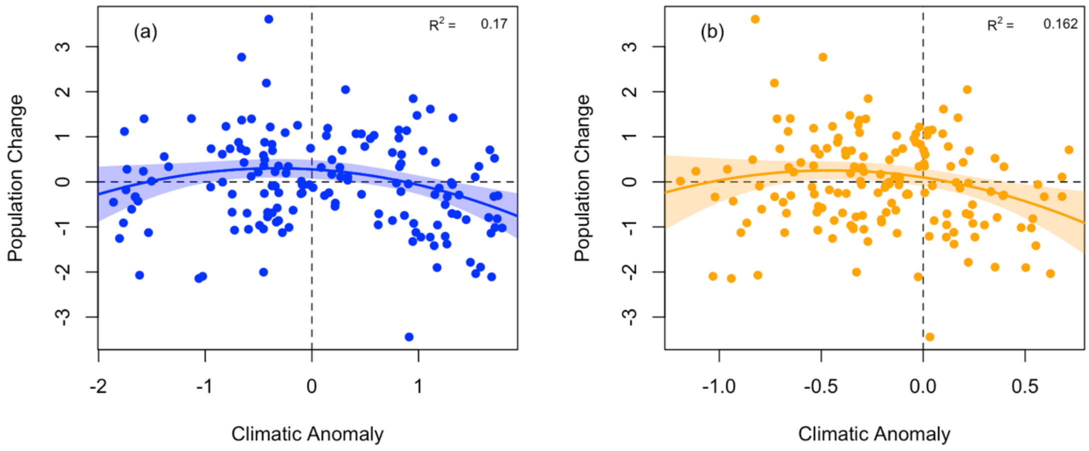

Supplementary Figure 64. Population change in relation to local and global climatic anomalies for (a) local and (b) global responses respectively for *Plebejus idas*, a species best adapted to local climatic anomalies in temperature during the post flight period of the previous year (t-1) of their adult stage. Colors indicate spatial scale (blue, local; orange, global), circles indicate raw data. R^2 values are provided.

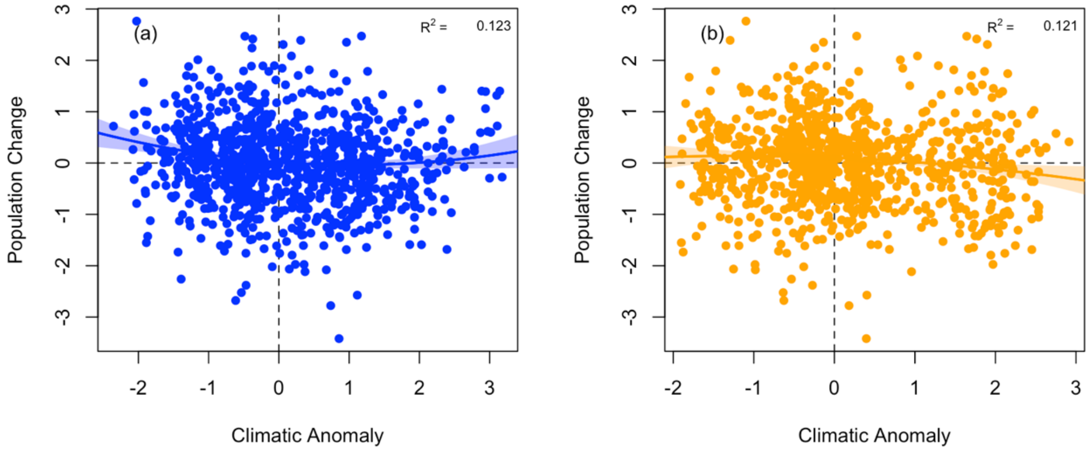

Supplementary Figure 65. Population change in relation to local and global climatic anomalies for (a) local and (b) global responses respectively for *Polygonia c-album*, a species best adapted to local climatic anomalies in temperature during the post flight period of the previous year (t-1) of their adult stage. Colors indicate spatial scale (blue, local; orange, global), circles indicate raw data. R^2 values are provided.

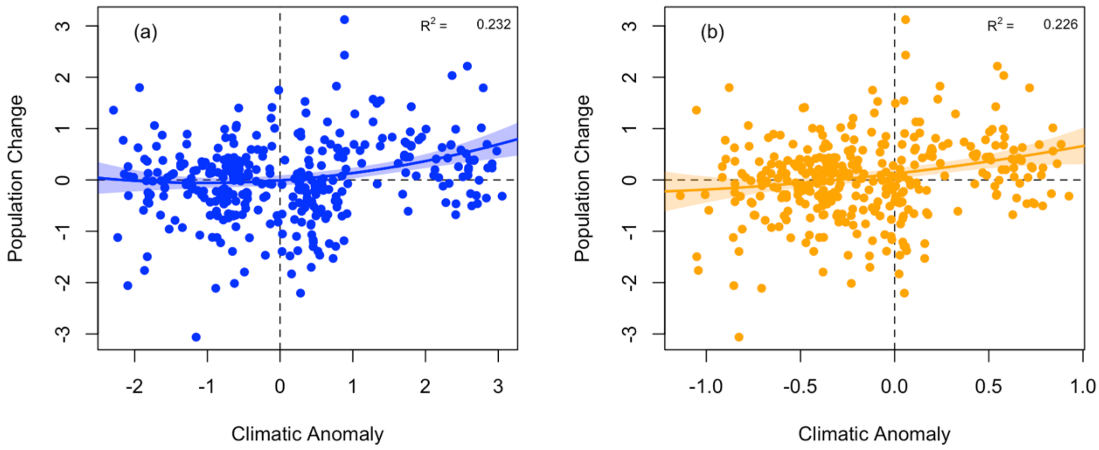

Supplementary Figure 66. Population change in relation to local and global climatic anomalies for (a) local and (b) global responses respectively for *Polyommatus amandus*, a species best adapted to local climatic anomalies in temperature during the flight period of the previous year (t-1) of their adult stage. Colors indicate spatial scale (blue, local; orange, global), circles indicate raw data. R^2 values are provided.

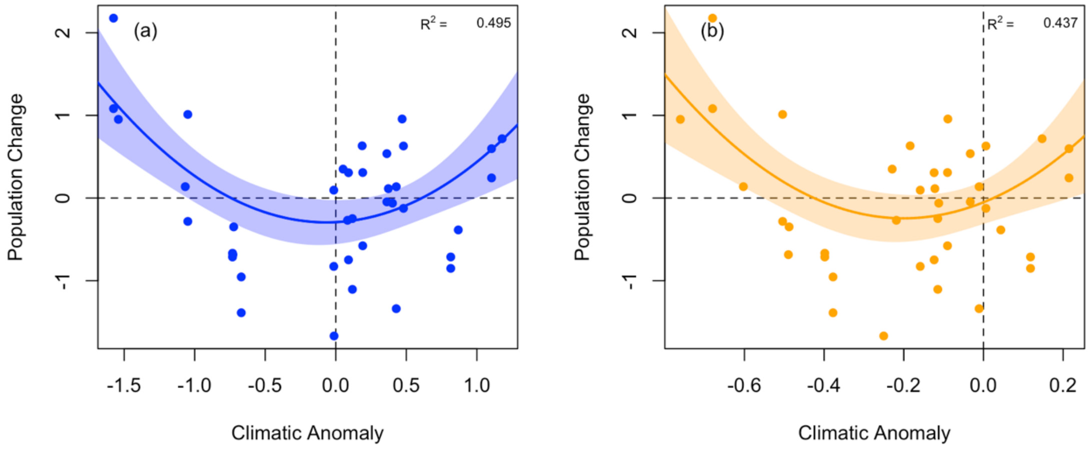

Supplementary Figure 67. Population change in relation to local and global climatic anomalies for (a) local and (b) global responses respectively for *Polyommatus celina*, a species best adapted to local climatic anomalies in temperature during the pre-flight period of the previous year (t-1) of their adult stage. Colors indicate spatial scale (blue, local; orange, global), circles indicate raw data. R^2 values are provided.

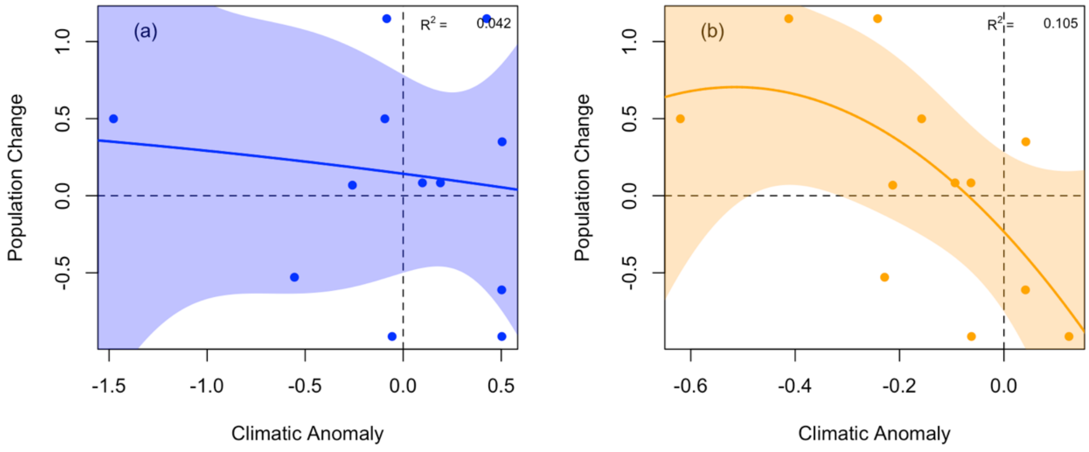

Supplementary Figure 68. Population change in relation to local and global climatic anomalies for (a) local and (b) global responses respectively for *Polyommatus fulgens*, a species best adapted to global climatic anomalies in temperature during the flight period of the year (t) of their adult stage. Colors indicate spatial scale (blue, local; orange, global), circles indicate raw data. R^2 values are provided.

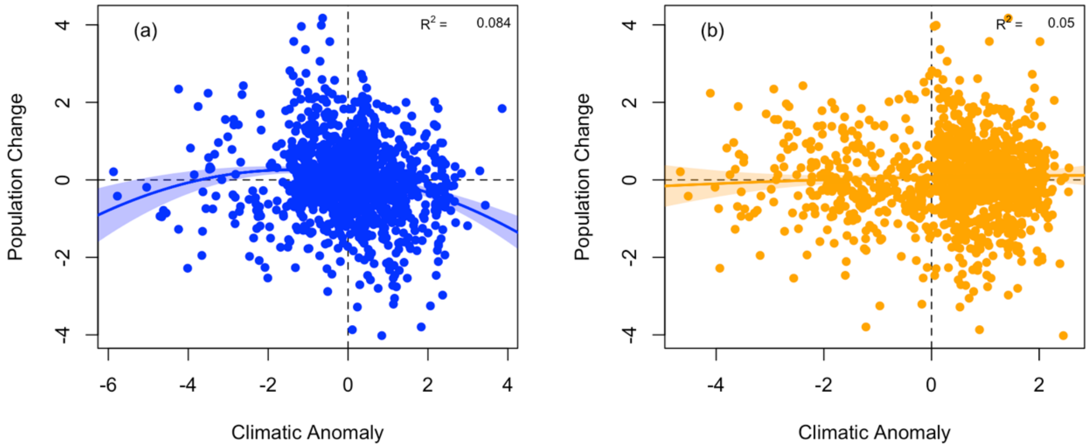

Supplementary Figure 69. Population change in relation to local and global climatic anomalies for (a) local and (b) global responses respectively for *Polyommatus icarus*, a species best adapted to local climatic anomalies in temperature during the overwintering of the previous year (t-1) of their adult stage. Colors indicate spatial scale (blue, local; orange, global), circles indicate raw data. R^2 values are provided.

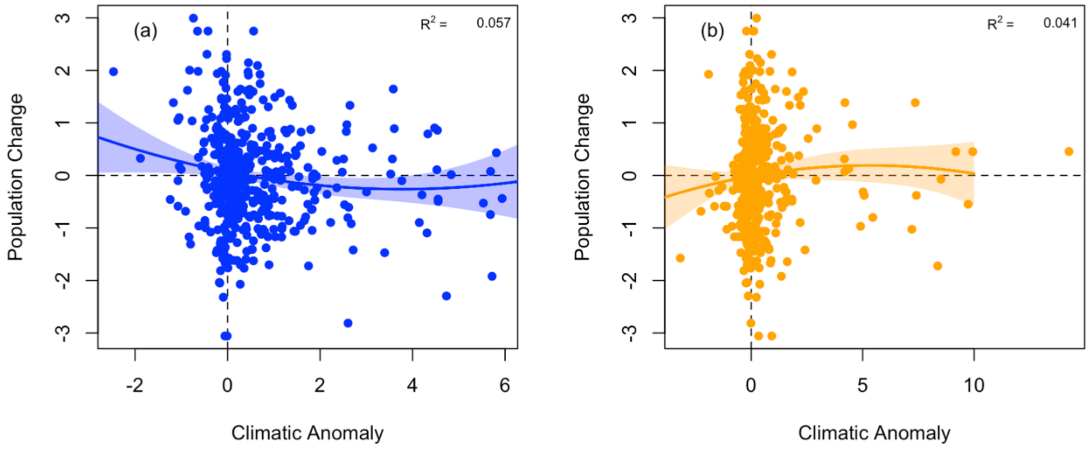

Supplementary Figure 70. Population change in relation to local and global climatic anomalies for (a) local and (b) global responses respectively for *Pontia daplidice*, a species best adapted to local climatic anomalies in aridity during the flight period of the year (t) of their adult stage. Colors indicate spatial scale (blue, local; orange, global), circles indicate raw data. R^2 values are provided.

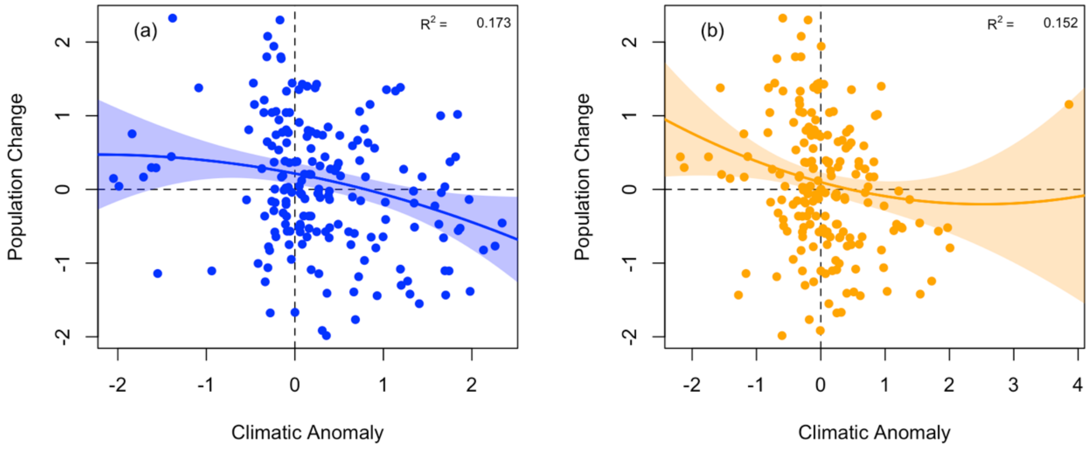

Supplementary Figure 71. Population change in relation to local and global climatic anomalies for (a) local and (b) global responses respectively for *Pseudophilotes panoptes*, a species best adapted to local climatic anomalies in aridity during the pre-flight period of the year (t) of their adult stage. Colors indicate spatial scale (blue, local; orange, global), circles indicate raw data. R^2 values are provided.

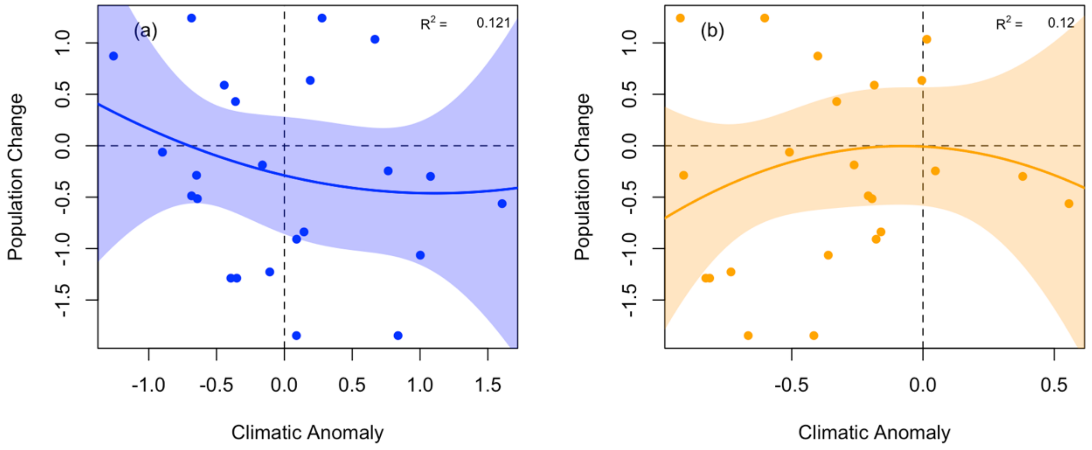

Supplementary Figure 72. Population change in relation to local and global climatic anomalies for (a) local and (b) global responses respectively for *Pyrgus armoricanus*, a species best adapted to local climatic anomalies in temperature during the flight period of the year (t) of their adult stage. Colors indicate spatial scale (blue, local; orange, global), circles indicate raw data. R^2 values are provided.

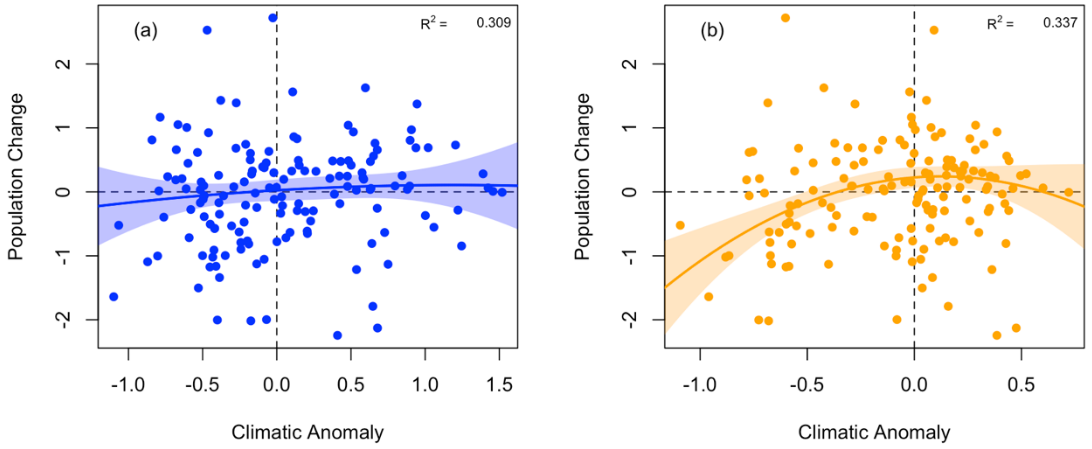

Supplementary Figure 73. Population change in relation to local and global climatic anomalies for (a) local and (b) global responses respectively for *Pyrgus malvae*, a species best adapted to global climatic anomalies in temperature during the post flight period of the previous year (t-1) of their adult stage. Colors indicate spatial scale (blue, local; orange, global), circles indicate raw data. R^2 values are provided.

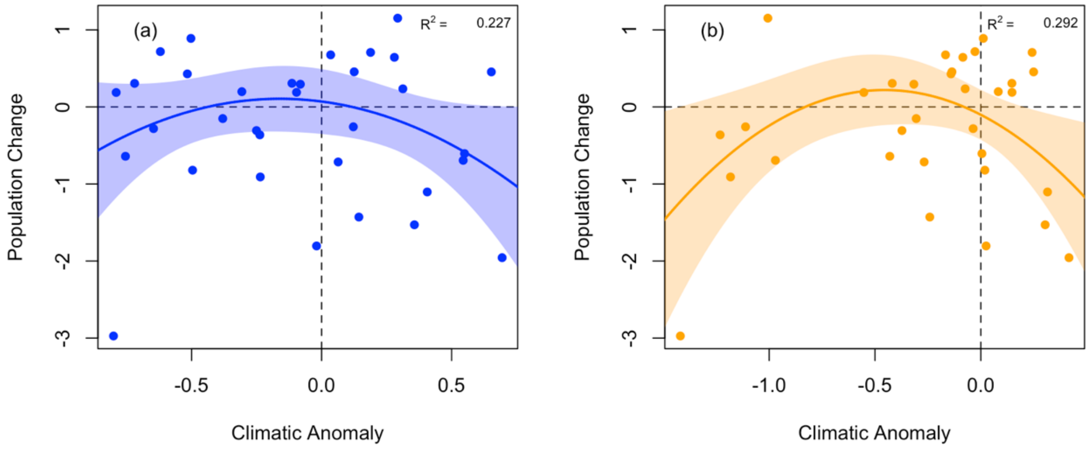

Supplementary Figure 74. Population change in relation to local and global climatic anomalies for (a) local and (b) global responses respectively for *Satyrium acaciae*, a species best adapted to global climatic anomalies in temperature during the pre-flight period of the previous year (t-1) of their adult stage. Colors indicate spatial scale (blue, local; orange, global), circles indicate raw data. R^2 values are provided.

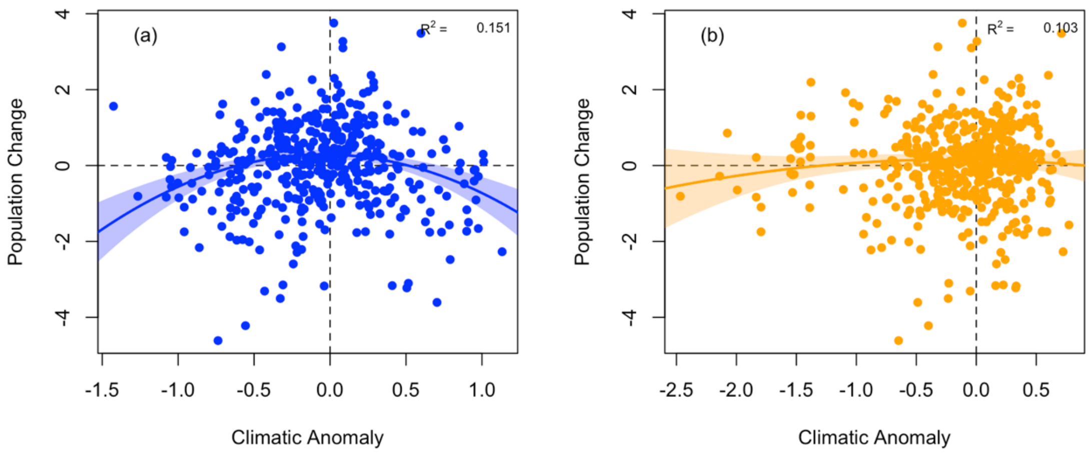

Supplementary Figure 75. Population change in relation to local and global climatic anomalies for (a) local and (b) global responses respectively for *Satyrium esculi*, a species best adapted to local climatic anomalies in temperature during the pre-flight period of the previous year (t-1) of their adult stage. Colors indicate spatial scale (blue, local; orange, global), circles indicate raw data. R^2 values are provided.

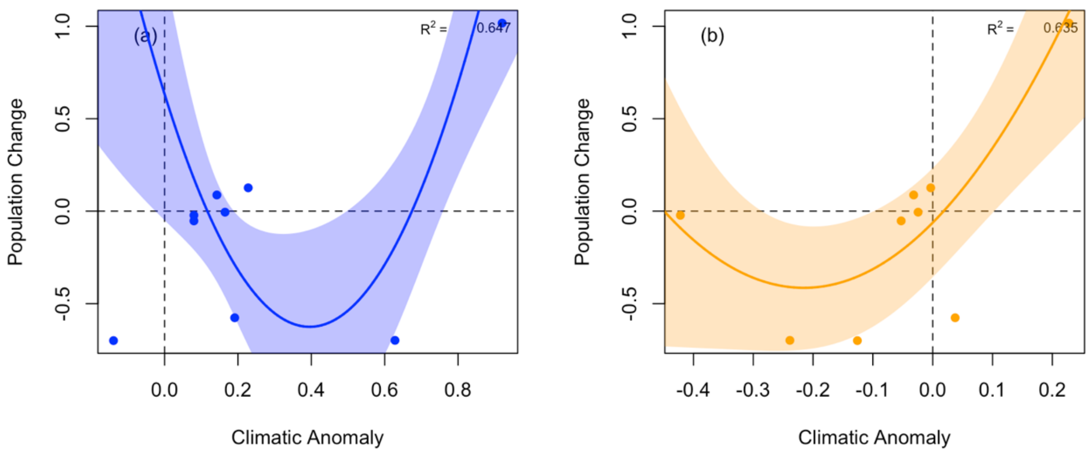

Supplementary Figure 76. Population change in relation to local and global climatic anomalies for (a) local and (b) global responses respectively for *Satyrium ilicis*, a species best adapted to local climatic anomalies in temperature during the pre-flight period of the year (t) of their adult stage. Colors indicate spatial scale (blue, local; orange, global), circles indicate raw data. R^2 values are provided.

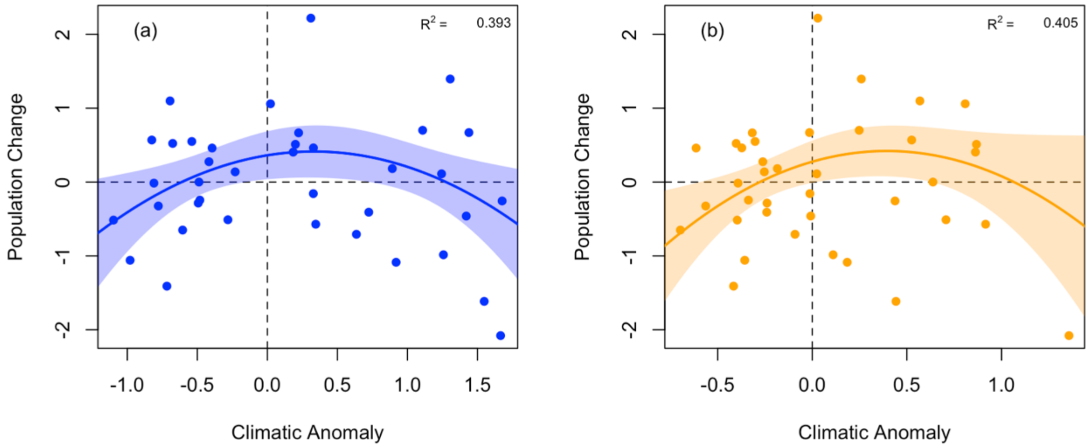

Supplementary Figure 77. Population change in relation to local and global climatic anomalies for (a) local and (b) global responses respectively for *Satyrium pruni*, a species best adapted to global climatic anomalies in temperature during the post flight period of the previous year (t-1) of their adult stage. Colors indicate spatial scale (blue, local; orange, global), circles indicate raw data. R^2 values are provided.

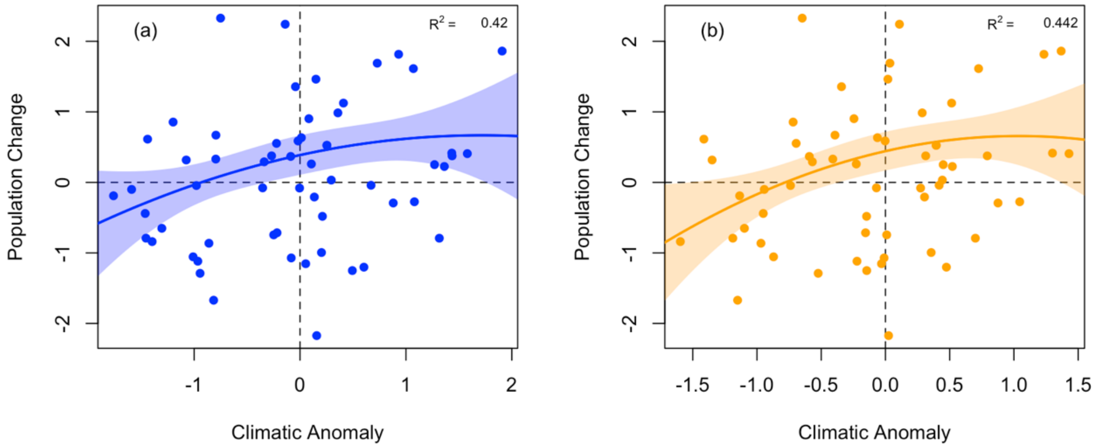

Supplementary Figure 78. Population change in relation to local and global climatic anomalies for (a) local and (b) global responses respectively for *Satyrium spini*, a species best adapted to global climatic anomalies in precipitation during the post flight period of the previous year (t-1) of their adult stage. Colors indicate spatial scale (blue, local; orange, global), circles indicate raw data. R^2 values are provided.

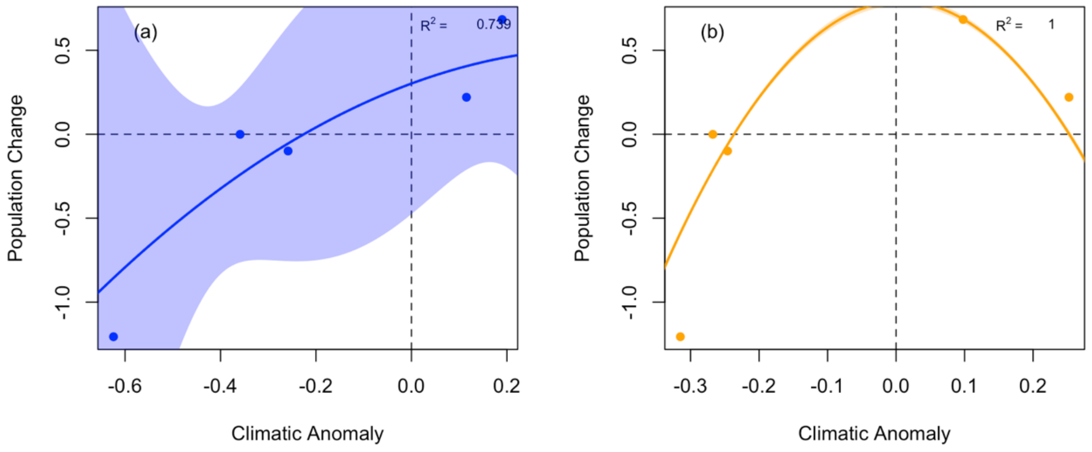

Supplementary Figure 79. Population change in relation to local and global climatic anomalies for (a) local and (b) global responses respectively for *Satyrium w-album*, a species best adapted to global climatic anomalies in precipitation during the flight period of the year (t) of their adult stage. Colors indicate spatial scale (blue, local; orange, global), circles indicate raw data. R^2 values are provided.

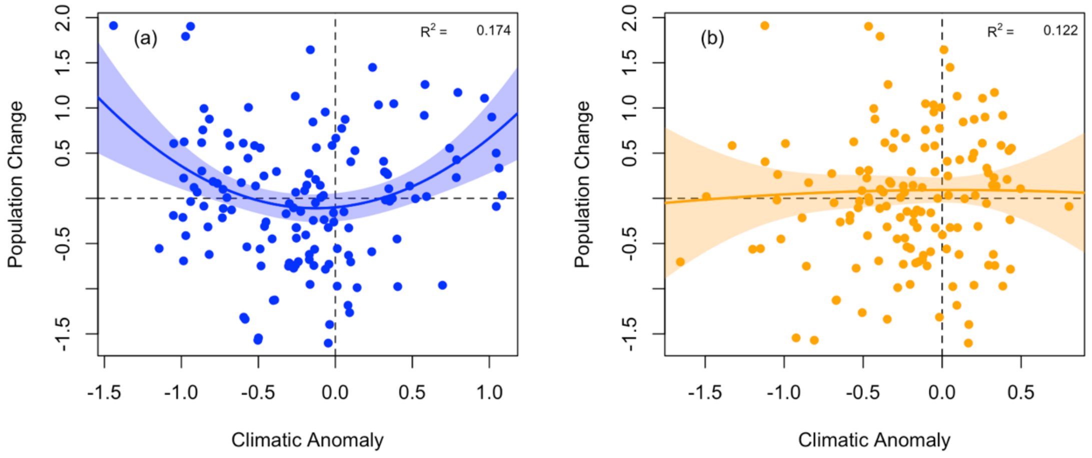

Supplementary Figure 80. Population change in relation to local and global climatic anomalies for (a) local and (b) global responses respectively for *Spialia sertorius*, a species best adapted to local climatic anomalies in temperature during the pre-flight period of the previous year (t-1) of their adult stage. Colors indicate spatial scale (blue, local; orange, global), circles indicate raw data. R^2 values are provided.

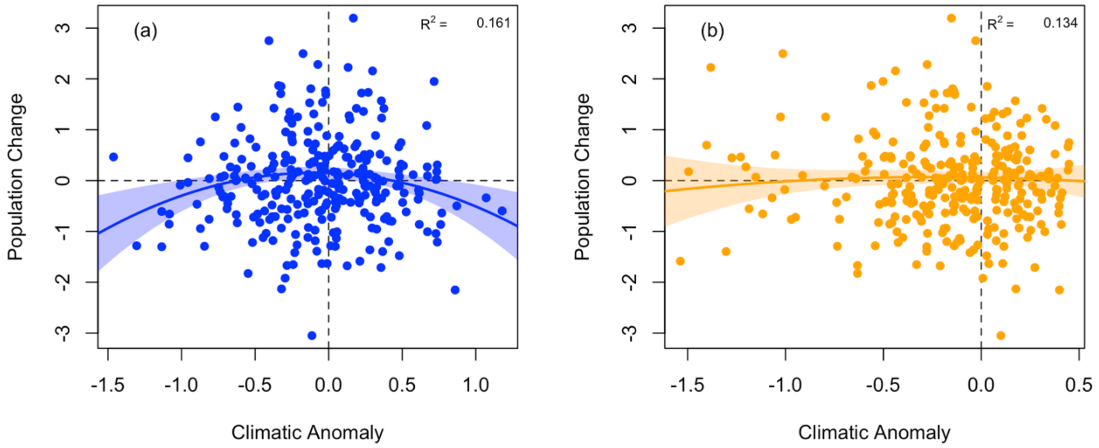

Supplementary Figure 81. Population change in relation to local and global climatic anomalies for (a) local and (b) global responses respectively for *Thymelicus acteon*, a species best adapted to local climatic anomalies in temperature during the pre-flight period of the previous year (t-1) of their adult stage. Colors indicate spatial scale (blue, local; orange, global), circles indicate raw data. R^2 values are provided.

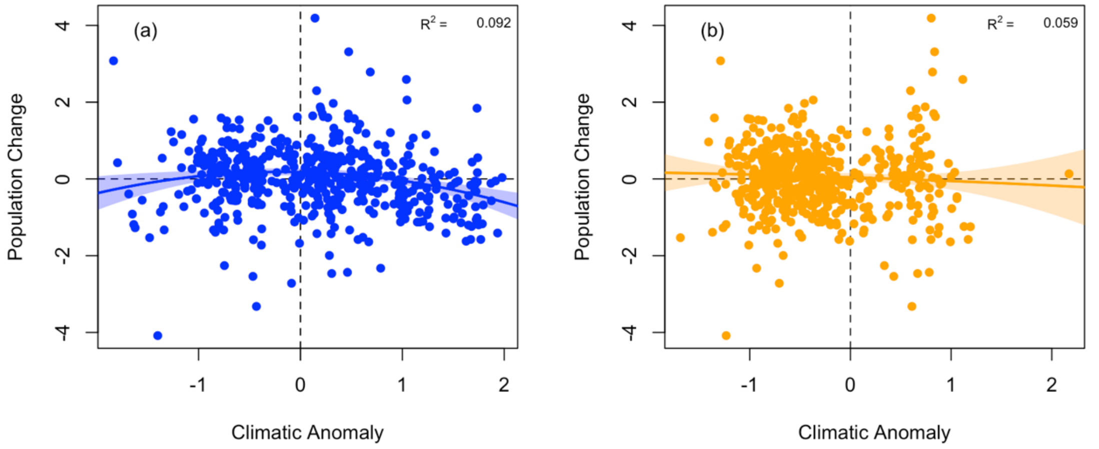

Supplementary Figure 82. Population change in relation to local and global climatic anomalies for (a) local and (b) global responses respectively for *Thymelicus lineola*, a species best adapted to local climatic anomalies in temperature during the post flight period of the previous year (t-1) of their adult stage. Colors indicate spatial scale (blue, local; orange, global), circles indicate raw data. R^2 values are provided.

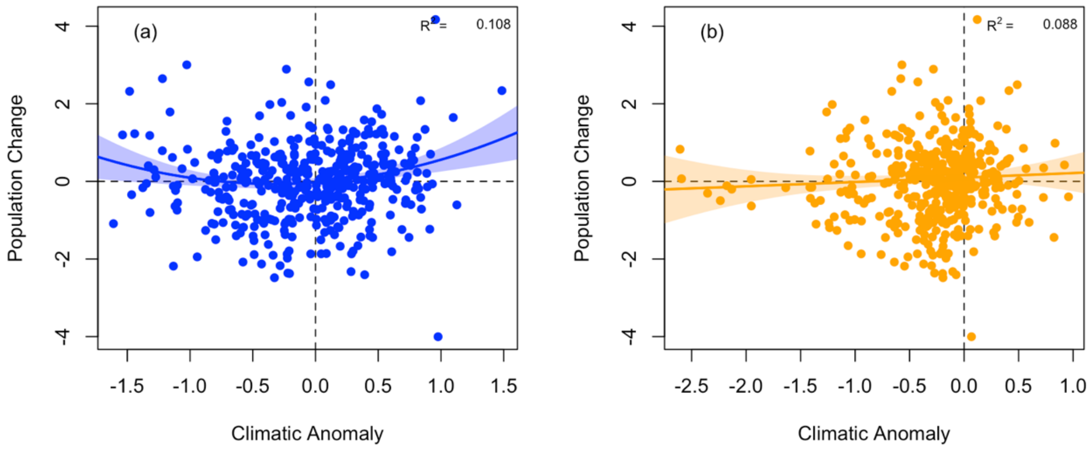

Supplementary Figure 83. Population change in relation to local and global climatic anomalies for (a) local and (b) global responses respectively for *Thymelicus sylvestris*, a species best adapted to local climatic anomalies in temperature during the pre-flight period of the previous year (t-1) of their adult stage. Colors indicate spatial scale (blue, local; orange, global), circles indicate raw data. R^2 values are provided.

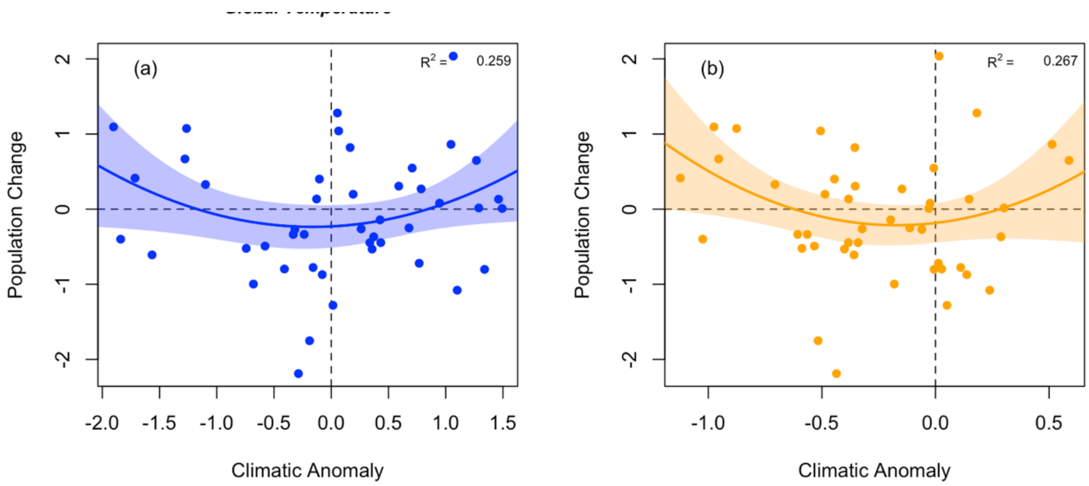

Supplementary Figure 84. Population change in relation to local and global climatic anomalies for (a) local and (b) global responses respectively for *Tomares ballus*, a species best adapted to global climatic anomalies in temperature during the flight period of the previous year (t-1) of their adult stage. Colors indicate spatial scale (blue, local; orange, global), circles indicate raw data. R^2 values are provided.

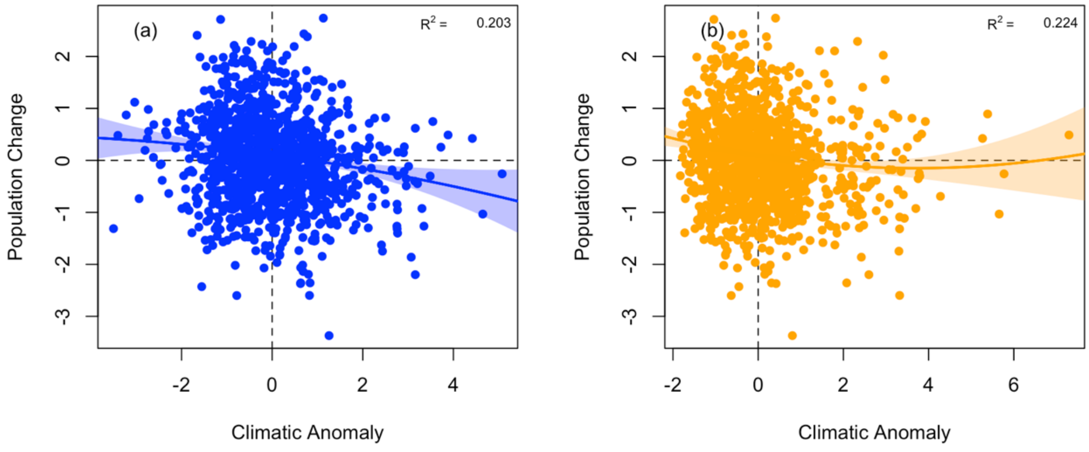

Supplementary Figure 85. Population change in relation to local and global climatic anomalies for (a) local and (b) global responses respectively for *Vanessa atalanta*, a species best adapted to global climatic anomalies in precipitation during the flight period of the previous year (t-1) of their adult stage. Colors indicate spatial scale (blue, local; orange, global), circles indicate raw data. R^2 values are provided.

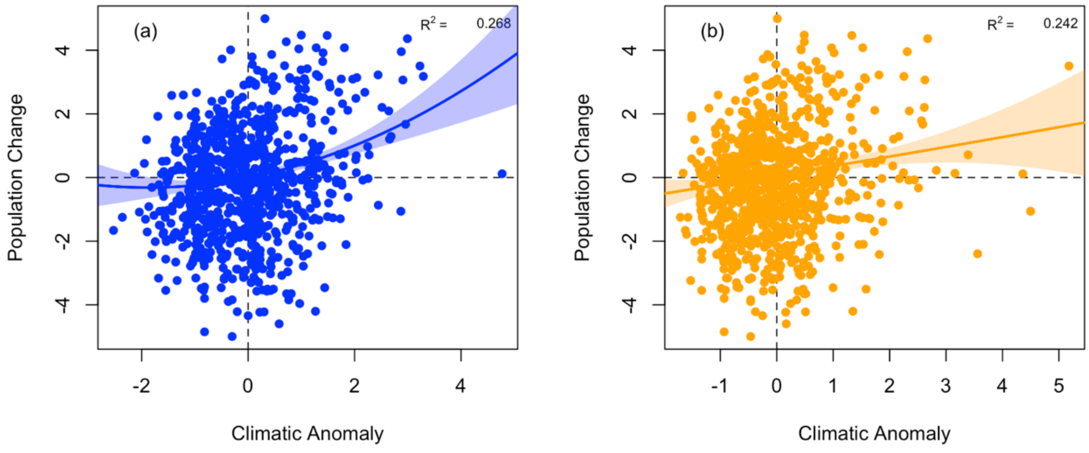

Supplementary Figure 86. Population change in relation to local and global climatic anomalies for (a) local and (b) global responses respectively for *Vanessa cardui*, a species best adapted to local climatic anomalies in precipitation during the flight period of the previous year (t-1) of their adult stage. Colors indicate spatial scale (blue, local; orange, global), circles indicate raw data. R^2 values are provided.

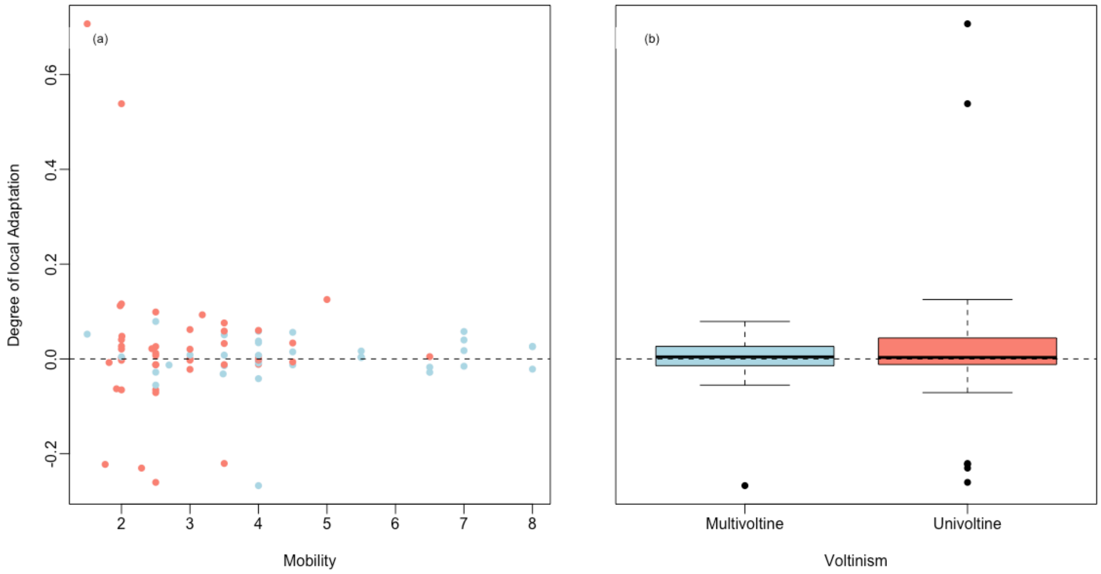

Supplementary Figure 87. Degree of local adaptation per species (dots) in relation to their (a) mobility and (b) voltinism for the 86 species for which climate was important in explaining population dynamics (i.e. the model with climatic anomalies was a significant improvement compared with the null model). The sample sizes per species range from 5 to 729 site-year combinations. Mobility is shown for 84 species since there is no data for two species (*Leptidea juvernica* and *Erebia euryale*), and voltinism for the 86 species.

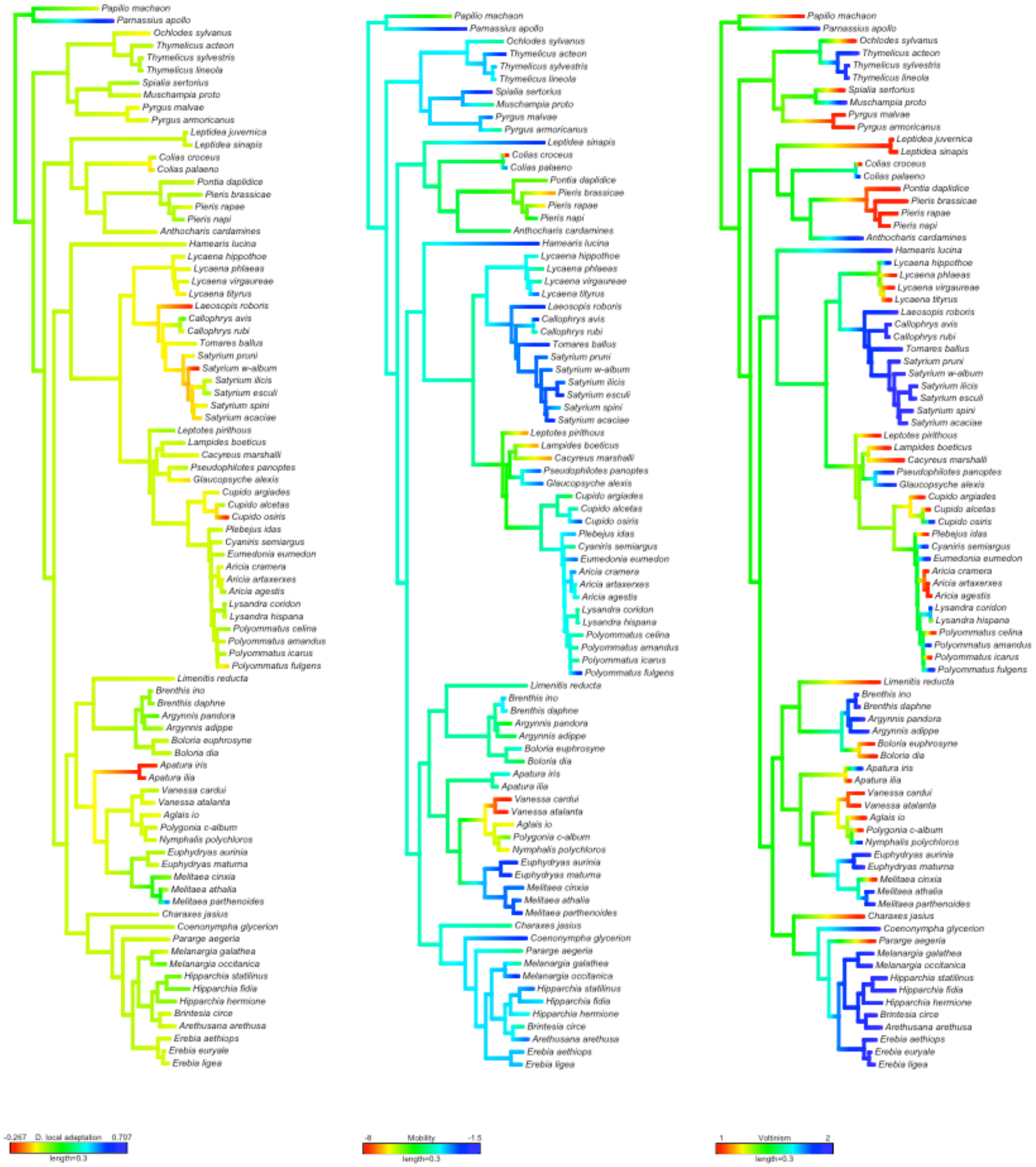

Supplementary Figure 88. Phylogenetic comparative trait plots of the butterfly traits for the species degree local adaptation, mobility (both with a continuous distribution) and voltinism (with a discrete distribution), for the 86 species for which climate was important in explaining population dynamics (i.e. the model with climatic anomalies was a significant improvement compared with the null model). Negative (red) values of the degree of local adaptation relate to species adapted to climatic anomalies at the global scale, positive values (blue) the local scale. Values of mobility are shown in the inverse order to coincide with the hypotheses for the degree of local adaptation; thus, negative (red) values relate to most mobile and positive (blue) values to less mobile species. Voltinism is marked as univoltine (red, 1) and multivoltine species (blue, 2).

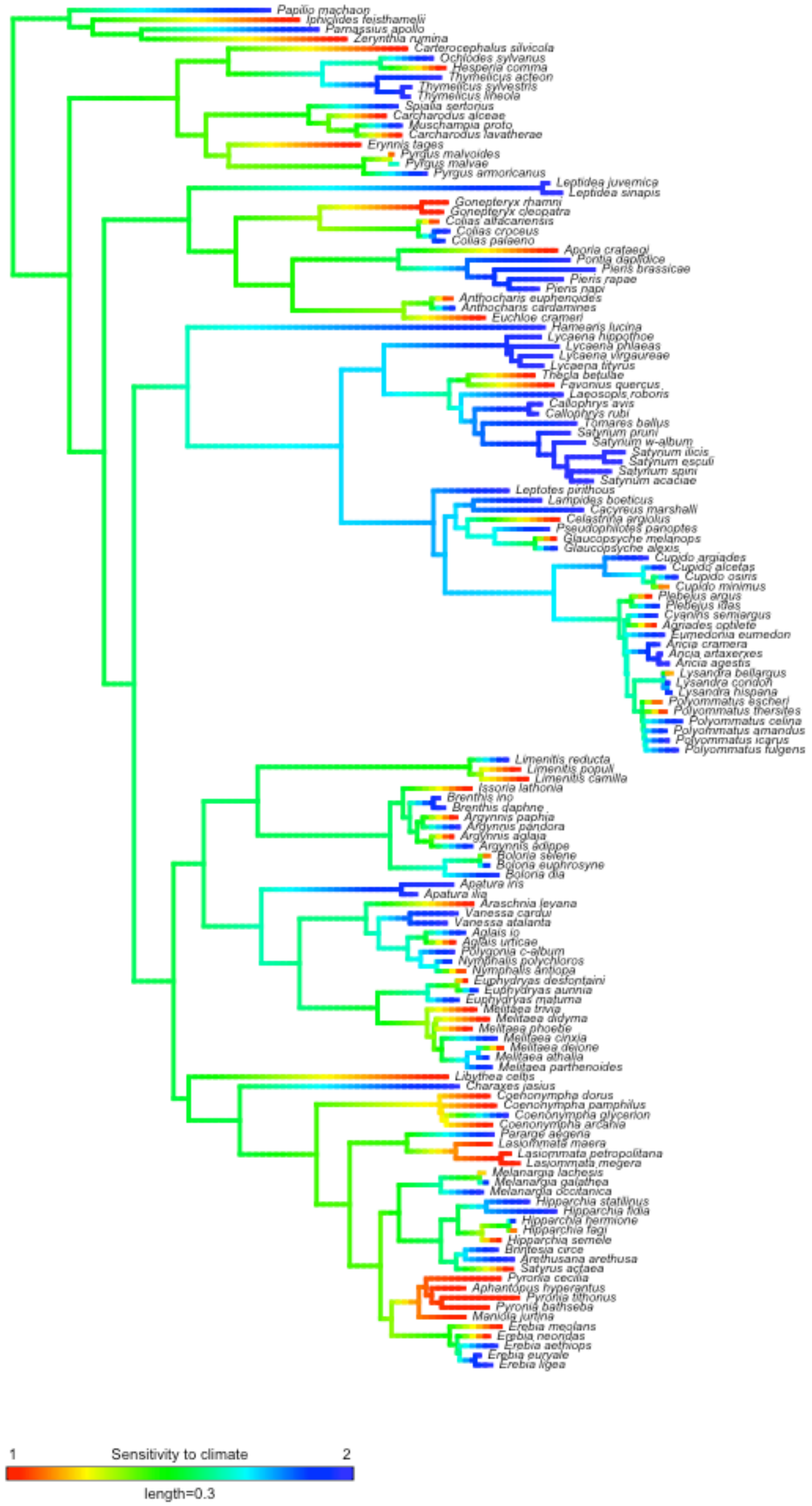

Supplementary Figure 89. Phylogenetic comparative trait plot of the butterfly sensitivity to climatic anomalies (with a discrete binomial distribution), for the 86 species for which climate was important in explaining population dynamics (i.e. the model with climatic anomalies was a significant improvement compared with the null model) and the 57 species for which density dependence but explained alone the population dynamics (i.e. the null model was the best model). No sensitivity is marked as red (1) and sensitivity as blue (2).

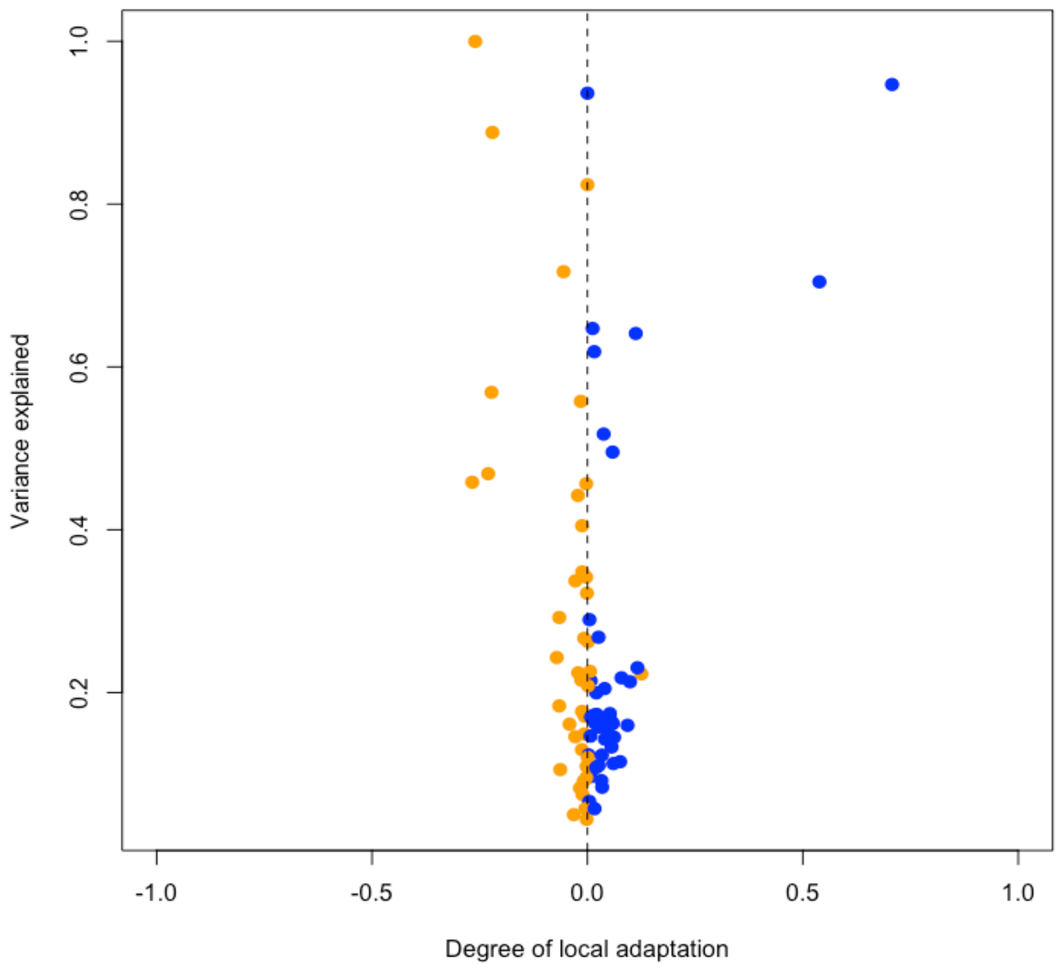

Supplementary Figure 90. Degree of local adaptation for the 86 species affected by climatic anomalies in relation to the variance explained (R^2) by the best model. No significant relationship was found (p-value = 0.5).

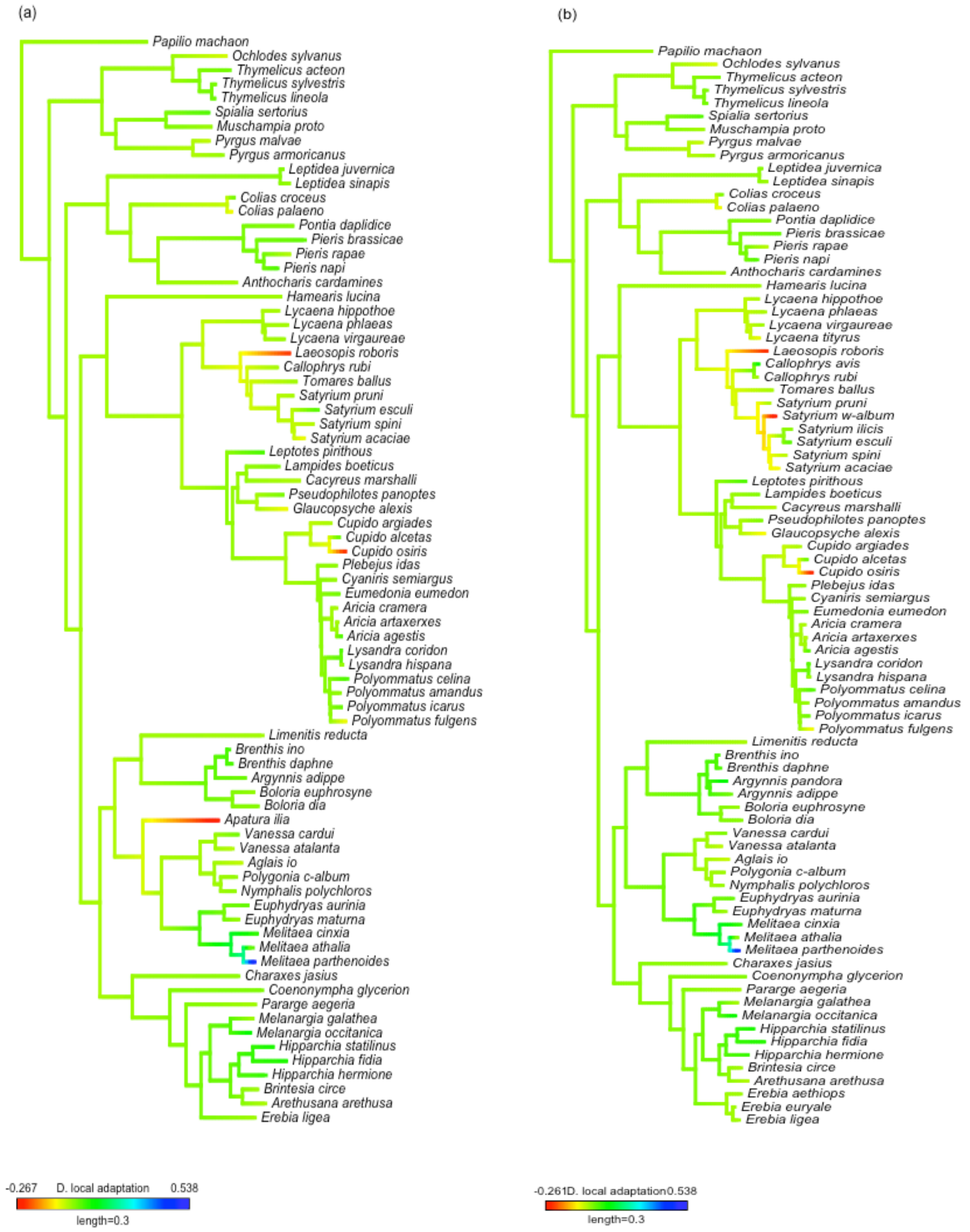

Supplementary Figure 91. Phylogenetic comparative trait plots of the butterfly traits for the species degree local adaptation, mobility (both with a continuous distribution) and voltinism (with a discrete distribution), for (a) the 77 species with Nsample size > 10, Pagel's lamda = 0.0007; and b) the 83 species left after removing visual outliers: Parnassius apollo, Apatura ilia and Aptaura iris, Pagel's lamda = 0.085. Negative (red) values of the degree of local adaptation relate to species adapted to climatic anomalies at the global scale, positive values (blue) the local scale.

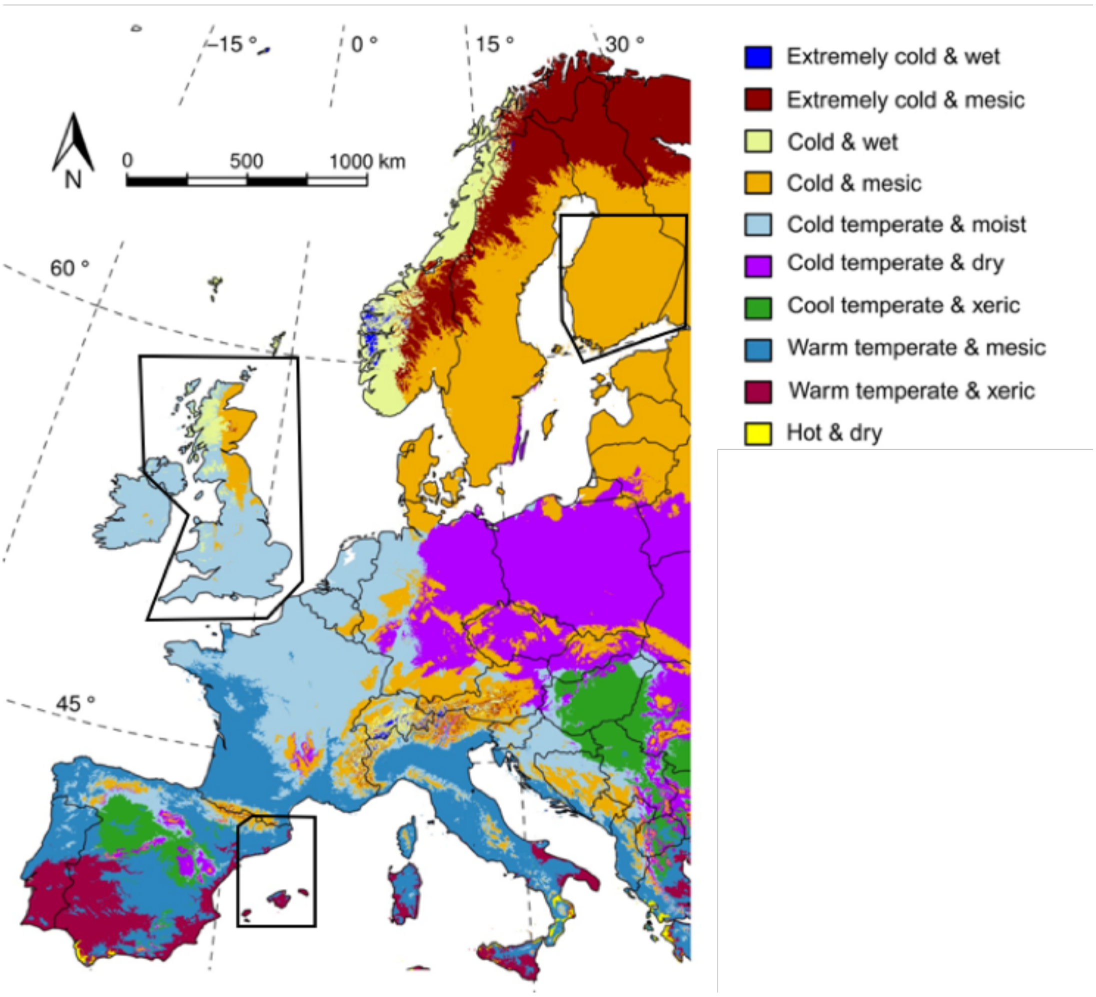

Supplementary Figure 92. Bioclimatic regions across Europe, marking those areas covered by the study. Adapted from Schmucki et al. (2016)[1] and regions based on Metzger et al. (2013)[2].

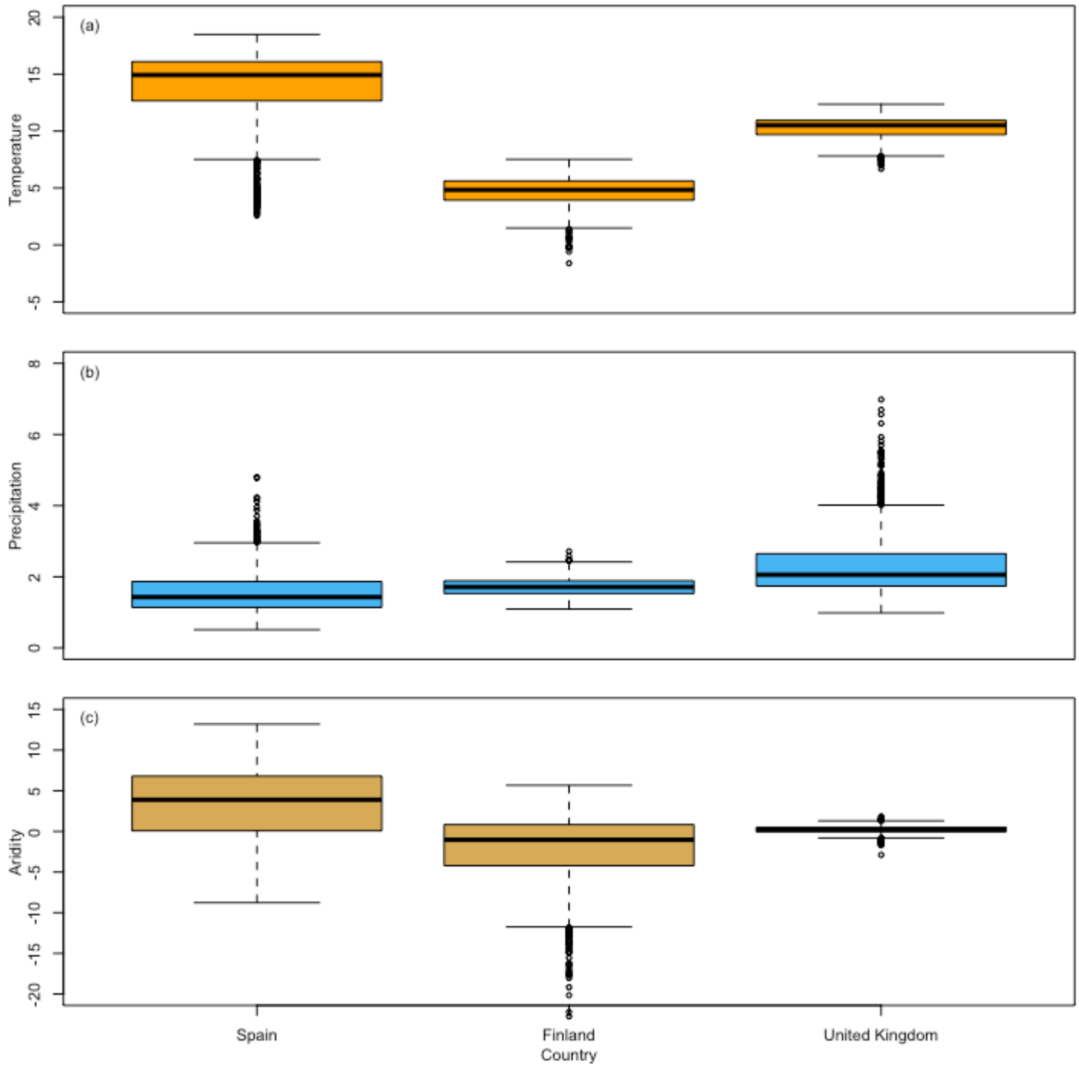

Supplementary Figure 93. Boxplot for the annual mean (a) temperature, (b) precipitation, and (c) aridity for the monitoring sites located in Spain, Finland and United Kingdom for the period 1999-2017.

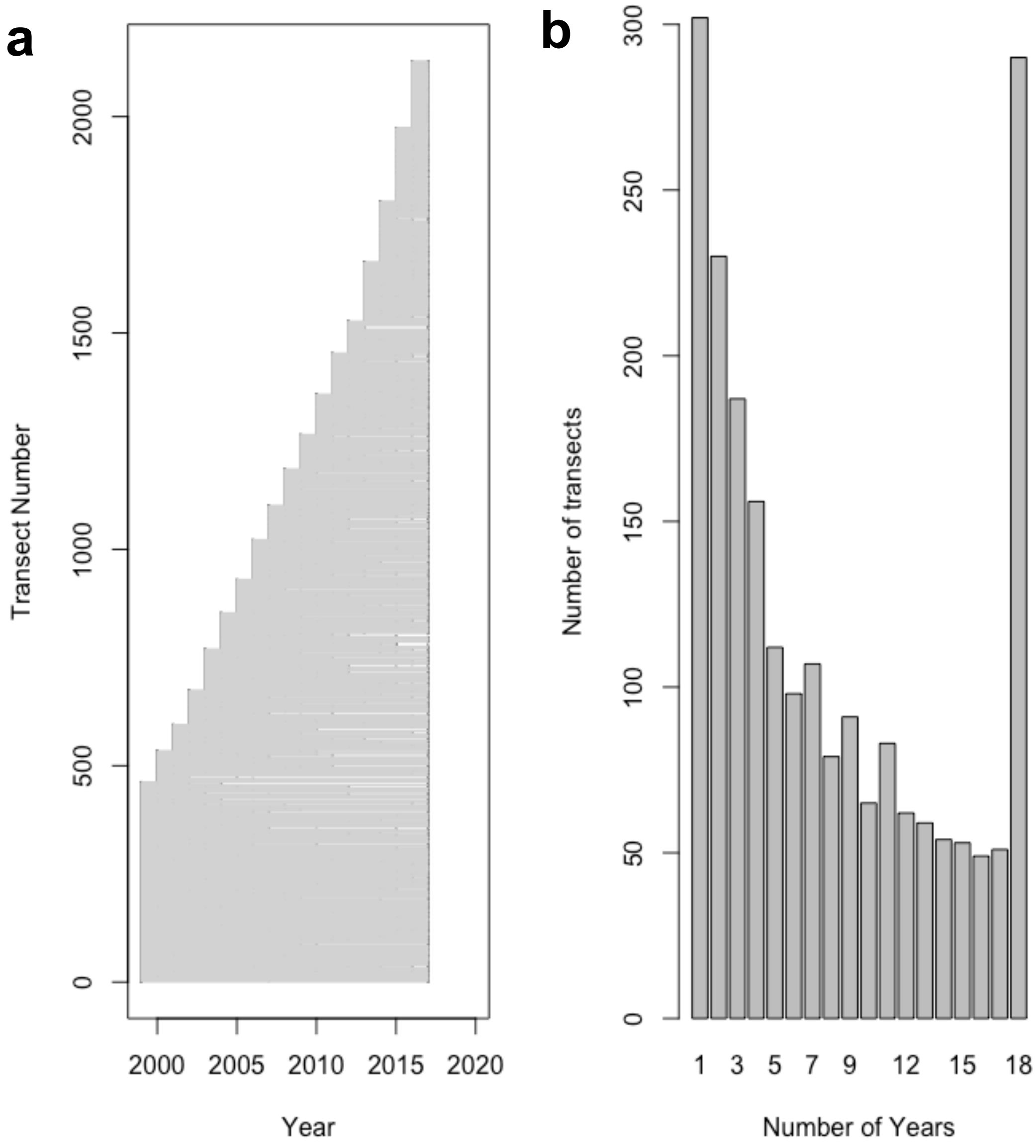

Supplementary Figure 94. (a) Duration of recording years that a transect has been surveyed in UK during 1999-2017; (b) Number of transects with a determined number of years of surveys carried out during 1999-2017; n = 2128.

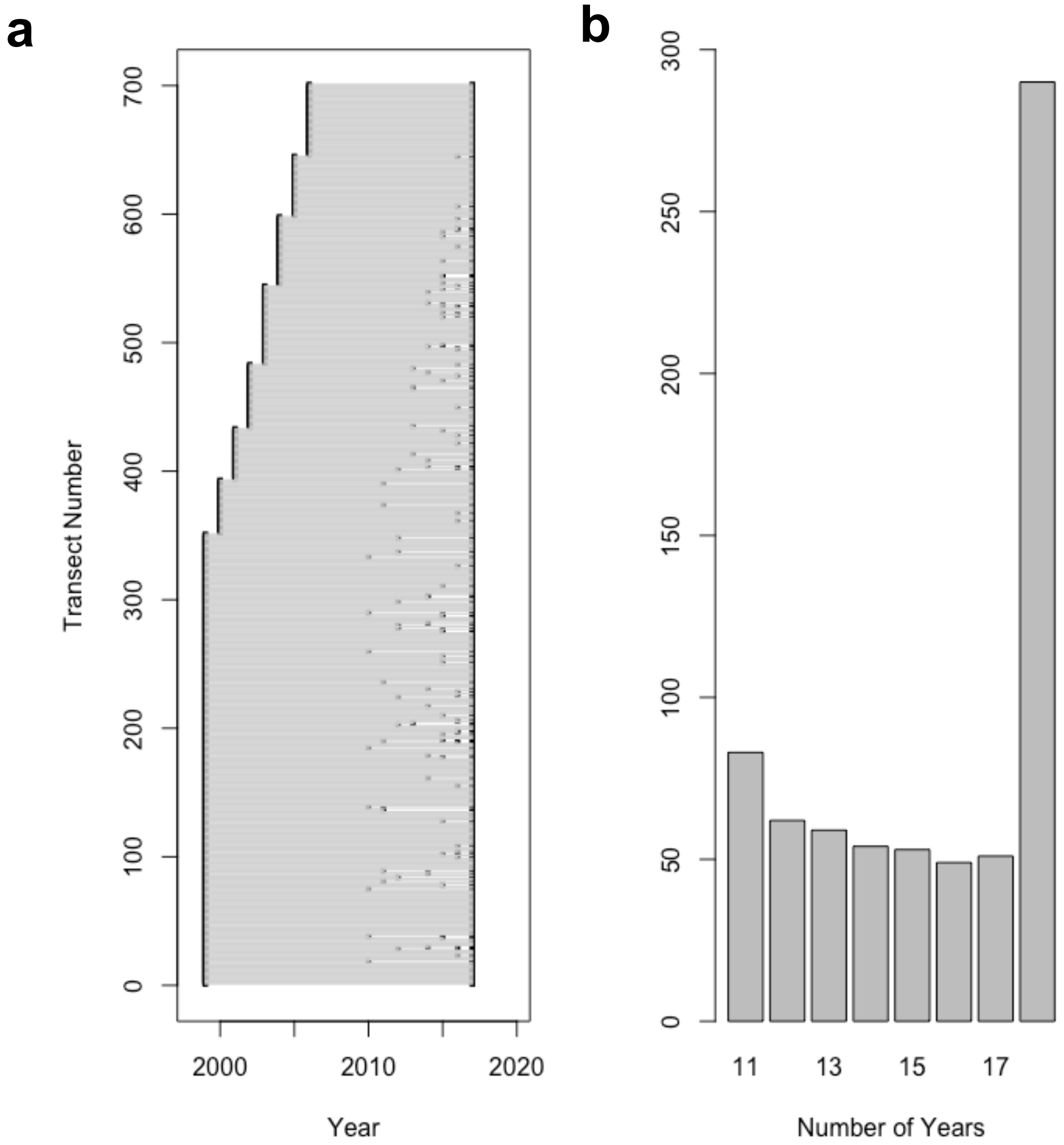

Supplementary Figure 95. (a) Duration of recording years that a transect has been surveyed in UK during 1999-2017, only for those transects with at least 10 years of recording; (b) Number of transects at least 10 years (or more) of population change data (at least two years of consecutive surveys) carried out during 1999-2017; n = 701.

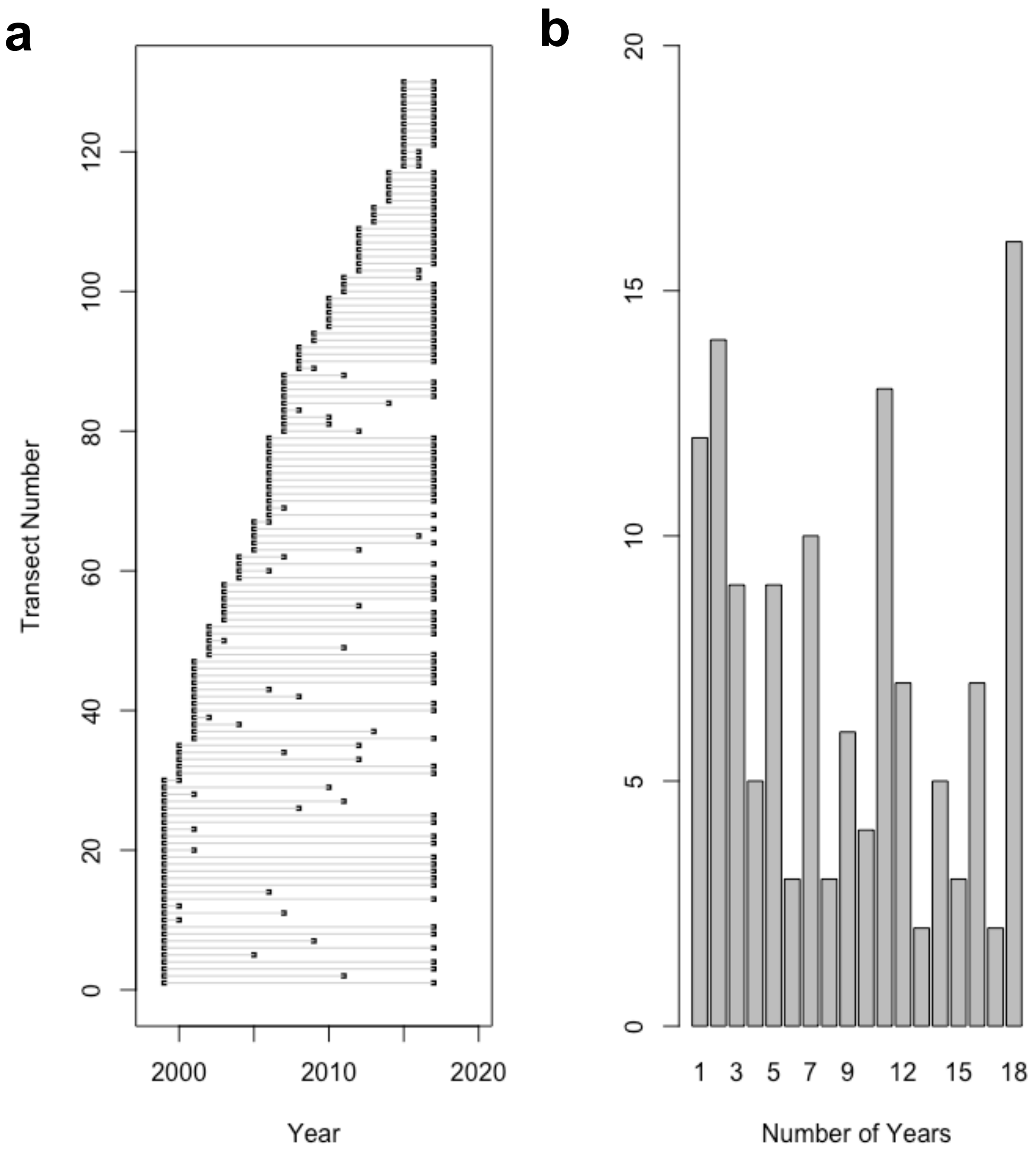

Supplementary Figure 96. (a) Duration of recording years that a transect has been surveyed in Catalonia during 1999-2017, for all transects; (b) Number of transects with a determined number of years of surveys carried out during 1999-2017; n = 130.

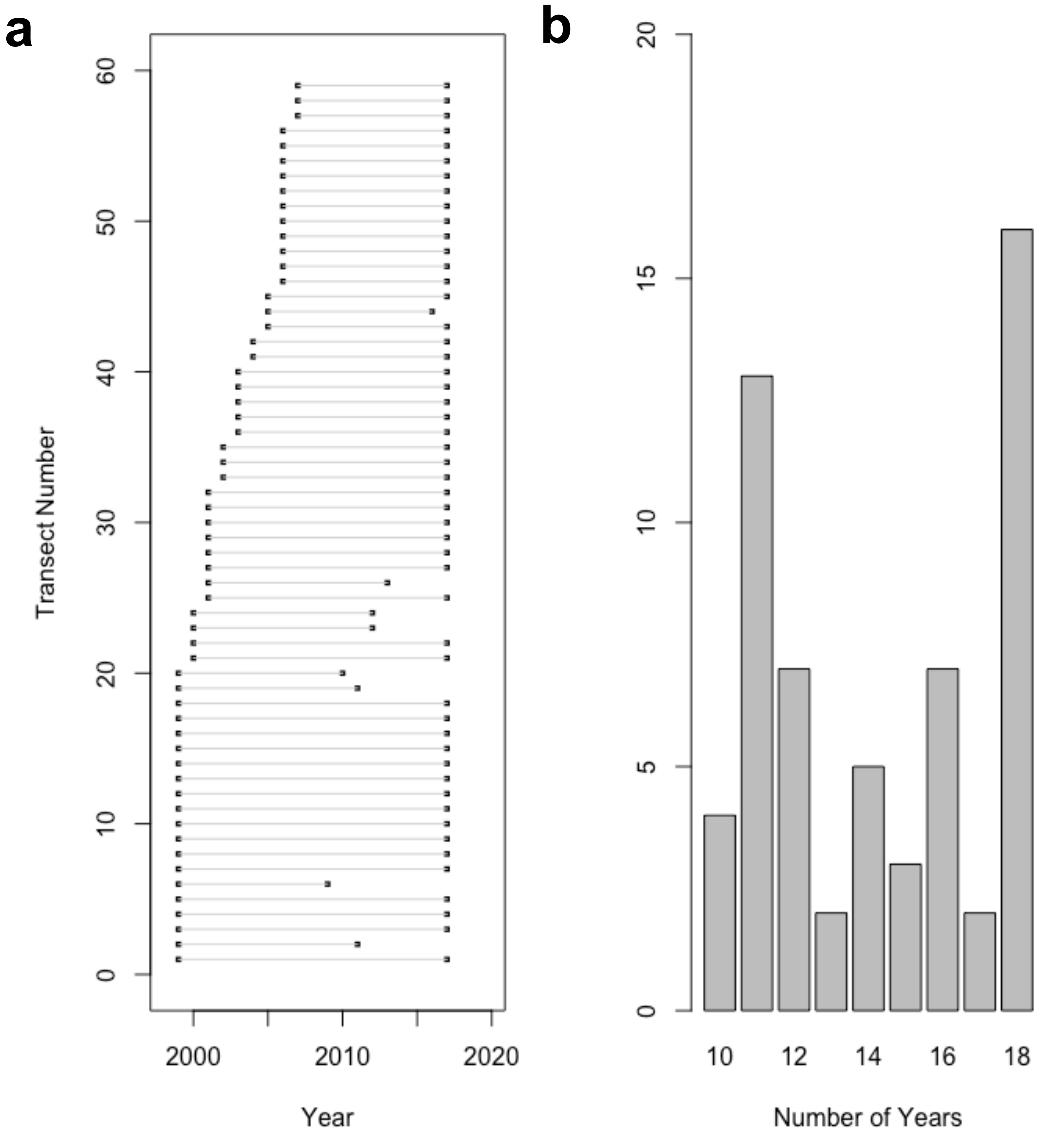

Supplementary Figure 97. (a) Duration of recording years that a transect has been surveyed in Catalonia during 1999-2017, only for those transects with at least 10 years of recording; (b) Number of transects at least 10 years (or more) of population change data (at least two years of consecutive surveys) carried out during 1999-2017; n = 59.

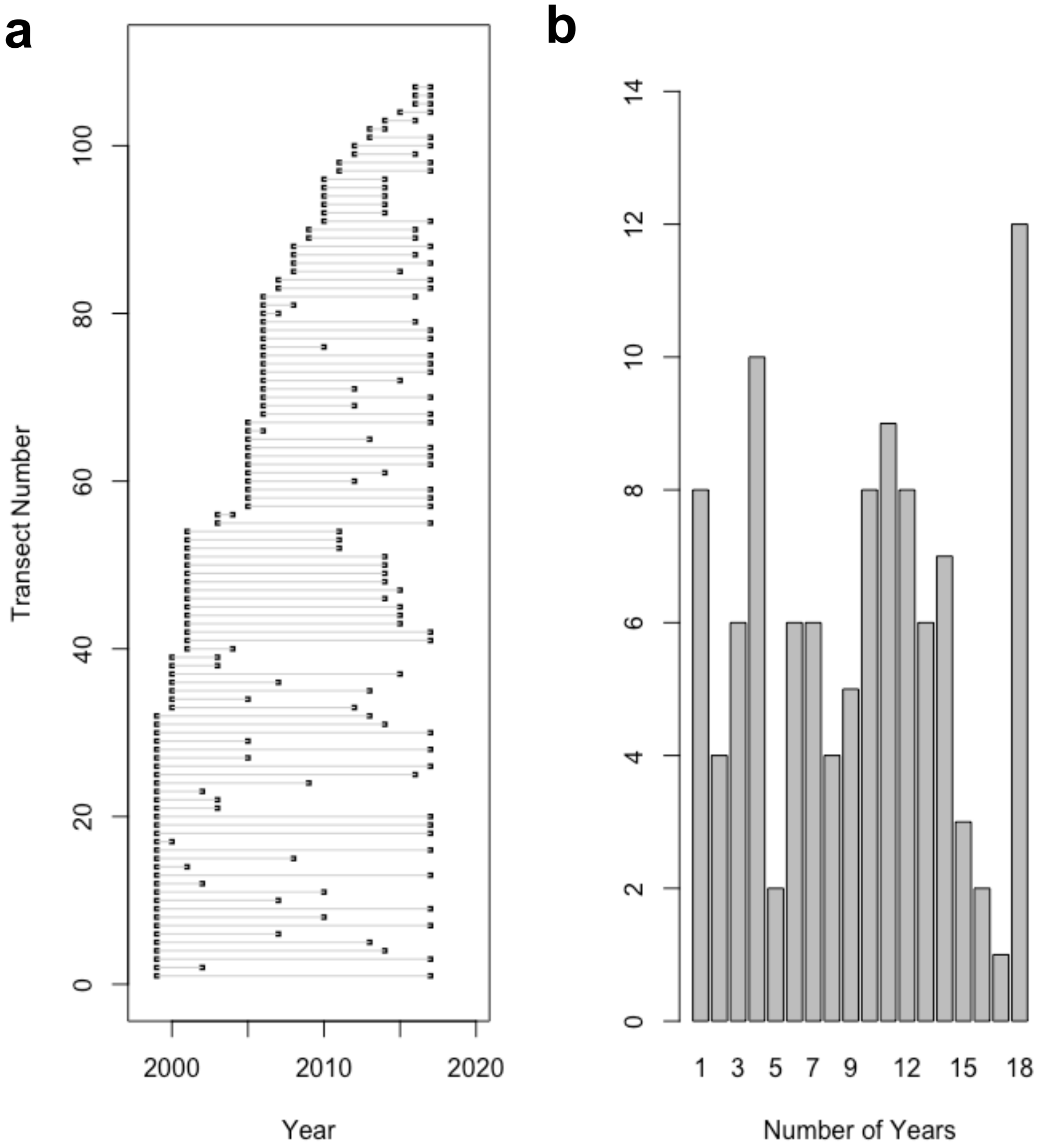

Supplementary Figure 98. (a) Duration of recording years that a transect has been surveyed in Finland during 1999-2017, for all transects; (b) Number of transects with a determined number of years of surveys carried out during 1999-2017; n = 107.

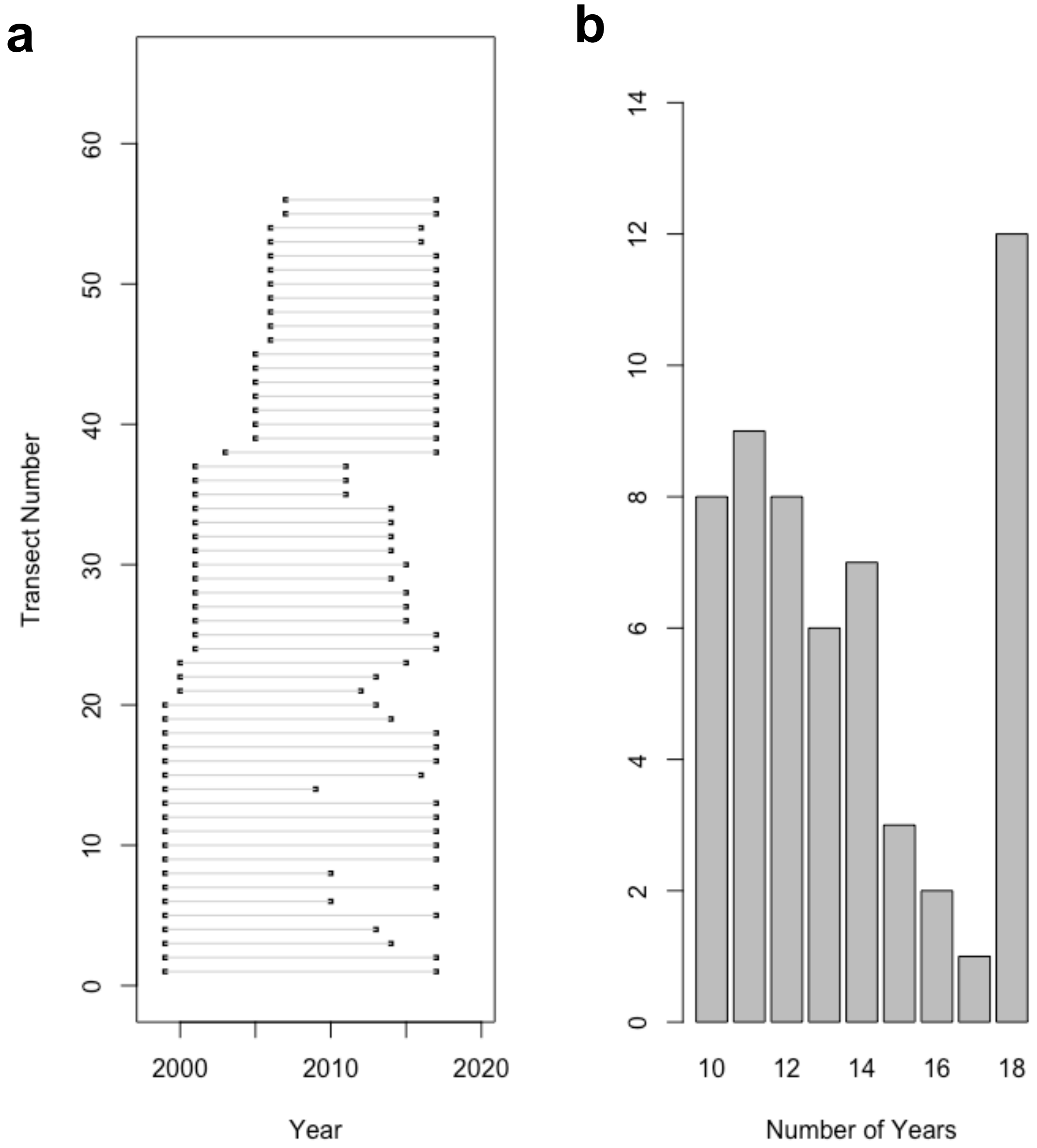

Supplementary Figure 99. (a) Duration of recording years that a transect has been surveyed in Finland during 1999-2017, only for those transects with at least nine years of recording; (b) Number of transects at least 10 years (or more) of population change data (at least two years of consecutive surveys) carried out during 1999-2017; n = 56.

Supplementary Table 1. Model parameter estimates for the influence of the species attributes, mobility and voltinism, on the degree of local adaptation to climatic anomalies, along with the phylogenetic signal in the degree local adaptation. The analysis includes 84 species affected by climatic anomalies since there were no data on mobility for two species (*Leptidea juvernica* and *Erebia euryale*). The intercept relates to the multivoltine species.

**Parameter**	**Estimate or Pagel's λ***	**SE**	**t-value**	**p-value**
Intercept	0.12	0.08	1.50	0.14
Univoltine	0.01	0.03	0.48	0.63
Mobility	0.0004	0.01	-0.04	0.97
Degree local adaptation	0.84	NA	NA	< 0.0001

* Phylogenetic signal of the degree of local adaptation

Supplementary Table 2. Phylogenetic signal of the butterfly species Degree local adaptation ($r^2_{local} - r^2_{global}$), mobility (both with a continuous distribution), voltinism (with a discrete distribution) and sensitivity to climatic anomalies (fit as a binary response). The Pagel's λ and D-statistic were used for continuous and discrete traits, respectively. Pagel's λ [0,1] close to 1 indicates strong phylogenetic signals. So does D-statistic near or below 0. For discrete traits the probability that the observed trait has a phylogenetically random distribution (i.e. no correlation between phylogenetic and trait distance) or follow a Brownian-motion (B-M) model of evolution (i.e. phylogenetic distance correlates with trait distance) are shown. For continuous traits, the significance of the phylogenetic signal, assessed using a likelihood-ratio test where H_0 is $\lambda = 0$, is shown. The Pagel's λ for the degree of local adaptation shown for the 86 species for which model with climatic anomalies improved their associate null model (sample size range 5-729). Mobility is shown for 84 species since there is no data for two species (*Leptidea juvernica* and *Erebia euryale*), and voltinism for the 86 species. Sensitivity to climatic anomalies is shown for all species set N = 143.

**Trait**	**Pagel's λ**	***P*-value**	**D-statistic**	**P (random) / P (B-M)**
Degree local adaptation	0.84	< 0.0001	NA	NA
Mobility	0.78	< 0.001	NA	NA
Voltinism	NA	NA	0.58	0.005 / 0.013
Sensitivity to climatic anomalies	NA	NA	0.88	0.14 / <0.0001

Supplementary Table 3. Model parameter estimates for the influence of the species attributes on the degree of local adaptation to climatic anomalies, along with the phylogenetic signal in the degree local adaptation; using (A) voltinism categorized as univoltine or multivoltine and maximum minus minimum number of generations[3], (B) voltinism categorized as univoltine, putative multivoltine or strict and maximum minus minimum number of generations[3], (C) voltinism categorized as univoltine, putative multivoltine or strict multivoltine[3], (D) voltinism categorized as specific number of generations (1, 1.5. 2 or >2)[3], (e) length of flight period (i.e. average number of flight months, FMo_Average) as a proxy of genetic differentiation instead of voltinism[3] and maximum minus minimum number of generations[3], (f) length of flight period as a proxy of genetic differentiation instead of voltinism[3], (g) voltinism categorized as univoltine or multivoltine, maximum minus minimum number of generations[3] and genetic differentiation (G_{ST}) instead of mobility as a proxy of gene flow[4], (h) voltinism categorized as univoltine or multivoltine and genetic differentiation (G_{ST})[4]. The analysis includes 79-84 species affected by climatic anomalies since there were no data on mobility for two species (*Leptidea juvernica* and *Erebia euryale*), on maximum and minimum number of generations for two other species and FMo_Average (*Argynnis adippe* and *Colias croceus*), and on G_{ST} for nine species (*Cacyreus marshalli*, *Callophrys avis*, *Erebia euryale*, *Euphydryas maturna*, *Leptidea juvernica*, *Polyommatus celina*, *Polyommatus fulgens*, *Satyrium pruni* and *Tomares ballus*). The intercept relates to the univoltine species, except in option (a) ad (g) where it relates to multivoltine as in Supplementary Table 1.

	**Parameter**	**Estimate or Pagel's λ***	**SE**	**t-value**	**p-value**
(a)	Intercept	0.11	0.09	1.25	0.21
	Univoltine	0.03	0.04	0.86	0.39
	Max - min	0.01	0.02	0.39	0.70
	Mobility	0.003	0.01	-0.30	0.76
	Degree local adaptation	0.84	NA	NA	< 0.0001
(b)	Intercept	0.11	0.09	1.25	0.21
	Putative Multivoltine	0.03	0.04	0.86	0.39
	Strict Multivoltine	0.01	0.02	0.39	0.70
	Max - min	0.00	0.01	-0.30	0.76
	Mobility	-0.01	0.01	-0.55	0.58
	Degree local adaptation	0.84	NA	NA	< 0.0001
(c)	Intercept	0.14	0.08	1.84	0.07
	Putative Multivoltine	0.02	0.03	0.51	0.61
	Strict Multivoltine	0.04	0.05	0.91	0.36
	Mobility	-0.01	0.01	-0.84	0.40
	Degree local adaptation	0.84	NA	NA	< 0.0001
(d)	Intercept	0.14	0.08	1.77	0.08
	Univoltine 1.5	-0.07	0.06	-1.07	0.29
	Multivoltine 2	0.01	0.04	0.22	0.82
	Multivoltine > 2	0.01	0.04	0.28	0.78
	Mobility	-0.01	0.01	-0.55	0.58
	Degree local adaptation	0.84	NA	NA	< 0.0001

(e)	Intercept	0.14	0.08	1.71	0.09
	FMo_Average	0.001	0.01	0.13	0.90
	Max - min	0.003	0.02	-0.15	0.88
	Mobility	-0.01	0.01	-0.60	0.55
	Degree local adaptation	0.84	NA	NA	< 0.0001
(f)	Intercept	0.14	0.08	1.73	0.09
	FMo_Average	0.0006	0.01	0.07	0.95
	Mobility	-0.01	0.01	-0.58	0.56
	Degree local adaptation	0.84	NA	NA	< 0.0001
(g)	Intercept	0.10	0.08	1.31	0.19
	Univoltine	0.03	0.03	1.00	0.32
	Max - min	0.01	0.02	0.42	0.67
	G_{ST}	-0.01	0.05	-0.23	0.82
	Degree local adaptation	0.84	NA	NA	< 0.0001
(h)	Intercept	0.11	0.07	1.57	0.12
	Univoltine	0.03	0.03	0.92	0.36
	G_{ST}	-0.01	0.05	-0.22	0.82
	Degree local adaptation	0.84	NA	NA	< 0.0001

* Phylogenetic signal of the degree of local adaptation

Supplementary References

1. Schmucki, R. *et al.* A regionally informed abundance index for supporting integrative analyses across butterfly monitoring schemes. *J. Appl. Ecol.* **53**, 501–510 (2016).
2. Metzger, M. J. *et al.* A high-resolution bioclimate map of the world: a unifying framework for global biodiversity research and monitoring. *Glob. Ecol. Biogeogr.* **22**, 630–638 (2013).
3. Middleton-Welling, J. *et al.* A new comprehensive trait database of European and Maghreb butterflies, Papilionoidea. *Sci. Data* **7**, (2020).
4. Dapporto, L. *et al.* Integrating three comprehensive data sets shows that mitochondrial DNA variation is linked to species traits and paleogeographic events in European butterflies. *Mol. Ecol. Resour.* **19**, 1623–1636 (2019).
