# Supplementary material for: Local adaptation to climate anomalies relates to species phylogeny
Source: Commun Biol. 2022 Feb 17;5:143. doi: 10.1038/s42003-022-03088-3 (PMC8854402; doi:10.1038/s42003-022-03088-3)
Supplement: Supplementary file 3 — Description of Additional Supplementary Files [file 42003_2022_3088_MOESM3_ESM.pdf]

Description of Additional Supplementary Files

File name: Supplementary Data 1
Description: Models AIC and rsquared marginal and conditional per species, time, period and climatic anomaly at the local and global scale. Best models per species are marked in bold.

File name: Supplementary Data 2
Description: Best model per Page 7 of 13 species and related variables per species.
